# Submillennial timescales of eruptible rhyolite magma: Converging evidence from Aira Caldera, Japan

**DOI:** 10.1126/sciadv.aea2075

**Published:** 2026-08-07

**Authors:** Bradley W. Pitcher, Guilherme A. R. Gualda, Nobuo Geshi

**Affiliations:** ^1^ Earth & Environmental Sciences, Vanderbilt University, Nashville, TN 37235, USA.; ^2^ Lamont-Doherty Earth Observatory, Columbia University, Palisades, NY 10964, USA.; ^3^ Geological Survey of Japan, AIST, AIST Site 7, 1-1-1 Higashi, Tsukuba, Ibaraki 305-8567, Japan.; ^3^ Department of Earth and Planetary Sciences, Kyushu University, 744-W1 Motooka, Nishiku, Fukuoka 819-0395, Japan.

## Abstract

Recent studies debate whether caldera-forming silicic magma is primarily stored in a crystal-poor, eruptible state for >100,000 years (kyr) (“warm storage”) or exists as a crystal-rich “mush” with defrosting events causing brief periods (<1 kyr) of eruptibility (“cold storage”). To address this, we examined Aira Caldera (Japan), where the 400–cubic kilometer Aira-Tanzawa (AT) eruption (30,000 years ago) was preceded by three smaller eruptions over 3.5 kyr. All eruptions consist of roughly similar high-silica rhyolite stored at comparable depths (75 to 175 megapascals). Diffusion modeling in quartz and plagioclase is consistent and gives short residence times (<1 to 2.5 kyr) for all eruptions with no progressive increase up-section. The AT eruption also shows signs of hotter, less evolved magma and higher lithium concentrations, possibly indicating greater mush rejuvenation by a hotter fluid-rich recharge. We suggest that the 400–cubic kilometer AT magma was eruptible for less than 1 kyr, consistent with the hypothesis that silicic magmas are primarily held in cold storage with brief periods of eruptibility.

## Teaser

Multiple crystal clocks from Aira eruptions reveal short periods of melt-rich eruptibility, supporting the cold storage model.

## INTRODUCTION

Caldera-forming eruptions (CFEs) rapidly expel tremendous volumes of magma (up to thousands of cubic kilometers) and volatiles and therefore pose a serious threat to life, property, infrastructure, and even global climate. Thus, it is critical that we understand the timescales of the magmatic processes that lead to these catastrophic events. In the past two decades, there has been a major paradigm shift in our understanding of the storage conditions and timescales of silicic magmas that erupted at caldera systems worldwide. Recent work has challenged the traditional model held for the past century in which a single reservoir of magma persists in a melt-rich eruptible state for tens of thousands to millions of years before eruption (*1*, *2*). Instead, many recent studies have suggested a model in which caldera-forming magma is stored in a complex system throughout the crust (*3*–*6*) and is predominantly stored as crystal-rich mush bodies (*6*–*8*) that can be remobilized into melt-dominated, eruptible bodies over timescales of decades to centuries (*9*–*13*). This “cold storage” model (*14*) elegantly explains an apparent discrepancy between chronometers: The long timescales from ^238^U-^206^Pb dating of accessory minerals [5 to 700 thousand years (kyr)] and ^238^U-^230^Th disequilibria of major phases (1 to 300 kyr) represent the total residence time, at any temperature, because the magma first began crystallizing, whereas the much shorter mineral diffusion timescales (10 to 1000 years) represent the total time that the magmatic systems exist at elevated temperatures capable of producing large volumes of eruptible magma (i.e., above the rheological lock-up temperature). This implies that a given magma system resides in a noneruptible crystal-rich state for more than 90 to 99.9% of the total time (*14*), consistent with low melt fractions observed with geophysical imaging for systems worldwide (*15*–*17*).

Two recent papers, however, have argued that titanium diffuses two to three orders of magnitude slower in quartz than previously determined by Cherniak *et al.* (*18*), and therefore, a quartz diffusion timescale of 1 kyr found by numerous studies on caldera systems worldwide would actually be 1.2 or 3.9 million years (Myr) (*19*, *20*), respectively. A third study using U-series dating of zircon inclusions within quartz gives an intermediate diffusion coefficient that would give timescales of 0.4 Myr (*21*). Because these timescales, which give time at elevated temperatures, are similar to or longer than the total magma crystallization times (at any temperature) determined by absolute U-Pb dating of accessory phases (*22*), it would imply that melt-dominated eruptible magmas are perpetually held in “warm storage” for hundreds of thousands to millions of years before eruption. As a result, our understanding of the timescales that lead to CFEs has been cast into a state of uncertainty: Melt-rich eruptible magmas are either ephemeral and can be remobilized and accumulated within centuries leading to eruption or magma bodies reside in a melt-rich state beneath volcanoes for hundreds of thousands to millions of years before eruption. Considering the hazard monitoring implications for these drastically different models of eruptible magma storage timescales, resolving this debate is of critical importance for the field of volcano science.

**Fig. 1.**
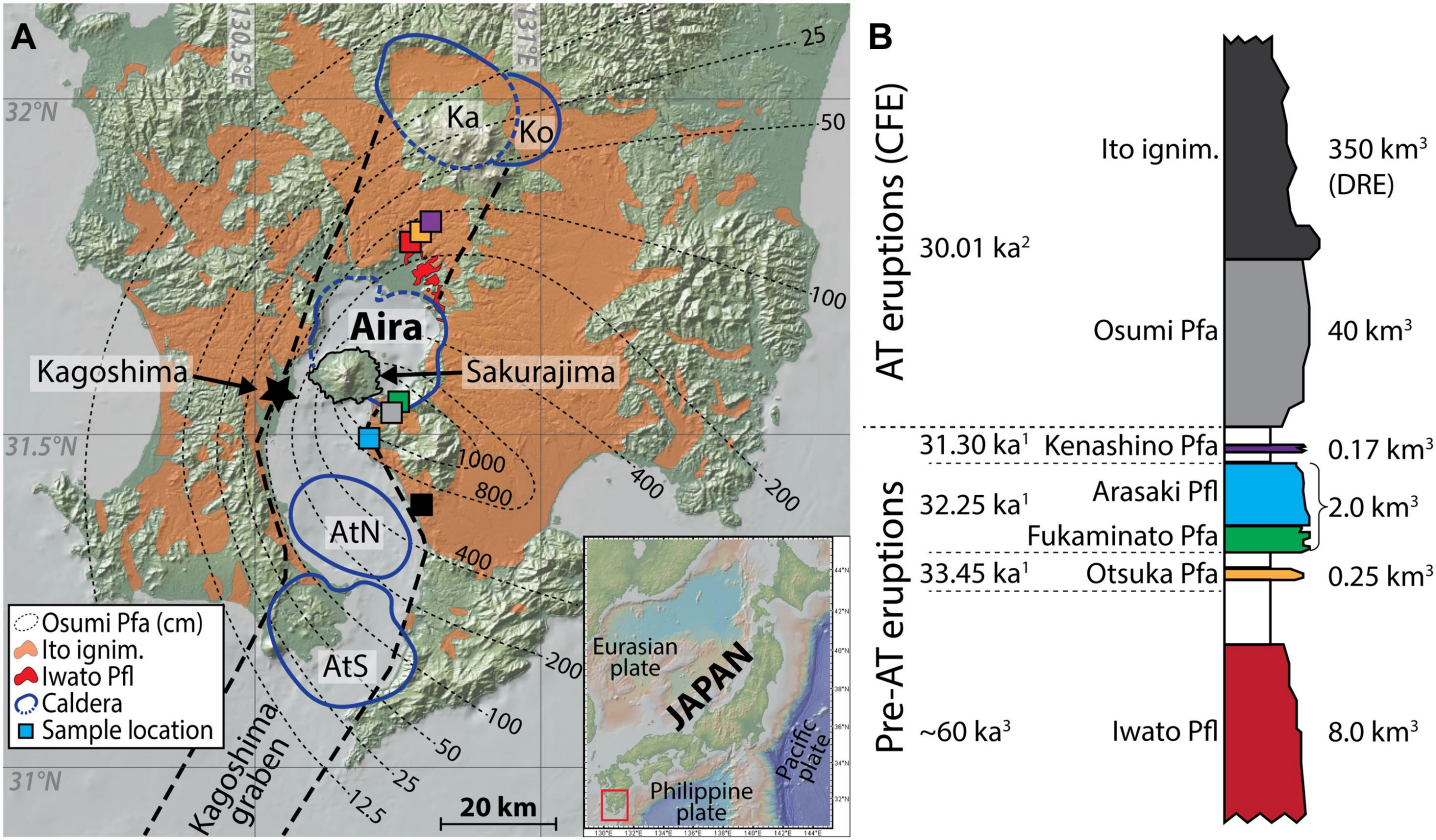
Map and simplified stratigraphic column depicting pre-AT and AT eruptions. (**A**) Map showing the location of Aira Caldera in southern Kyushu Japan and the deposits from the caldera-forming “AT” eruption: Orange regions show the distribution of the Ito ignimbrite, and dashed isopach lines represent the thickness (in cm) of the Osumi fall deposit (*23*). The locations of other major middle-late Pleistocene calderas within the Kagoshima Graben are shown. Ka, Kakuto; Ko, Kobayashi; AtN, Ata North; AtS, Ata South. Sampling locations for the samples used in this study are shown as boxes colored according to the stratigraphic section. (**B**) Schematic stratigraphic section of Aira pyroclastic fall (Pfa) and flow (Pfl) units. Thickness of pre-AT units are scaled relative to DRE volume, which are 8x vertically exaggerated compared to Osumi Pfa. The thickness of Ito and Iwato ignimbrites are cut off at the top and bottom, respectively. For simplicity, we omit the Tsumaya and Tarumizu Pfl deposits, which erupted between Osumi and Ito, because they were not studied in detail in this study. Stratigraphic profiles of each unit are modified from Nagaoka *et al.* (*24*). Approximate ages are displayed to the left, where (i) this study (^14^C of underlying soil), (ii) Smith *et al.* (*25*) (^14^C of correlated lacustrine ash), and (iii) Nagaoka *et al.* (*27*) (approximate age from stratigraphy). Color scheme of units within the stratigraphic section will be used for all figures in this paper.

Aira Caldera, in southern Kyushu, Japan, is the perfect case study to test the viability of these conflicting timescales. The 400-km^3^ CFE of Aira-Tanzawa (AT), [30 thousand years ago (ka)], was preceded, within 3.5 kyr, by three smaller pyroclastic eruptions (0.17 to 2.0 km^3^) (*23*, *24*), thereby providing a unique opportunity to assess the magmatic timescales leading to the CFEs (Fig. 1). Our test is this: If the longer diffusion timescales of hundreds of thousands of years are accurate, then the magma that fed each of the pre-AT and AT eruptions must have all been in an eruptible state simultaneously, implying protracted “warm storage.” However, if the shorter diffusion timescales of <1 kyr are more accurate, then each magma batch erupted, including that of the 400-km^3^ AT eruption, could have been in an eruptible state for a shorter time than the recurrence interval and would have been assembled rapidly after the previous eruption. In this study, we use major and trace elements in pumice glass and plagioclase and multiple geobarometers to assess the petrogenetic relationship between the pre-AT and AT magmas and use textural evidence and multiple independent mineral diffusion chronometers to assess whether the 400-km^3^ caldera-forming melt-rich magma was accumulated rapidly or was in an eruptible state, along with the pre-AT magmas, for much longer timescales. This work allows us to weigh in on the warm storage versus cold storage debate, which is critical to our understanding of magmatic processes at silicic systems worldwide.

### Aira Caldera background

Aira caldera (17 km by 23 km) is the largest of at least seven middle to late Pleistocene calderas within the Kagoshima graben (*23*, *24*). The NNE-SSW trending graben is 120 to 130 km long by 20 to 30 km wide (Fig. 1), and volcanic activity is the result of the subduction of the Philippine plate beneath the Eurasian plate. Aira caldera was formed during the AT eruption (30.009 ± 0.189 ka), which deposited tephra over most of Japan and had a total estimated volume of 400 km^3^ [dense rock equivalent (DRE)] (*24*, *25*). The AT eruption consisted of the Osumi pumice fall deposit (>27- to 31-km^3^ DRE), the Tarumizu pyroclastic flow (10 km^3^), Tsumaya pyroclastic flow (6 km^3^), and the 350-km^3^ Ito ignimbrite and corresponding co-ignimbrite ash (*23*, *26*). The eruption is considered one of the largest in the Northwest Pacific since the late Pleistocene (*23*). Before the AT eruption, there were five major pumice fall (Pfa) and pyroclastic flow (Pfl) eruptions since 100 ka, including the Fukuyama Pfa (not studied here), Iwato Pfl, Otsuka Pfa, Fukaminato Pfa + Arasaki Pfl, and Kenashino Pfa and maar deposit (*24*, *27*) (Fig. 1).

Following the AT eruption, Aira experienced numerous post–caldera eruptions, including the formation of Sakurajima on the southern caldera rim, which has built a 25-km^3^ stratovolcanic edifice since 26 ka (*23*). Over 17 Plinian and sub-Plinian eruptions from Sakurajima have occurred in this time, and it continues to erupt daily, posing a threat to the roughly 1.6 million people who live in the Kagoshima prefecture surrounding it (*28*). The magmatic system that formed the caldera is still active, as evidenced by numerous geophysical studies that indicate magmatic storage for Sakurajima located 8 km north of the volcanic edifice, directly below the center of the caldera (*29*).

## RESULTS

A summary of petrographic features, compositions, timescales, and geobarometry results for each unit is given in Table 1.

**Table 1.** Summary table comparing important attributes of each statistically distinct pumice population. The table compares the petrography, major and trace element glass composition, rhyolite-MELTS pressure, MI H_2_O content, MI H_2_O-CO_2_ saturation pressure, zircon saturation temperature, and maximum quartz residence time [using the diffusion coefficient of Cherniak *et al.* (*18*)]. Eruption ages (± 2σ) are from this study (^14^C of underlying soil), except for (a) Nagaoka *et al.* (*27*) (approximate age from stratigraphy) and (b) Smith *et al.* (*25*) (^14^C of correlated lacustrine ash). Volume % crystallinity (c) is from Geshi *et al.* (*23*).

	Iwato	Otsuka	Fukaminato (Lwr)	Fukaminato (Mid #1)	Fukaminato (Mid #2)	Arasaki-1	Arasaki-2	Arasaki-3	Kenashino (Lwr)	Kenashino (Upr)	Osumi (XR)	Osumi (XP)	Osumi (Gray)	Ito (XR)	Ito (Mod. xtal)	Ito (Gray)
Type	Pfl	Pfa	Pfa	Pfa	Pfa	Pfl	Pfl	Pfl	Pfa	Pfa	Pfa	Pfa	Pfa	Ignimbrite	Ignimbrite	Ignimbrite
Age (ka)	∼60 ka (a)	33.450 ± 0.346	32.249 ± 0.506	32.249 ± 0.506	32.249 ± 0.506	32.249 ± 0.506	32.249 ± 0.506	32.249 ± 0.506	31.11 ± 0.189	31.11 ± 0.189	30.009 ± 0.189 (b)	30.009 ± 0.189 (b)	30.009 ± 0.189 (b)	30.009 ± 0.189 (b)	30.009 ± 0.189 (b)	30.009 ± 0.189 (b)
Pumice samples	IW-01-01 to 14		F-A1-02, 03, 04, 05, 06, 07, 08	F-A1-01; F-A2-02, 03, 04, 06	F-A2-01, 05, 08	A-01, 02, 06-08, 12	A-03, 04	A-10, 13, 15	K-A-01, 02, 03, 05; K-B-05	K-A-04; K-B-01 to 04; K-B-06 to K-B-11	OS-B1-01,04, 05, 07, 10-13, 15; OS-B2-07;OS-B3-02, 04, 05, 09	OS-B2-03, 01, 02, 05, 06, 08-10, 12-15; OS-B1-02, 06, 08, 14	OS-B3-01, 06, 07, 13	ITO-D1-01	ITO-D1-02, 03-07, 09-15, ITO-D2-02, 03-08	ITO-D1-08, ITO-D2-01
Pumice selected	IW-01-04	OC4-02, 03, 06	F-A1-02	F-A2-02	F-A2-01	A-08	A-03	A-10		K-B-01	OS-B1-01	OS-B2-03	OS-B3-01	ITO-D1-01	ITO-D1-02	ITO-D2-01
Crystallinity (wt %)	37%	20–26%	4%	32%	31%	11%	33%	21%		11%	12%	2%	∼20–25%	18%	15%	11%
Crystallinity (vol %) (c)	22–29%	20%	18–20%	18–20%	18–20%	18–20%	18–20%	18–20%	18–20%	12–15%	12–23%	12–23%		12–23%	12–23%	12–23%
Petrography	Pl ≈ Qtz >> Opx (<1%) > rare oxides; Qtz is MI-rich	Pl > Qtz >> Opx > rare Cpx	Qtz > Pl >> oxides > rare Opx > rare Bt; Volcanic lithic fragments w/in pumice	Pl > Qtz >> Opx (1-2%) > rare oxides > rare Cpx	Pl >> Qtz >> Opx (∼3%), rare oxides; Pl often has blueish core	Pl > Qtz >> Opx; Dense glass. Poorly vesicular	Pl > Qtz >> Opx; Moderately vesicular	Pl > Qtz >> Opx; very Large quartz (some >2 mm); Highly vesicular		Pl >> Qtz > Opx (<1%) and Cpx (?), very rare small oxides; Abundant lithic inclusions	Pl > Qtz > Opx > oxides	Pl >> rare small Qtz > rare Opx > rare oxides; ∼Aphyric, elongated vesicles	Xtal-rich (<0.5 mm), Plag and Opx, No Qtz; Gray pumice, often mingled with white pumice glass	Large Pl > Qtz >> Opx > rare Hbl (?)	Pl >> small Qtz > rare Opx	Pl ≈ Qtz > Opx > Hbl (?); Gray pumice
Composition	High: Al_2_O_3_, Sc	Mod. high Sr, Zr, REE	High: Al_2_O_3_, Na_2_O, K_2_O	High: FeO, CaO, Na2O, Sr, Zr	High: SiO_2_, FeO, CaO, Sr		Similar to Fukaminato (lower)	High: Sc, most trace elements	High: SiO_2_, FeO, CaO, Sr, Y, La, Zr, Nd, Th	Similar to Fukaminato (mid #2)	High: FeO	High: Na_2_O, Li, Ba, Nd	High: CaO, FeO, Sr	High: SiO_2_, CaO	High: Li, Sr, Ba, Eu	High: SiO_2_
	Low: FeO, Sr, Y, Zr, Nb, Nb, Th		Low: FeO, CaO, Sc, Sr, Zr	Low: K_2_O	Low: Al_2_O_3_, K2O, Sc	Low: Ba, Y, Sr, Nb, La	Low: Ca,Sr		Low: Al_2_O_3_		Low: K_2_O, Sc, Nb	Low: V, Zr	Low: SiO_2_, Ba, Sc, Y, Nb, REE	Low: K_2_O, Sc, Ga, Ba, Nb, Pb		Low: FeO, Zn, Pb
MELTS *P* (avg)	104 MPa	93 MPa	114 MPa	110 MPa	89 MPa	101 MPa	108 MPa	89 MPa	90 MPa	86 MPa	112 MPa	131 MPa	Max = 222 MPa	89 MPa	120 MPa	93 MPa
MI H_2_O				4.1–4.7 wt %		4.5–7.2 wt %	3.5–6.1 wt %			3.8–6.3 wt %	3.5–5.5 wt %	5.8 wt %				
MI equil. pressure				105–135 MPa		135–175 MPa	104–215 MPa			100–215 MPa	80–180 MPa	249 MPa				
Mean pumice Zr	85 ppm	106 ppm	69 ppm	99 ppm	99 ppm	88 ppm	77 ppm	103 ppm	120 ppm	94 ppm	110 ppm	91 ppm	117 ppm	97 ppm	113 ppm	112 ppm
Mean zircon sat. temp.	742°C	759°C	728°C	752°C	753°C	743°C	733°C	756°C	769°C	749°C	761°C	745°C	769°C	752°C	764°C	764°C
Max. qtz residence	1600 years	310 years	590 years	420 years	1480 years	770 years	770 years	1180 years		740 years	620 years	60 years	No quartz	700 years	860 years	780 years

### Radiocarbon ages

We obtained the radiocarbon ages of paleosols underlying three pre-AT pumice fall deposits (Table 1, table S1, and data S7). The calibrated ages are 33,795 to 33,104, 32,755 to 31,743, and 31,298 to 30,921 calibrated years before the present (cal yr B.P.) for the paleosols below the Otsuka Pfa, Fukaminato Pfa, and Kenashino Pfa, respectively (all at 95.4% confidence level).

### Sample descriptions and petrography

Crystal contents range between 4 and 30%, by mass, for most pre-AT samples, although the pumice clasts from the Iwato ignimbrite contain up to 40% crystals (Table 1). Quartz and plagioclase are the most abundant phenocrysts; only the lower Fukaminato Pfa has a higher modal abundance of quartz than plagioclase. Most pre-AT pumice clasts also contain <3% pyroxenes. Fe-Ti oxide minerals tend to be small and rare but present in most samples. Lower Fukaminato Pfa also contains rare biotite and, along with the upper Kenashino Pfa, is unique in containing lithic fragments within the pumice clasts.

White pumice clasts from the AT eruptions tend to have lower crystal content than pre-AT pumice clasts, with 15 to 18% crystals in the Ito ignimbrite and 2 to 12% crystals (by mass) in the Osumi Pfa. These clasts contain, in decreasing abundance: plagioclase, quartz, orthopyroxene, and Fe-Ti oxides. In contrast, the gray pumice clasts from Osumi are more crystal-rich (∼20 to 25%), with abundant plagioclase and pyroxene phenocrysts and microphenocrysts, but no quartz. Some Osumi Pfa pumice clasts identified as gray in the field (sample names starting with “OS-B3”) are actually variably mingled with white glass with statistically similar compositions to the white Osumi Pfa pumice clasts and have therefore been grouped with the crystal-rich Osumi Pfa pumice (see Table 1).

### Pumice glass major and trace element compositions

**Fig. 2.**
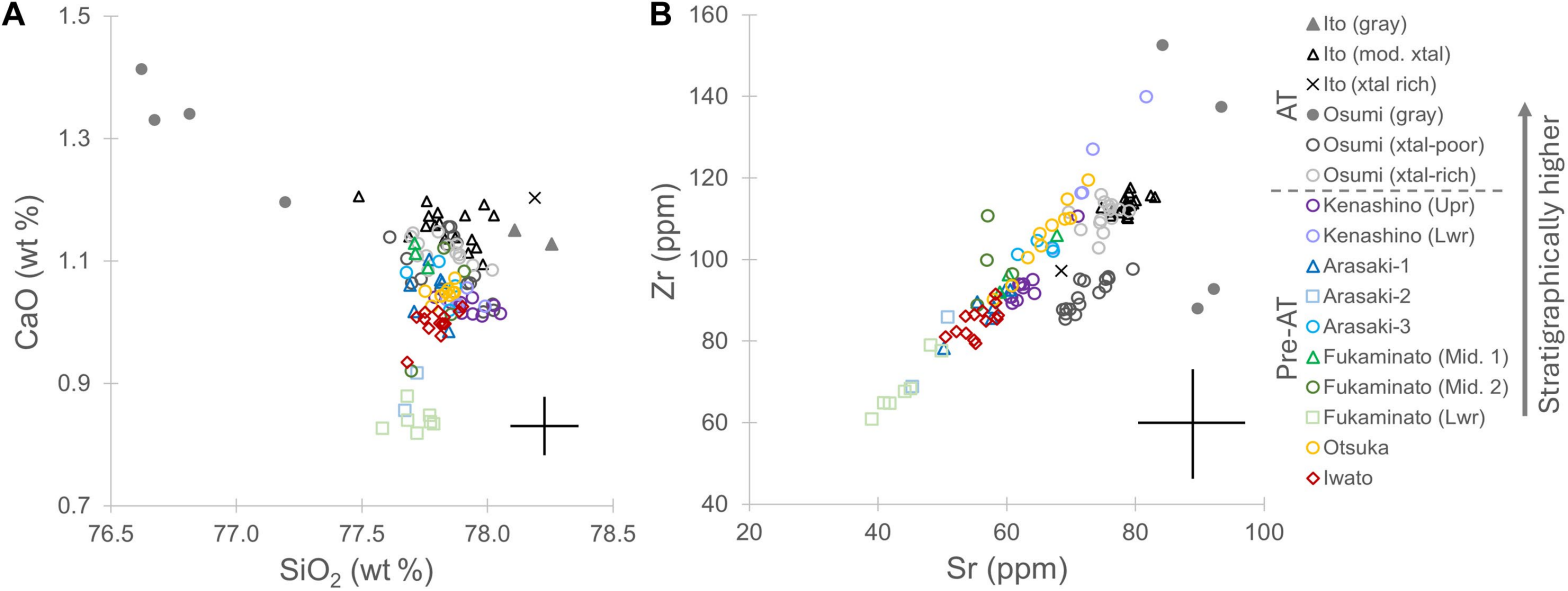
Bivariate plots of pumice glass major and trace elements. SiO_2_ versus CaO (wt %) is shown in (**A**), and Sr versus Zr (ppm) is shown in (**B**). Each point is the pumice clast average of 7 to 15 analyses. Color scheme is similar to the schematic stratigraphic section in Fig. 1, but with statistically distinct pumice populations shown as different shades and shapes (see statistical analysis within the Materials and Methods section for details). Error bars represent ±1σ analytical uncertainty, based on repeat analyses of the RGM-1 glass secondary standard. See fig. S1 for a comparison of glass to whole-rock data.

We collected pumice glass major element and trace element data using a scanning electron microscope with attached energy-dispersive spectrometer (SEM-EDS) and a laser ablation inductively coupled plasma mass spectrometer (LA-ICPMS), respectively (see the Materials and Methods section for details and Supplementary Files for all data). To investigate potential compositional heterogeneity, we collected in situ data from 12 to 15 pumice clasts from each subunit of the seven eruptive units in this study. All pumice clasts are high-silica rhyolites with average glass compositions that range between 77.5 and 78.3 wt % SiO_2_, with the exception of the gray pumice population from Osumi Pfa, which have averages between 76.5 and 77.2 wt % (Fig. 2 and fig. S1). All glass is subalkaline, with total alkali (Na_2_O + K_2_O) content of 7.0 to 7.5 wt %, although lower Fukaminato Pfa pumice clasts have slightly elevated values of 7.6 to 7.8 wt %. (Fig. 2). We used a Kruskal-Wallis nonparametric test to assess for statistical differences (*P* < 0.05) in the distributions of each major and trace element concentration between pre-AT, Ito-white, Ito-gray, Osumi-white, and Osumi-gray pumice. When compared to pre-AT pumice clasts, white pumice from Ito ignimbrite and Osumi Pfa have statistically significantly lower K_2_O, V, Ga, Nb, Ta, Th, and U and have higher CaO, Na_2_O, Li, Sr, Y, Zr, Ba, Hf, and rare earth element (REE) (Fig. 2). The gray pumice clasts from Osumi Pfa has significantly lower SiO_2_, Sc, Rb, Y, Nb, Cs, Ba, Th, U, and REE and have higher Al_2_O_3_, FeO, and Zn than all other pre-AT and AT pumice glass. Bivariate plots with glass data and whole-rock data from this study and the literature are shown in fig. S1.

Although all pumice glass has a high-silica rhyolite composition with a remarkably narrow range in SiO_2_, there are some within-unit and between-unit differences in major and trace elements that we identified using our multivariate statistical clustering approach (fig. S2). We find that Arasaki Pfl has three statistically distinct pumice populations (“Arasaki-1, Arasaki-2, and Arasaki-3”), and middle Fukaminato Pfa has two pumice populations (“Fukaminato middle-1 and Fukaminato middle-2”). Upper Kenashino, Arasaki-1, and Fukaminato middle-2 pumice are geochemically similar, whereas lower Kenashino, Arasaki-2 and Arasaki-3, Fukaminato middle-1, and Otsuka pumice are more compositionally similar. Iwato Pfl and lower Fukaminato Pfa tend to be compositionally distinct from these other pre-AT units, with generally higher Al_2_O_3_ and lower FeO, Sr, Y, and Zr. A multivariate analysis of variance (MANOVA) using less fluid-mobile elements (SiO_2_, Al_2_O_3_, FeO, CaO, Zr, Nb, Nd, and Th) reveals that AT glass compositions are statistically distinct in multivariate space from all pre-AT glass compositions (*P* < 0.05).

### Rhyolite-MELTS geobarometry

**Fig. 3.**
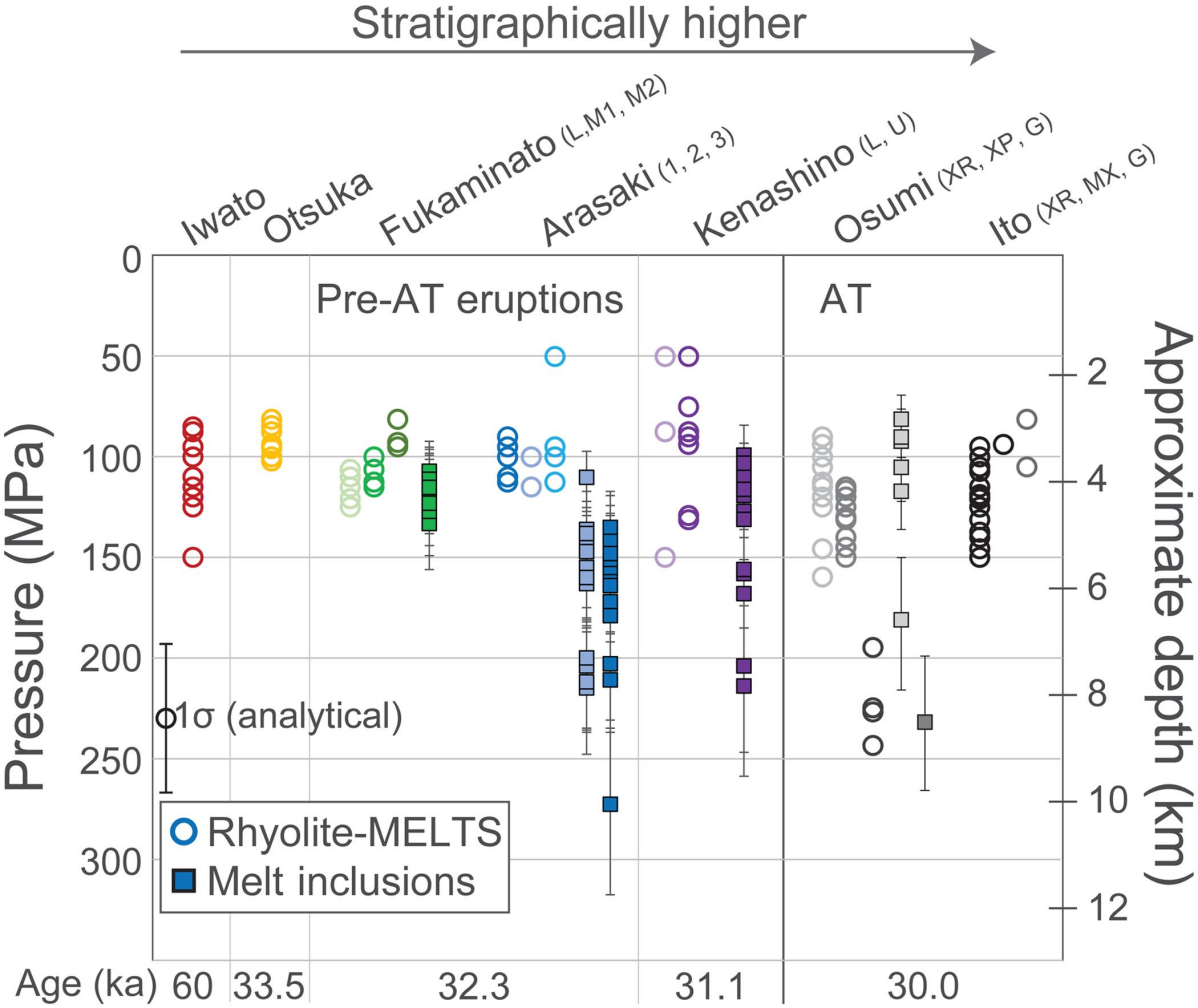
Storage pressures estimated by the rhyolite-MELTS geobarometer (circles) and by the H_2_O-CO_2_ content of quartz-hosted MIs (squares). Each circle represents the equilibration pressure calculated by rhyolite-MELTS using the average glass composition of one pumice clast, and different shades indicate statistically distinct compositional groups within some units. Note that, if the gray pumice from Osumi were not quartz saturated, the pressures are maxima. The ±1σ error bar on the left side shows the 38-MPa uncertainty in pressure due to analytical uncertainty alone, as calculated by Pitcher *et al.* (*13*) using the same instrument and procedure. Each square represents the saturation pressure of one MI, calculated using MagmaSat (*31*). Error bars are 1σ propagated from uncertainty in thickness and in the terms of the equations used to calculate H_2_O and CO_2_ contents from FTIR spectra. Approximate depth is shown on the right, assuming a crustal density of 2.7 g cm^−1^.

To estimate storage pressure of the Aira magmas, we first used the rhyolite-MELTS geobarometer (*30*), which determines the pressure at which a melt with the composition of each glass analysis was in equilibrium with the dominant mineral phases observed within each sample (i.e., plagioclase and quartz). The geobarometer successfully returned equilibrium pressures for 1246 of the 1489 individual glass compositions that were modeled (data S1). Because we find limited within-clast compositional variability for all pumice clasts, we suggest that, using the mean composition of each pumice clast likely yields a better approximation of the equilibrium pressure for each pumice clast than the range of individual analyses. The geobarometer returned pressures for 128 of the 131 clast-average compositions. Pressures calculated using average clast compositions range from 50 to 150 MPa for pre-AT, 80 to 160 MPa for AT magmas, and 195 to 245 MPa for Osumi gray pumice clasts (Fig. 3). However, because no quartz crystals were directly observed in the Osumi gray pumice, these represent maximum estimates if the magma was not saturated in quartz (*22*).

### H_2_O-CO_2_ geobarometry

In addition, we measured H_2_O and CO_2_ contents of quartz-hosted melt inclusions (MIs) by Fourier transform infrared (FTIR) to determine fluid-saturation pressures. MIs vary in appearance between different eruptions and even within a single eruption (Arasaki-1, Arasaki-2, and middle Fukaminato-1). Although most quartz hosts contain inclusions of various sizes and degrees of rounding, Arasaki Pfl inclusions tend to always be subangular (semifaceted). Quartz from Osumi Pfa, Kenashino Pfa, and Fukaminato Pfa have transparent glass MIs, whereas Arasaki Pfl has translucent brown glass. Although Osumi Pfa, Kenashino Pfa, and Fukaminato Pfa inclusions never contain vapor bubbles, Arasaki-1 (A-08) MI always contain a single large bubble (15 to 30 μm in diameter) whose volume is always less than 6% of the MI volume, and Arasaki-2 MI always contain numerous tiny bubbles (less than 5 μm in diameter). We observe no correlation between MI H_2_O and vapor bubble volume % in Arasaki, suggesting that the bubble has a negligible effect on MI H_2_O. Most inclusions from the crystal poor Osumi Pfa (OS-B2-03) were too small to analyze (<20 μm in diameter). All Ito ignimbrite quartz-hosted MIs contain large microlites of clinopyroxene, magnetite, sanidine, plagioclase, and quartz that range up to 40 μm in length. Because the crystallization of these MIs caused the glass to have artificially high volatile contents, we will not discuss the Ito ignimbrite MI FTIR data further.

The H_2_O and CO_2_ contents of MIs from most pre-AT and AT samples have significant overlap. The maximum H_2_O contents are 4.7, 7.2, 6.3, and 5.8 wt % for Fukaminato Pfa, Arasaki Pfl, Kenashino Pfa, and Osumi Pfa, respectively (data S2); minimum H_2_O contents are roughly similar, between 3.5 and 4.1 wt %. These values are consistent with those determined by Geshi *et al.* (*23*) using stoichiometric balance method with EDS analyses of quartz and orthopyroxene-hosted MIs, which range between 4 and 8 wt % (5th to 95th percentile). The maximum CO_2_ contents are 55, 141, 99, and 249 parts per million (ppm), respectively.

Saturation pressures estimated with MagmaSat (*31*) at 770°C range between 81 and 232 MPa for the Osumi Pfa and between 99 and 273 MPa for the pre-AT eruptions (Fig. 3). Pressures returned by MagmaSat change by less than 15 MPa when changing the temperature from 750° to 800°C, which are approximately the solidus and quartz saturation temperatures, respectively, as modeled with rhyolite-MELTS (*32*) using whole-rock initial compositions.

MagmaSat returned similar pressures to those estimated using the isobars for rhyolite calculated using VolatileCalc (*33*) (fig. S3). VolatileCalc always returns lower pressures; the difference in pressures is less than 30 MPa for all but the nine highest pressures, for which the difference ranges up to 68 MPa. We chose to use the MagmaSat pressures for this paper because they are thermodynamically constrained and are calibrated to the composition we input, rather than being a single extrapolated rhyolite composition. Furthermore, MagmaSat pressures are internally consistent with rhyolite-MELTS (*31*), which is used for rhyolite-MELTS geobarometry using glass compositions. Pressures estimated from volatile contents of MIs overlap with rhyolite-MELTS equilibrium pressures but range to 100 MPa higher values (Fig. 3).

### Geothermometry

Pumice clast average glass Zr contents are primarily between 70 and 120 ppm for pre-AT clasts and between 85 and 120 ppm in AT clasts. Compositional *M* values (*34*) are nearly all identical, between 1.24 and 1.35. Zircon was not directly observed, but average glass Zr is 8 to 60 ppm lower than whole-rock values from the same pumice clast, consistent with zircon saturation.

Pre-AT pumice clast average zircon saturation temperatures using the geothermometer of Watson and Harrison (*34*) primarily range (5th to 95th percentile) between 721° and 771°C, although pumice clasts from the lower Fukaminato Pfa have distinctly lower temperatures (714° to 734°C) and some lower Kenashino Pfa pumice clasts have higher temperatures (757° to 782°C) (fig. S4). Although Ito ignimbrite clusters tightly at 761° to 766°C and most of Osumi Pfa pumice clasts have a similar temperature range (739° to 763°C) to the pre-AT samples, the gray pumice clasts from Osumi Pfa range to higher temperatures (740° to 800°C).

The zircon saturation thermometers of Boehnke *et al.* (*35*) and Gervasoni *et al.* (*36*) give temperatures that are 45° to 55°C and 16° to 81°C lower than the Watson and Harrison (*34*) thermometer, respectively. We prefer the thermometer of Watson and Harrison (*34*) because almost all temperatures estimated by the other two thermometers are below 720°C, which is below the solidus temperature that we calculated for most samples using rhyolite-MELTS. Zircon saturation temperatures estimated by all three thermometers are given in data S3.

### Quartz diffusion timescales

**Fig. 4.**
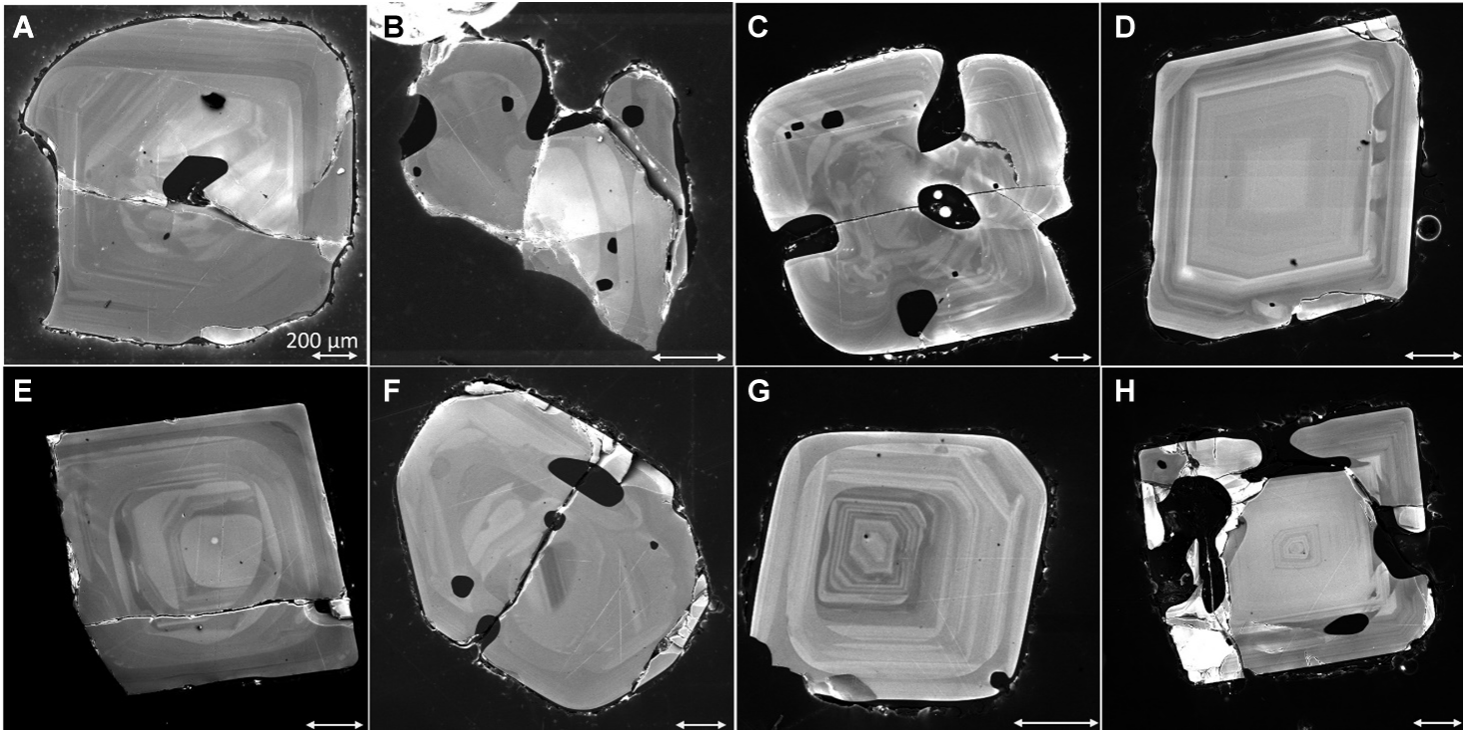
CL images of quartz crystals with variable growth/dissolution textures. Brighter regions have higher Ti content. (**A**) Osumi (crystal-rich) quartz has partially dissolved rim (truncated zones). (**B**) Osumi (crystal-poor) quartz has highly dissolved/embayed rim. (**C**) Arasaki-3 quartz has highly resorbed rim and has a much higher zircon saturation temperatures than lower Fukaminato (**D**), which tend to have euhedral rims and interior. (**E** and **F**) Clearly resorbed cores from Iwato and Kenashino quartz grains. The Fukaminato quartz in (**G**) shows nearly constant oscillatory zoning. (**H**) Skeletal growth in quartz from lower Fukaminato. White arrows in the bottom right of all images are 200 μm.

**Fig. 5.**
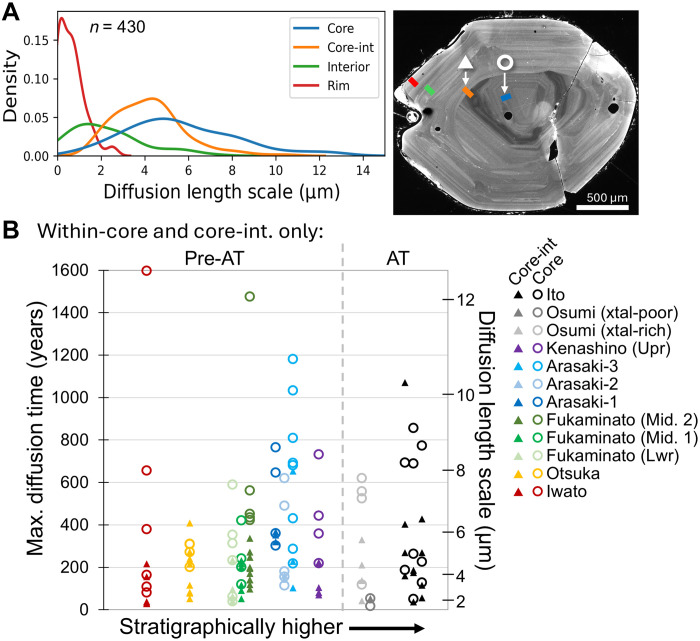
Quartz diffusion chronometry results. (**A**) Kernel density plot of modeled diffusion length scales for different regions of quartz from all samples. The CL image of a quartz (A-10_02) on the right shows an example of these regions, where the color of the profile corresponds to the kernel density lines on the left. “Cores” are defined as profiles that are coreward of the first visible zonation truncation [circle in (B)]. “Core-int” profiles cross the first visible zonation truncation and thus represent diffusion time since the first resorption event [triangles in (B)]. Note that the modeled diffusion length scales increase toward the core, consistent with longer diffusion times. (**B**) Diffusion timescales for the cores (circles) and core-interior boundary (triangles) of quartz crystals using the Ti diffusivity of Cherniak *et al.* (*18*) at 750°C. Corresponding diffusion length scales are shown on the axis on the right. Regardless of the diffusion coefficient, there is no consistent increase in diffusion length scale up-section. Note that Fukaminato Pfa (green) and Arasaki Pfl (blue) were co-erupted.

We collected cathodoluminescence (CL) images for 20 to 25 quartz crystals from a representative pumice clast from each statistically distinct pumice population. Quartz crystals from all units display a wide range of interior zoning as seen in CL images, from simple oscillatory to more complex zoning patterns, often with at least one resorption event seen as truncation of zones (Fig. 4). The correlation of CL grayscale with Ti in quartz has been demonstrated by numerous studies (*9*, *11*, *37*, *38*). We modeled one-dimensional (1D) diffusion of Ti in quartz using CL grayscale values of profiles taken across major zoning boundaries and fitting a complementary error function (fig. S5) (*11*). Diffusion profiles across the core-most zoning boundary have maximum characteristic length scales (*L*) that range between 2.7 and 18.0 μm. Although core timescales are the focus of this present study, modeled *L* values of quartz interior zoning boundaries decrease progressively rimward in almost all quartz crystals, consistent with diffusion being the driver for the observed CL brightness profiles (Fig. 5A) (*11*, *39*).

Diffusion timescales are vastly different depending on which Ti-in-quartz diffusion coefficient is used. Using Cherniak *et al.* (*18*) diffusivity (at 750°C) gives the shortest timescales, typically ranging between 45 and 1070 years (5th to 95th percentile), with several values that range up to 1600 years (Fig. 5). Timescales are much longer using the diffusion coefficients from three recent publications. Henceforward, we will use the abbreviations C07, A23, A21, and J20 to represent the timescales determined using the coefficients from Cherniak *et al.* (*18*), Audétat *et al.* (*21*), Audétat *et al.* (*19*), and Jollands *et al.* (*20*), respectively. Using A23, core timescales range from 17 to 410 kyr (5th to 95th percentile), J20 gives 54 kyr to 1.3 Myr, and A21 gives the longest timescales of 175 kyr to 4.2 Myr. As a result, timescale estimates for the same diffusion profile differ by a factor of up to 4000, depending on the diffusion coefficient used (data S4). We discuss these differences in depth in the Discussion section.

There are some differences in the range of diffusion timescales between units. Although all the pre-AT units and Ito ignimbrite have roughly similar ranges of 25 to 1600 years and 50 to 1070 years, respectively using C07, quartz from the crystal-rich pumice (OS-B1-01) of the Osumi Pfa yields shorter timescales (120-620 years); the crystal-poor pumice (OS-B2-03) yields even shorter core diffusion timescales, ranging between 7 and 120 years (Fig. 5). It should be noted that crystal-poor pumice from Osumi Pfa contains much smaller crystals and we could only find true cores within two crystals, which were both less than 0.7 mm in size compared to crystals from crystal rich Osumi pumice that range up to 2 mm (fig. S6). Short timescales in OS-B2-03 could either be the result of not sampling larger crystals or could truly indicate that the magma spent less time crystallizing, leading to smaller crystals.

### Plagioclase textures and compositions

Most plagioclase crystals in all units have a patchy core, consisting of low-An patches surrounded by a euhedral envelope of high An, followed by a sharp decrease in An, and then relatively constant composition or low-amplitude oscillatory zoning to the rim (fig. S7). Some crystals have one or two zoning truncations and/or sawtooth textured boundaries, likely indicating crystal resorption. Patchy cores sometimes contain small mineral or MIs, but such inclusions are never found outside of the core. Rims tend to be euhedral to slightly rounded.

The low-An patches within cores of plagioclase from all samples range between An37 to An55, whereas the high-An envelope that surrounds them ranges between An60 to An93 and has lower Ba, Li, Pb, and Mg, as expected from normal partitioning (fig. S8). However, for most crystals, the Sr content is roughly similar between the low-An and high-An patchy zones. Although texturally similar, there are some statistical differences between high-An cores of plagioclase from pre-AT and AT units, using a Kruskal-Wallis test (*P* < 0.05). Ito ignimbrite and Osumi Pfa both have statistically significantly higher-core An, with Ito ignimbrite being higher than Osumi Pfa. Ito ignimbrite has significantly higher An, Li, Ti, Sr/Ba, and Li/An and lower Mg, Ba, Fe/An, Mg/An, Ba/An, La/An, and Pb/An than pre-AT cores (data S1). Notably, Ito ignimbrite plagioclase has higher Li for a given An content than Osumi Pfa or any of the pre-AT plagioclase. Although gray pumice from Osumi Pfa has distinctly different glass major and trace element compositions (e.g., lower SiO_2_) compared to white pumice from the same eruption, plagioclase core compositions are remarkably similar between the two pumice populations.

Rimward of the major core-interior boundary, most plagioclase grains have minor variation in An content over the 200- to 500-μm distance to the crystal edge. AT and pre-AT plagioclase crystals have similar An content in these outer regions, with a median composition of An38-An42 for all samples and 90% of analyses ranging between An30 and An50. The interquartile range (IQR) of the interior and rim analyses from all samples is less than 7 mol % An (that is, 50% of analyses have a narrow range of only 7 mol %). However, rim compositions of AT plagioclase have higher Sr and lower Ba and Pb compared to pre-AT samples. Unlike the similarities in core compositions, gray pumice from Osumi Pfa has higher Fe and lower Li, Ba, and light rare earth element (LREE) than white pumice from Osumi Pfa and all other samples.

## DISCUSSION

### Diffusion timescales

Our primary goal is to determine whether the long-term thermochemical state of the magmatic system is one that supports eruptible or uneruptible magma. Was the magma that ultimately fed the Aira CFE in an eruptible state during the three preceding eruptions or only for ∼1 kyr (after the final pre-AT eruption)? Answering this question is not only important for the Aira caldera system itself but also for our understanding of caldera systems worldwide; are these large volumes of silicic magma commonly kept at high enough temperatures to be eruptible (<50% crystals) (*7*) for long periods of time (“warm storage”), or are they kept primarily as a rheologically locked-up crystal-rich mush, punctuated with short periods of high temperature defrosting events that create short-lived melt-rich bodies (“cold storage”)?

By strategically choosing to model diffusion using the temperature of rheological lock-up (∼50 to 60% crystals) (*7*), the resulting timescales give the maximum amount of time that the magma spent at or above this temperature and thus in an eruptible state. Modeling the evolution of Aira magmas with the whole-rock compositions in this study using rhyolite-MELTS demonstrates that all compositions reach 60% crystals (rheological lock-up) between 742° and 750°C at 200 MPa (fig. S9), the maximum pressure calculated with the rhyolite-MELTS geobarometer. Therefore, we chose to model diffusion at 750°C to provide maximum time spent above rheological lock-up. It should be noted that inherent averaging due to beam interaction volume, modeling 1D diffusion, and assuming an initial step function all cause these calculated timescales above 750°C to be maxima (*38*).

We modeled diffusion within the cores of quartz grains and estimate the total residence time of AT quartz at or above 750°C (within melt-dominated magma) to be less than 1100 years (using C07) (Fig. 5). Because the AT eruption temperature, determined by Fe-Ti oxides (*23*, *40*), is between 780° and 800°C, the true residence time in an eruptible state must be much shorter. The magma could have spent no more than 240 years at 800°C, which is the highest temperature possible without dissolving quartz (if stored at 125 MPa) (fig. S9).

**Fig. 6.**
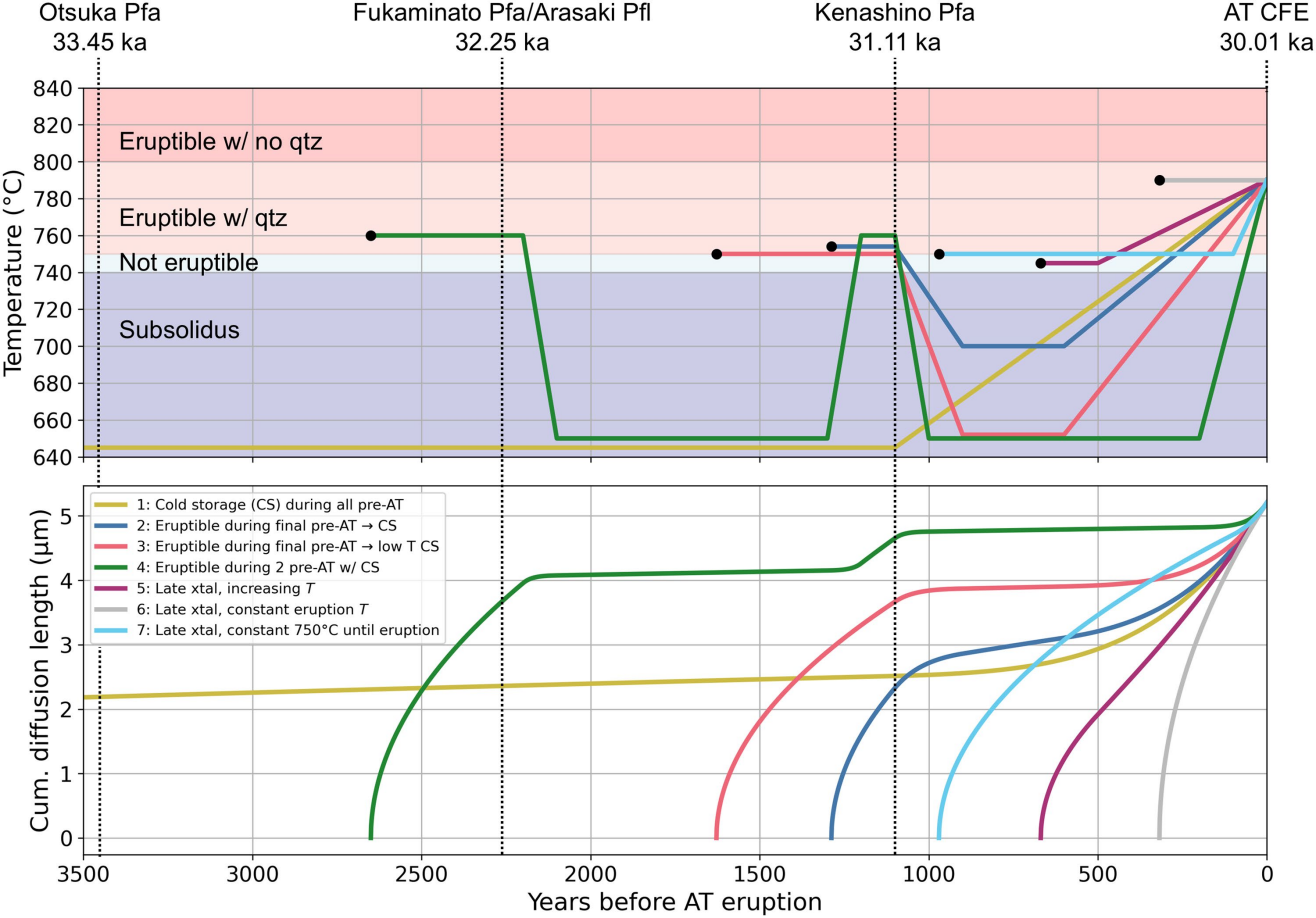
Potential thermal histories of AT magma determined by inverse modeling. For each potential temperature-time pathway, the maximum observed Ti diffusion distance in AT quartz (*L* = 5.21 μm, one side of the complimentary error function) is achieved (using C07). Top figure shows seven examples of plausible temperature-time pathways (legend is in the lower plot). The eruption temperature of 790°C is inferred from Fe-Ti oxide geothermometry (*23*). Black dots represent the onset of quartz crystallization. The crystal in path 1 begins crystallizing 10,500 years before AT eruption and rapidly cools to subsolidus conditions at 650°C (not visible on the reduced *x* axis). Although the maximum time in an eruptible state (above 750°C) is 1070 years, cold storage extends the total diffusion time (e.g., paths 1 to 4), and storage at hotter temperatures reduces the total residence time (e.g., paths 5 to 7). The bottom plot shows the cumulative diffusion length for each of the *T*-*t* paths above; all converge on the maximum diffusion length observed in AT quartz. Note that timescales of eruptibility are brief, always less than the repose time leading up to the AT eruption.

Considering that even the 750°C timescale is shorter than the time between the pre-AT and AT eruptions, this implies that the AT magma could not have been held in a melt-rich state (“warm storage”) for the entire duration of repose time between the eruptions. There are infinite temperature-time (*T*-*t*) paths that could cause diffusion in quartz to reach the observed Ti diffusion length scale. We used inverse modeling to simulate some of the possible magma *T*-*t* histories, each leading to the maximum observed Ti diffusion length scale of 5.21 μm (one-sided) (Fig. 6). There are three categories of plausible magma histories. AT magma could have begun crystallizing quartz thousands of years before the AT eruption but were then frozen in a subsolidus pluton during all of the pre-AT eruptions, only to be defrosted after the final pre-AT eruption (path 1 in Fig. 6). Equally valid are *T*-*t* histories in which the AT magma had an eruptible viscosity during at least one of the pre-AT eruptions but cooled below 750°C for some time before being defrosted before the AT eruption (paths 2 to 4 in Fig. 6). It should be noted, however, that, with each additional heating and cooling cycle modeled, it requires steeper temperature changes and less time in an eruptible state to fit the observed profile (e.g., Scenario-4). Last, AT quartz could have begun crystalizing only after the final pre-AT eruption and the AT magma was eruptible (750° to 800°C) for much less than 1000 years (paths 5 to 7). Regardless of the scenario, diffusion timescales (using C07) indicate that the long-term thermal state of the Aira magma reservoir must be “cold storage” in a crystal-rich mush or pluton with ephemeral windows with eruptible viscosity. Similarly, quartz from pre-AT eruptions have maximum timescales of eruptibility that do not overlap with preceding eruptions.

However, as we discuss in the next section, there is currently major uncertainty with these timescale estimates because conflicting diffusion coefficients proposed in recent years produce timescales that span over three orders of magnitude. Thus, we also model plagioclase diffusion as an independent timescale estimate.

**Fig. 7.**
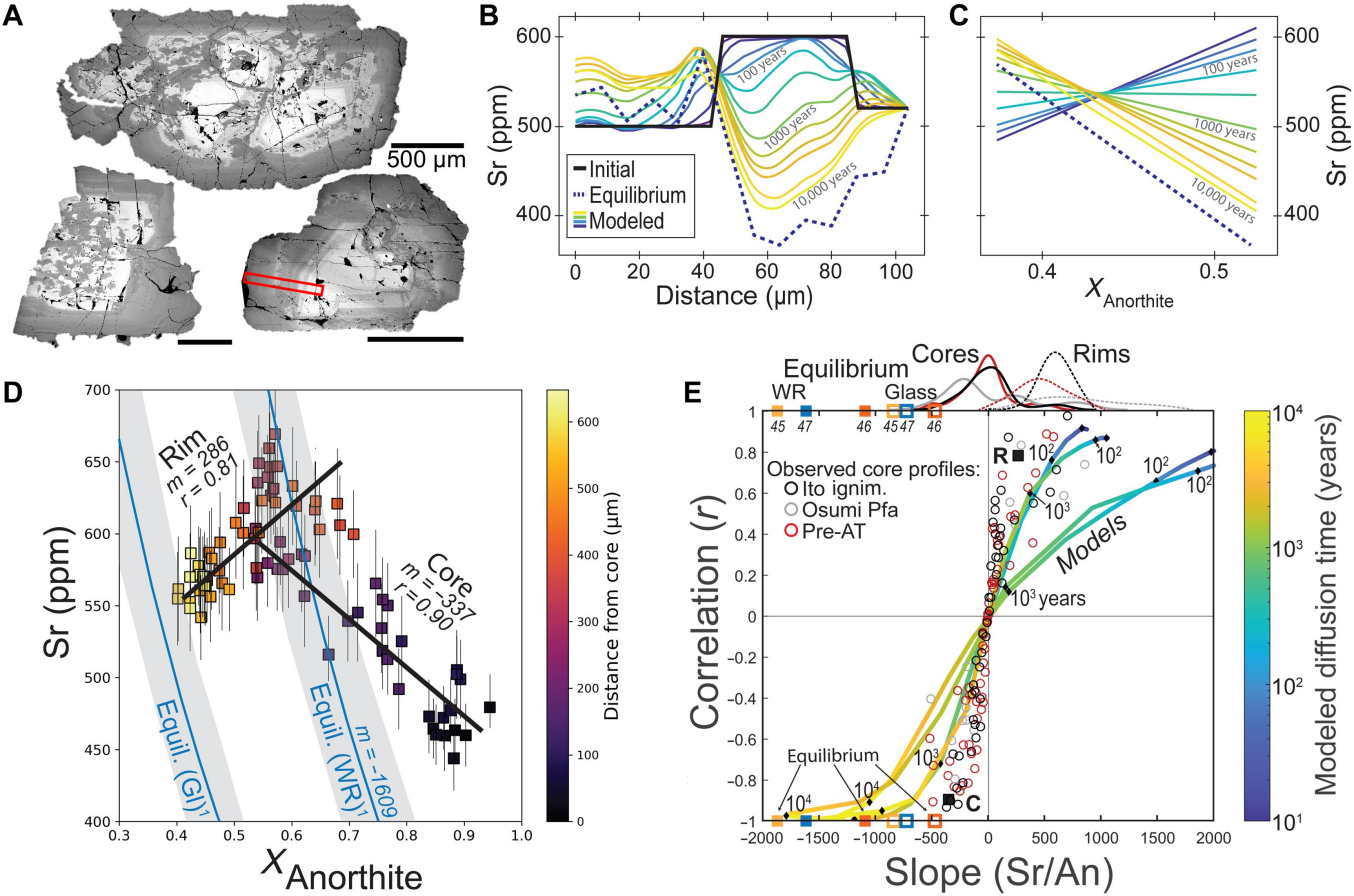
Plagioclase textures, Sr diffusion forward modeling approach, and results. (**A**) BSE images show common plagioclase textures (ITO-D1-01_Plag-8, F-A2-02_Plag-4, and ITO-D1-02_Plag-12, respectively). Scale bars, 500 μm. (**B** and **C**) An example of one forward model shows changes in the Sr profile and Sr versus An from initial conditions (black line) toward equilibrium (blue dashed line). The color scale is shown in (E). (**D**) Example of observed Sr and An data from ITO-D1-02_Plag-12 (red box in A), colored by distance from the core-most analysis. Blue curves show the plagioclase compositions (±2σ) expected if full diffusive equilibrium were reached with a magma with 80 ppm Sr (average glass Sr) or 180 ppm Sr (average whole-rock Sr). Solid black lines are linear regressions for the core and rim, with slope (*m*) and correlation (*r* value) shown. (**E**) Observed slope and *r* values of all plagioclase core segments (*N* = 120). The core and rim in (D) are plotted as black squares labeled with “C” and “R,” respectively. Kernel density plots of the slopes of all core (solid lines) and rim segments (dashed lines), are plotted at the top, as well as the slopes expected if equilibrium were to be reached with a melt with 80 ppm Sr (filled squares) and 180 ppm Sr (open squares), using the three partitioning models [Nielsen *et al.* (*47*), Bindeman *et al.* (*45*), and Dohmen and Blundy, (*46*)]. The colored curves on the figure show the modeled progression of the Sr/An slope and *r* value of four forward diffusion models (color scale on the right). Black diamonds are labeled with the diffusion time (in years) needed to reach that slope and *r* value. Only models using the partitioning equation from Nielsen *et al.* (*47*) are shown here for clarity. See fig. S10 for comparison to all equilibrium slopes using all three partitioning models.

Many studies have used a finite difference methodology to model Mg, Ba, or Sr diffusion within plagioclase to determine timescales (*41*–*44*). However, for most plagioclase grains in this study, this approach could not be confidently used; we discuss the complications that prevent standard approaches for modeling diffusion of these elements in the Materials and Methods section. Instead, we establish maximum timescales of Sr diffusion using a forward modeling approach, adapted from Cooper and Kent (*14*) and explained in more detail in the Materials and Methods section. We take advantage of the fact that plagioclase crystallizing in a fractionating melt (with progressively decreasing melt Sr and Ca) will initially have a strongly positive slope of Sr versus An; however, diffusive reequilibration will cause the slope and the correlation of Sr versus An to move toward a progressively stronger negative trend until reaching equilibrium (Fig. 7, B and C). We compare the observed Sr/An slope of Aira plagioclase cores and rims to the calculated slope of the equilibrium Sr versus *X*_An_ using the partition coefficient equations from three different sources (*45*–*47*); the slope of this line is what we would expect if the Sr had reached diffusive equilibrium within a plagioclase crystal. Because we can expect the Aira melt to have had Sr contents between that of the whole pumice (180 ppm) and the glass (80 ppm), the equilibrium Sr (ppm)/*X*_An_ slopes should be ∼−715 or −1609, respectively, according to the partitioning equation of Nielsen *et al.* (*47*). Using all three relevant published partitioning equations gives equilibrium slopes that range between −479 and −1865 (fig. S10). None of the observed plagioclase core data reach such negative slopes, indicating that they have not reached diffusive equilibrium. An example is shown in Fig. 7D. After calculating the slope and *r* value for 120 core transects and 46 rim transects, we observe that plagioclase rims tend to have much more positive slopes of Sr/An than cores, consistent with cores having experienced more diffusion (kernel density curves; Fig. 7E). Aira plagioclase cores tend to have Sr/An slopes that are less negative than −500 and *r* values that tend to be less negative than −0.80 (Fig. 7E). This is far from the strong negative trends, with slopes of up to −829 and −1865, that would be expected from equilibrium with the glass and whole-rock Sr contents (80 and 180 ppm), respectively (colored boxes; Fig. 7E). We can therefore be confident that Sr has not fully reequilibrated within the plagioclase cores in this study. Our forward models reach a slope of −500 after 1200 to 2500 years, depending on the initial conditions, suggesting that, even if the plagioclase cores started with strong positively correlated initial profiles, Sr could have only been diffusing for a maximum of 2500 years at 750°C to reach the most negative observed slope. The diffusion time at 800°C would only be 800 years. These maximum timescales of less than 2500 years determined by our forward modeling approach are consistent with the timescales for three cores (390 to 2370 years) that we could confidently model using the finite difference methodology of Schleider *et al.* (*41*) and Lubbers *et al.* (*43*). Using the partition coefficients of Dohmen and Blundy (*46*) gives slightly longer timescales of up to 4500 years (see the Materials and Methods section for details). We discuss the implications of a new Sr diffusion coefficient (*48*) later.

Plagioclase diffusion timescales are therefore of similar magnitude to quartz diffusion timescales that tend to be less than 1100 years using C07. In contrast, the plagioclase diffusion timescales are significantly shorter than timescales of 400 kyr to 4 Myr using the three newer Ti-in-quartz diffusion coefficients. We discuss this further below.

### Possible scenarios for the Aira magmatic system

In this section, we discuss possible petrogenetic scenarios for the pre-AT and AT magmas. Considering that all pre-AT and AT pyroclastic eruptions have depositional patterns that show an eruption source within the current Aira caldera (*23*, *27*), we have three possible scenarios. In Scenario-1, the AT magmas were stored at different depths and were distinct from pre-AT magmas. Because these magmas were disconnected in this scenario, the AT magma may or may not have been in a melt-rich state during the prior eruptions. In Scenario-2, pre-AT and AT magmas were sourced from the same approximate storage region and were at eruptible viscosities for much longer periods than the repose time between eruptions (i.e., >1 kyr). This scenario requires pre-caldera eruptions to selectively tap only a portion of the >400-km^3^ eruptible magma, and the rest is erupted during the AT caldera-forming event. In Scenario-3, pre-AT and AT magmas were also sourced from the same storage region, but the AT magma was uneruptible during prior eruptions and the 400-km^3^ melt-rich magma was rejuvenated from a crystal-rich mush within the 1 kyr between the Kenashino Pfa and AT eruptions.

### Comparing pre-AT versus AT magma storage

In order for Scenario-1 to be correct, we would expect that the unrelated pre-AT and AT magmas should have both distinct compositions and different storage depths. Although all pumice clasts, with the exception of the gray pumice from Osumi Pfa, have a remarkably narrow range in SiO_2_ (77.5 and 78.3 wt %), there are statistical differences between pre-AT and AT pumice in some major and trace elements. For example, although pre-AT pumice clasts tend to have similar compositions or lie along a single bivariate trendline, AT pumice glass tends to be displaced to higher CaO and Sr, which may indicate the AT magmas fractionated less plagioclase (Fig. 2). However, it is unlikely that these compositional differences are due to degree of fractionation alone because despite containing significantly less crystals (<1 wt % versus 12 wt %), the crystal-poor Osumi pumice has similar glass CaO and Sr to the crystal-rich Osumi pumice (Fig. 2). In addition, the lower Fukaminato pumice contains less crystals (4 wt %) than pumice clasts from upper Fukaminato Pfa or any other pre-AT unit, yet it has distinctly lower Sr and CaO than any other unit, the opposite of what would be expected if fractionation of a single magma were responsible for the trends in major and trace elements. Alternatively, the AT magmas could have been extracted from a different mush (or a different region of the same mush body) that had experienced more plagioclase melting leading to a higher initial Sr content, although whole-rock data from this study and from Geshi *et al.* (*23*) and Kuritani (*49*) show significant overlap and, in some cases, even lower CaO and Sr than pre-AT samples (fig. S1). Furthermore, Kuritani (*49*) demonstrate that, with the exception of the oldest pre-AT (Iwato Pfl) eruption, the rhyolite pumice from all eruptions has similar Sr and Nd isotopic compositions, suggesting a similar origin. Therefore, these compositional differences alone are not enough evidence to conclude that there was no cogenetic relationship between the magmas, whether from the same eruptible melt or extracted from the same mush (*50*, *51*).

Magma storage depths determined the rhyolite-MELTS geobarometer are similar between pre-AT magmas (50 to 150 MPa) and those of the AT eruption (80 to 160 MPa), with the exception of the gray Osumi pumice (Fig. 3). Saturation pressures estimated with MagmaSat (*31*) using H_2_O-CO_2_ concentrations in quartz-hosted MIs give a range of pressures, mostly between 80 and 250 MPa, with significant overlap between all eruptions. Despite this range, all crystals in all samples have a clear minimum water content that equates to pressures of 80 to 135 MPa, which is identical to the range of rhyolite-MELTS equilibrium pressures for most pre-AT and AT magmas.

Water contents of MIs and associated saturation pressures vary widely between MIs from a single pumice clast or even within a single host quartz. For example, although 10 MIs from just two crystals from Fukaminato Pfa have nearly identical saturation pressures (107 to 133 MPa), we observe a 40- to 200-MPa range within single quartz crystals (fig. S11) from the pyroclastic flow unit of the same eruption (Arasaki Pfl). Such within-crystal variability could be the result of MIs being entrapped at different depths during polybaric crystallization. Alternatively, it could be the result of the host magma ascending to a shallower staging region and stalling long enough to allow for differing degrees of diffusive water loss from MIs within a single crystal (due to MI size or distance) but not long enough to allow all MIs within each quartz to reequilibrate to the shallower depth (*52*). In this case, the narrow range of MI H_2_O content in lower Fukaminato (4.1 to 4.7 wt % H_2_O) would be the result of these crystals having spent more time at this shallow level, allowing for the reequilibration of all MIs. We propose that the shallow Aira magma storage system may span a range of 200 MPa within the upper crust, such that magmas of both the pre-AT and AT eruptions ascended from a deeper 200- to 280-MPa storage region (∼7 to 10 km) and temporarily stalled within a shallower storage zone of 80 to 140 MPa (∼3 to 6 km), consistent with the rhyolite-MELTS geobarometer. Modeling magma ascent history is beyond the scope of this paper and will be our focus in a future study. However, we can be confident that all eruptions tapped magmas that were stored in a complex storage system with a similar shallow final storage depth.

This complex storage system also allows for different magma populations to be co-erupted, such as the Fukaminato Pfa and Arasaki Pfl, which each have three statistically distinct pumice glass compositions (Fig. 2 and fig. S2) and which have differing ranges in volatiles within quartz-hosted MI (Fig. 3 and fig. S11). In addition, each pre-AT eruption has pumice populations that are statistically similar to compositions in other pre-AT eruptions (Fig. 2 and fig. S2). In summary, there is evidence for compositionally different magma batches within individual eruptions, each stored at slightly distinct levels and with slightly different ascent histories. Similar evidence of co-eruption of distinct magma batches have been found at many other caldera systems worldwide (*3*, *5*, *13*, *53*, *54*).

These pressure ranges overlap both with the results of Geshi *et al.* (*23*) who used H_2_O-only in quartz-hosted MIs to propose AT storage at 140 to 260 MPa, and the findings of Yasuda *et al.* (*55*) who used orthopyroxene-melt and amphibole geobarometers to propose AT storage near of 80 to 110 MPa. Our storage pressure range, which equates to depths of ∼2 to 11 km is consistent with the results of Miyamachi *et al.* (*56*), who argue that the solidified remnants of the AT magma reservoir was between 4 and 11 km, based on a substantial high velocity zone detected by active source seismic tomography. Thus, our results suggest that, although the AT magma system may have extended to slightly higher pressures, there was significant overlap in the storage depths of the pre-AT and AT magmas.

Although we see some differences in the AT units compared to the pre-AT, such as slightly deeper rhyolite-MELTS equilibration pressures and some major and trace element differences (e.g., higher Sr and CaO), there is not enough evidence that these magmas were completely unrelated, and there is too much overlap in both equilibration pressures and MI volatile saturation pressures to infer different storage depths for AT and pre-AT magmas. Thus, we suggest that Scenario-1, that the magmas were unrelated and stored in separate disconnected storage zones, is not consistent with our results.

### Evidence for short eruptible timescales

**Fig. 8.**
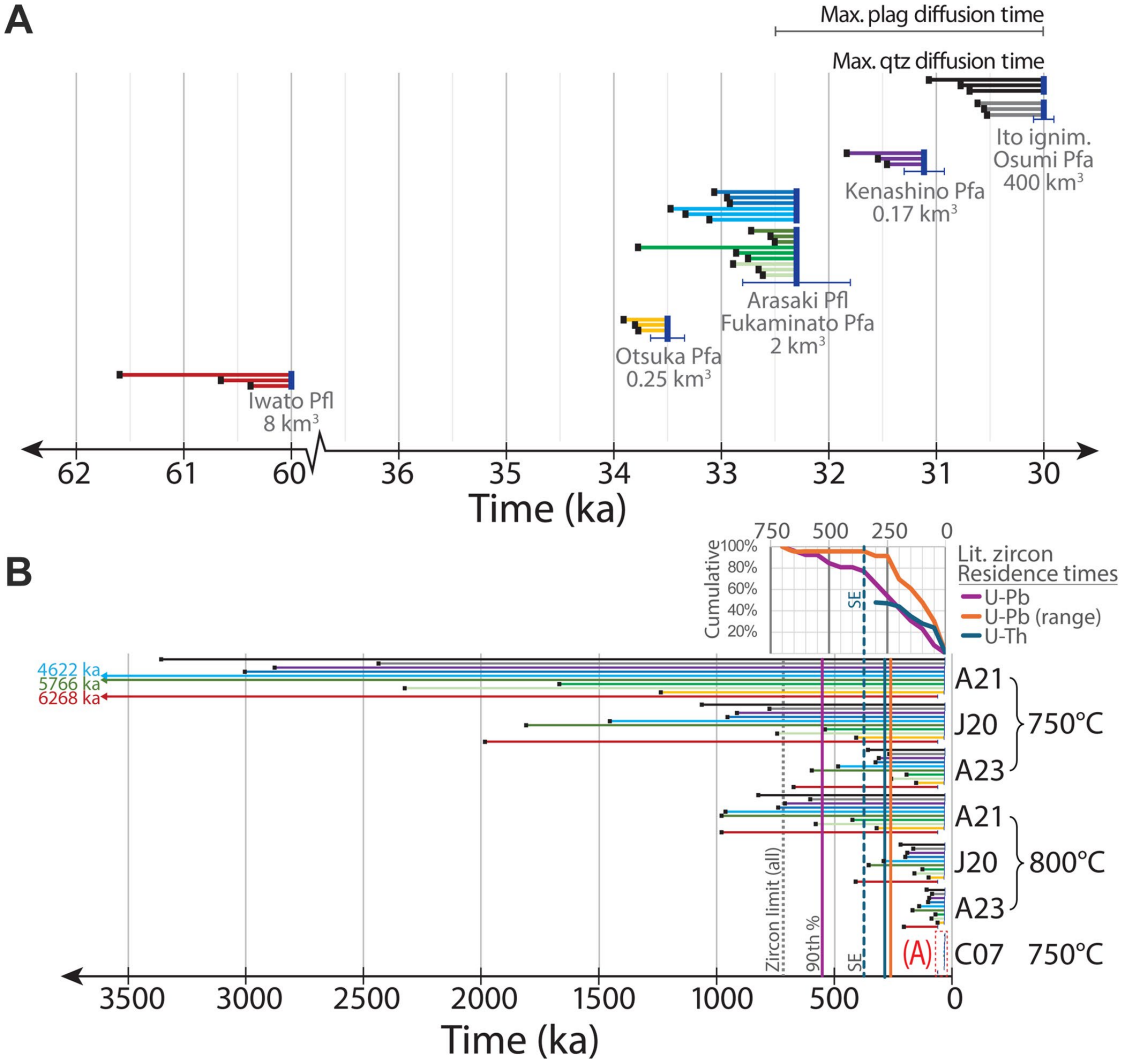
Timelines relating diffusion timescales to eruption ages. (**A**) Timeline showing the three longest maximum quartz residence times (above 750°C) of each pumice population, using C07. Timescales project from the ^14^C eruption ages, which are plotted in dark blue (±2σ). The range of maximum plagioclase Sr diffusion times (1200 to 2500 years) determined by this study are comparable to quartz residence times and are shown above the AT timescales for comparison. (**B**) Timeline with the maximum quartz diffusion timescale from each pumice population, calculated using A21, J20, and A23 at 750° and 800°C. Note the change in timescale axis; the scale of (A) is shown by the red box at the bottom of (B). The curves in the figure at the top of (B) show the cumulative frequency of literature zircon residence times, determined by U-Pb dating (oldest zircon age minus the eruption age), U-Pb range (oldest age minus youngest age), and U-Th disequilibria dating (minus eruption age) (*97*). To compare zircon residence times from other systems to Aira quartz residence, cumulative density function (CDF) curves are set to have the same 30-ka eruption age as the AT eruption. The 90th percentile of each zircon residence time is projected down onto the timeline for comparison. “SE” indicates that no U-Th residence times larger than 350 kyr are resolvable due to secular equilibrium. Data compilation and sources are given in data S6.

Considering that we have demonstrated that the magma erupted during the AT eruptions was sourced from a similar storage region as pre-AT eruptions, we next discuss the longevity of eruptible magma within the Aira system. This is complicated, however, by the choice of *D*_Ti_ that is used. We estimate quartz residence times in eruptible magma (>750°C) of less than 1 kyr (95th percentile), using C07, or much longer residence times of 410 kyr (A23), 1.3 Myr (J20), or 4.2 Myr (A21) (Fig. 8).

Because of this ambiguity, we turn to our plagioclase data and its petrogenic relationship with quartz. Our forward modeling results show that both AT and pre-AT plagioclase crystals (*n* = 120 profiles) have Sr versus An trends of their core-mantle boundaries that indicate they could have only resided above 750°C for less than 1200 to 2500 years. It should be noted that, because these are transects across the core-mantle boundary, not entirely within the core, these represent the diffusion timescales after the mantle began to grow onto the cores of the crystals. The commonly observed high-An (An80-90) patchy texture likely indicates that the cores of many plagioclase crystals are antecrysts that were partially melted and remobilized (fig. S7). Our modeled diffusion time is therefore likely the time spent above 750°C because the antecrystic core experienced some resorption and subsequent growth of a lower-An (An40-60) mantle and rim within a melt-rich felsic magma. These plagioclase timescales are of similar magnitude to quartz diffusion timescales calculated using C07 (mostly <1 kyr) but are several orders of magnitude shorter than quartz diffusion timescales calculated using A23, J20, and A21.

To investigate the plausibility of these scenarios we must consider the crystallization sequence of these phases. By modeling the liquid line of descent of an extracted melt with the whole pumice compositions from this study, rhyolite-MELTS predicts that plagioclase would be the first to crystallize in the rhyolite melt at the shallow pressures determined for the Aira system. This has also been demonstrated by several other studies without the use of rhyolite-MELTS (*57*, *58*). Furthermore, using a similar methodology to Pamukcu *et al.* (*10*) and Pitcher *et al.* (*13*) and assuming that heat (enthalpy) were to be lost from the extracted melt at a constant rate, our rhyolite-MELTS modeling indicates that quartz should crystallize for 13 to 67% of the time that plagioclase would be crystallizing (at 125 MPa), depending on the sample. This is most consistent with our C07 diffusion results that indicate quartz residence times that are 16 to 44% of the residence times of plagioclase.

Use of the recent, slower diffusion coefficients (A23, J20, and A21) gives quartz diffusion timescales that would imply that quartz resided within an eruptible magma for hundreds of thousands to millions of years before plagioclase began to crystallize just 2.5 kyr before eruption. Such quartz-only rhyolite magmas are never predicted by rhyolite-MELTS or observed in nature (*58*). Alternatively, if the Aira magma experienced periods of much higher temperatures, it would require less diffusion time to reach the observed profiles. However, the existence of quartz puts an upper limit on the temperatures that could have been reached because rhyolite-MELTS predicts that quartz would dissolve above 770° to 830°C, depending on the sample and storage depth (fig. S9). Even if we assume that quartz could avoid dissolution up to the maximum temperature of 830°C (requiring storage at 75 MPa, the minimum depth retrieved by our barometers), it would still require diffusion timescales of up to 66 kyr (A23), 136 kyr (J20), and 665 kyr (A21), that is, tens to hundreds of thousands of years of quartz-only magma without plagioclase. Thus, the independently derived plagioclase diffusion timescales are more consistent with short quartz residence and the diffusion coefficient of Cherniak *et al.* (*18*).

Recent work has determined a Sr diffusion coefficient that is 1.5 to 2 orders of magnitude lower (*48*) than the coefficient we used for our modeling (*59*), which could lead to plagioclase diffusion timescales that are 2 to 2.5 orders of magnitude longer, potentially up to 10^6^ years. This would be of the same order of magnitude as quartz timescales from J20 and A23 but would still be too short compared to A21 timescales. However, such long plagioclase diffusion timescales are inconsistent with the textures and compositions observed in the interiors and rims of Aira plagioclase. Most plagioclase exhibit minor variation in An content over the 200- to 500-μm distance from the core-interior boundary to the crystal edge, mostly ranging between An30 and An50, characterized by within-crystal ranges of less than 0.15 mol fraction. Few crystals have any truncated zonation or sawtooth patterns indicative of resorption events (*60*, *61*), and very few have spikes in An that are more than 0.05 mol fraction. Thus, Aira plagioclase record few signs of major changes in temperature, dissolved water (pressure), or melt composition. If the new plagioclase diffusion coefficient were to be accurate, it would require the host melt to remain in a narrow temperature range without any major disruptions or changes in composition for up to 10^6^ years, which is likely unrealistic in a system that would require continual recharge events to remain in a melt-rich state. In addition, these longer plagioclase timescales are inconsistent with absolute (U-series) ages of plagioclase crystals at silicic systems worldwide that tend to have begun crystallizing within tens of thousands of years before eruption (*14*). It should also be noted that the new diffusion coefficient was calibrated at a much higher temperature range (900° to 1200°C) and a narrower range in An (28 to 67) and would require extrapolating beyond the calibration to apply to Aira plagioclase. Although this publication adds additional uncertainty, we give several additional independent lines of evidence below that are consistent with shorter timescales.

Additional evidence of short timescales comes from the shape of quartz-hosted MIs. Because rounded MIs are disequilibrium features and diffusion at magmatic temperatures causes the inclusions to become more faceted through time, approaching an equilibrium negative crystal shape of the host crystal, previous studies have used the faceting of quartz-hosted MIs to inform on timescales (*10*, *11*). According to the conservative (slower) MI faceting equation of Gualda *et al.* (*11*), if the crystals had resided at 750°C for ∼1 Myr as implied by J20, we would expect that all MIs smaller than 550 μm should be fully faceted. We do not observe this in Aira quartz; most are rounded, with a faceting index of 1 or lower on the 0 to 2 scale defined by Boro *et al.* (*62*), wherein 2 is fully faceted (fig. S12). The lack of faceted MIs, even within the cores of most Aira quartz is inconsistent with long timescales at elevated temperatures and is more consistent with timescales of 10^3^ to 10^4^ years (*11*).

In addition, mineral textures and compositional variations imply periods of relatively rapid crystallization that is inconsistent with timescales of residence at >750°C for millions of years. Rapid growth textures, such as hopper cavity MIs and dendritic textures, as defined by Barbee *et al.* (*63*), are observed in quartz grains from most pre-AT and AT units (fig. S13). If dendritic growth textures were limited to the outer rims of quartz, these textures could still be consistent with slow growth for millions of years followed by a final stage of rapid growth. If the crystals only had rapid growth textures in the core, it could feasibly have resulted from initial fast growth followed by slow growth, potentially at elevated temperatures. However, several crystals from most Aira pre-AT and AT units contain skeletal textures throughout the core and/or interior of the crystal, indicating that the crystal experienced several episodes of rapid growth, which is more consistent with periods of heating and cooling associated with cold storage. Although there is uncertainty in the growth rates necessary to produce skeletal morphologies, it remains difficult to reconcile these rapid growth textures with long residence times of hundreds of thousands to millions of years at elevated temperatures without periods of cooling.

We also stress that Scenario-2 (warm storage, long timescales) is inconsistent with the Aira pre-AT eruptive history. Kuritani (*49*) used an amphibole geobarometer to show pressures of 127 ± 12 MPa for the 90-ka Fukuyama Pumice eruption, and our rhyolite-MELTS geobarometry results show that all magmas were likely stored at shallow depths similar to the AT magma. However, Kuritani (*49*) showed that the Fukuyama Pfa (90 ka) and Iwato Pfl (60 ka) have distinctly different Sr, Nd, and Pb isotopic signatures compared to each other and to the AT eruption and proposes that the shallow upper crustal Aira magma system was fully evacuated and replaced by new isotopically distinct magma after each of these eruptions. This isotopic difference is also echoed by the compositionally distinct dacitic pumice within the 90-ka Fukuyama Pfa that only contains trace quartz (*23*). Furthermore, the rarity of quartz 60 kyr before the AT eruption is not consistent with long AT quartz residence times of more than 10^5^ to 10^6^ years (A23 and A21, respectively).

We also suggest that it would be difficult to keep the AT magma in an eruptible state for 10^5^ to 10^6^ years without either erupting or cooling below 750°C. Rhyolites with near-eutectic compositions should crystallize to 25 wt % crystals (much higher than observed within the AT eruptions) within 1 ka (*11*) and would crystallize much faster with the assistance of hydrothermal convection (*64*). Such hydrothermal circulation within the crust surrounding the shallow AT magma should be expected because the Kagoshima Graben has been actively extending since 3 million years ago (Ma) and is characterized by modern productive geothermal fields and epithermal gold-silver deposits that date back to 2 Ma (*65*).

We hold that the long timescales of Scenario-2 require a highly specific and unlikely set of recharge conditions to stay hot (above the solidus), but not hot enough to dissolve quartz, for millions of years. For example, a magma stored at 125 MPa, as suggested by our two barometers, would need to remain between 770° and 800°C (fig. S9). The long timescales of Scenario-2 would require the AT magma to remain in these narrow temperature conditions with an eruptible viscosity, but not erupt, during the pre-AT eruptions at 90, 60, 33.5, 32.3, or 31.6 ka or any other time during a time period of 10^5^ to 10^6^ years. As discussed above, this would also require the magma to exist for much of this time containing only quartz (but no plagioclase), an assemblage that is never predicted by rhyolite-MELTS or observed in nature (*57*, *58*). Therefore, we strongly favor the short timescales of eruptible magma in Scenario-3.

### Short timescales proposed by other studies

Other studies on other caldera systems have used independent methods and crystalline phases that give short timescales that are similarly inconsistent with the timescales given by J20, A21, and A23. For example, recent work by Wang *et al.* (*39*) directly compared Mg and Sr diffusion relaxation times in plagioclase from the Cerro Galán Ignimbrite (*43*) to quartz timescales from the same unit and found that only the C07 diffusion coefficient gives similar rim timescales to plagioclase. Using Sr diffusion in plagioclase and amphibole, Lubbers *et al.* (*44*) suggest that the Youngest Toba Tuff was stored at temperatures above 750°C for only centuries. In the Bishop Tuff, Gualda *et al.* (*11*) used Ti zoning in quartz (using C07), along with MI faceting times, quartz, and feldspar crystal size distributions, and thermodynamic and heat flow modeling to suggest a short crystallization timescale of ∼500 to 3000 years. These short timescales are backed up by Chamberlain *et al.* (*12*), who determined diffusion timescales of less than 150 years in orthopyroxene (Fe-Mg) and quartz (Ti, using C07), and less than 5000 years using Ba and Sr in sanidine. Studies at other locations including Yellowstone, Santorini, Taupo, and Mount St. Helens, using crystal size distributions (*4*), Ba and Sr diffusion in sanidine (*66*), Fe-Mg diffusion in orthopyroxene and clinopyroxene (*67*–*69*), and Mg and Sr diffusion in plagioclase (*41*, *43*), consistently give timescales on the order of decades to centuries. It is possible that other magmatic systems may have evolved in diverse ways, possibly under warm storage, but for the eight distinct systems highlighted above, a myriad of independent chronometers converge on these short timescales consistent with cold storage.

Jollands *et al.* (*20*) and Audétat *et al.* (*19*) argue that their new, slower diffusion coefficients bring quartz diffusion timescales to match the total crystallization timescales given by zircon. However, we show that timescales of quartz diffusion determined for Aira using A21 and J20 are, in many cases, muchlonger than absolute zircon ages. We compiled literature values of U-Th and U-Pb total zircon residence times (defined as the oldest zircon age minus the eruption age) as well as the maximum range of zircon crystallization (oldest age minus youngest age) for other systems around the world and found zircon residence times are primarily less than 400 kyr, but range up to 700 kyr, which is much shorter than the 6- and 2-Myr timescales of quartz diffusion determined using A21 and J20, respectively (Fig. 8B). Because zircon ages represent the full crystallization time of magma and mush systems (regardless of temperature and eruptibility) and most of this time records the waxing and waning of antecrystic zircons (*14*, *38*), it is illogical to have quartz diffusion timescales, which only record periods of high temperature, that are longer than the total crystallization timescales represented by zircons. A similar conclusion is reached by Pamukçu *et al.* (*70*), who argue that the slower diffusion coefficients give quartz diffusion timescales within the Searchlight magmatic system that are not permitted by total pluton (mush) crystallization times (150 to 200 kyr) determined by zircon geochronology. Furthermore, the authors demonstrate that only the faster diffusion timescales (C07) of less than 10 kyr are of similar magnitude to zircon dates and plagioclase growth times from the extracted leucogranite melt and are more consistent with the geologic evidence.

An alternative model in which large rhyolite magmas are held at much higher temperatures, which would decrease the diffusion timescales, still renders those calculated with J20 and A21 to be unrealistic. As mentioned above, even at the maximum temperature of 830°C for this system that quartz could survive without dissolving (fig. S9), diffusion timescales of 66 kyr (A23), 136 kyr (J20), and 665 kyr (A21) would be required to reach observed profiles. Although A21 would imply quartz residence that is still longer than most zircon radiometric growth times worldwide, these elevated temperatures allow for timescales implied by the other two diffusion coefficients that are within the range of globally compiled zircon timescales (Fig. 8B). However, our calculated zircon saturation temperatures demonstrate the magma could not have spent extended durations above 770°C because zircon would have begun to dissolve above this temperature and caused the glass Zr to be much closer to the whole-rock Zr than is observed in most Aira pumice clasts (figs. S1 and S4).

Last, the warm storage model is inconsistent with geophysical observations worldwide. On average, there have been 1.4 to 2.0 magnitude 8 (10^15^ kg erupted) eruptions per million years, worldwide, since 13.5 and 6 Ma, respectively (*71*). Therefore, the Poisson probability of one of these large eruptions occurring in the next million years is 75 to 86% (*71*). If large volumes of silicic magma are held in an eruptible state for more than a million years before eruption (as suggested by J20 and A21), then we would expect that there should currently be at least one large reservoir of melt-rich silicic magma somewhere on Earth. However, no geophysical studies to date have revealed a melt-rich magma body >100 km^3^ (*15*, *16*). Furthermore, Yellowstone, the most geophysically studied caldera system in the world, has been shown by most studies to have a magma system that contains less than 20% melt (*17*). Considering that the three most recent CFEs at Yellowstone have been separated by ∼700 kyr, and the last eruption was ∼630 ka, the current lack of a large volume of melt-rich rhyolite is inconsistent with the theory of long timescales of warm storage. It is important to note, however, that future improvements in spatial resolution may be able to estimate melt proportions more confidently and may reveal complex systems of numerous melt rich bodies, which are currently seen as low melt fractions averaged over a large volume. Nevertheless, recent surveys have failed to detect a single large volume of melt-rich magma that would be consistent with the warm storage hypothesis.

We conclude that considering that submillennial timescales of Aira quartz using C07 are consistent with independent timescales determined by numerous studies worldwide using MI faceting, crystal size distributions, diffusion in pyroxene, plagioclase, and sanidine, and absolute age dating of zircons. Thus, we suggest that the Cherniak *et al.* (*18*) diffusion coefficient is more realistic, and that the rhyolite magmas that feed caldera eruptions around the world are commonly held in cold storage and are eruptible for only centuries.

### Why was only the fourth Aira eruption in 3500 years so large?

We have provided compelling evidence that Scenario-3 is the most likely scenario for the Aira caldera system, but two logical questions follow from this conclusion. If each of the pre-AT and AT magmas had an eruptible viscosity for less than ∼1000 years, which is less than the repose times between these eruptions, then why was only the fourth eruption in 3500 years so large? Furthermore, how did 400 km^3^ of eruptible magma amass in such a brief period of time? We suggest, based on the eruption of a less evolved population of gray pumice in the Osumi Pfa, higher Osumi eruption and zircon saturation temperatures, and distinctly higher Li abundance in Ito ignimbrite plagioclase cores, that the AT eruption likely involved the recharge of hotter, more fluid-rich magma, which allowed for more mush rejuvenation and the rapid production of 400 km^3^ of melt-rich magma.

Although the Osumi gray pumice (Osumi-G) clasts account for less than 1% of the volume of pumice within the Osumi pumice fall deposit (*23*), the unique composition of these pumice clasts hints at an important difference in the petrogenesis of the AT magma. The pumice glass of Osumi-G has slightly lower SiO_2_ (76.3 to 77.9 wt % versus 77.2 to 78.5 wt % SiO_2_), lower incompatible element concentrations of Rb, Y, Nb, Cs, Ba, REE, U, and Th, and higher concentrations of compatible Al_2_O_3_, FeO, CaO, and Sr (Fig. 2). Whole-rock data from the literature show similar distinctions in Osumi-G compositions, as well as elevated MgO, P_2_O_5_, Cr, and Y, which are not consistent with the clear linear trends exhibited by all other pre-AT and AT whole-rock compositions (fig. S1) (*23*, *49*). Our geobarometry work shows that the Osumi-G magma could have been stored at deeper levels (up to 200 to 300 MPa) than the rhyolitic magma (75 to 160 MPa) of the Osumi eruption, although these pressures would be maxima if quartz were not saturated, as discussed above. Osumi-G compositions form mixing trends toward the white Osumi pumice and some gray pumice clasts are visibly banded with both white and gray glass. In addition, the Osumi-G pumice has been shown to be isotopically distinct from the rhyolite pumice from Osumi or any pre-AT eruption (*49*). This indicates the involvement of a distinct, deeper, and less evolved rhyodacite magma that experienced incomplete mixing with the more common rhyolite composition before eruption. Nishihara *et al.* (*40*) also analyzed rare andesitic scoria found within the Ito ignimbrite and suggests, based on major element and isotopic data, that andesitic magma was injected into an upper crustal mush, which mixed with the resident felsic melt and crystallized to produce the white AT pumice; additional injection of the andesite and variable degrees of mixing with the rhyolite led to the production of the observed gray pumice at the bottom of the magma reservoir. This hypothesis would be consistent with pronounced increase in An (∼An40 to An80-94) that we observe in the interior of many plagioclase crystals of the white pumice, and the hot andesite would provide the recharge driver for mush rejuvenation.

In addition, Osumi Pfa magma was stored and erupted at higher temperatures than pre-AT magmas. Although most (90%) of pre-AT zircon saturation temperatures are between 721° and 771°C, the crystal-poor Osumi pumice is at the higher end of this range (756° to 764°C), and the gray pumice is much hotter (745° to 795°C) (fig. S4). Magnetite-ilmenite eruption temperatures calculated by Nishihara *et al.* (*40*) also indicate that the gray pumice erupted at much higher temperatures (836° to 860°C) compared to white pumice from the same unit (762° to 822°C). Pre-AT eruption temperatures, calculated using the same methodology, are much cooler (730° to 800°C). Considering that the magma was likely of near-eutectic composition and moderately wet, small increases in temperature would lead to large degrees of remobilization of a crystalline mush (*72*).

**Fig. 9.**
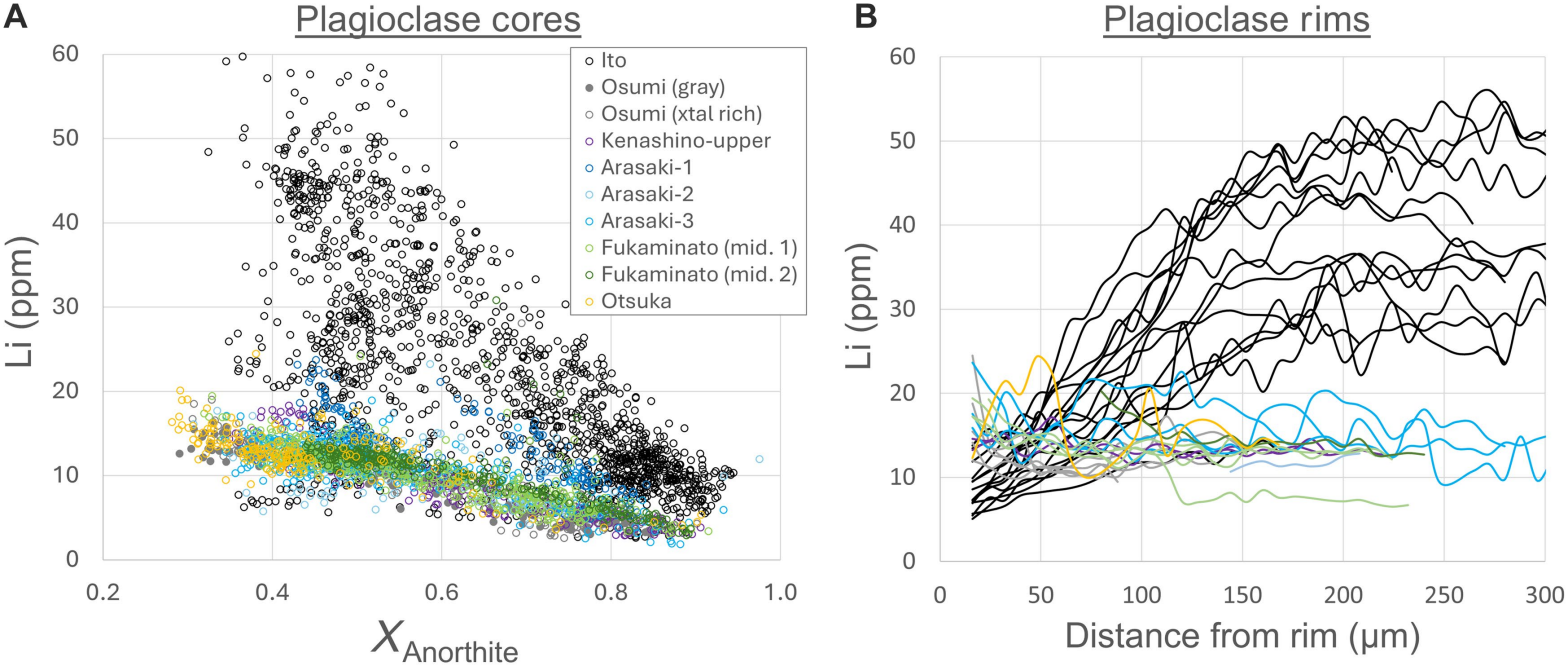
Lithium in plagioclase. (**A**) Li (ppm) versus An (mol %) of plagioclase cores. Lithium content from Ito ignimbrite is much higher for a given An content than Osumi Pfa or any pre-AT units. (**B**) Whereas Osumi Pfa and pre-AT plagioclase Li content stays relatively constant to the rim (with a slight increase), those from the Ito ignimbrite have Li contents that start much higher and then decrease toward the rim with lower rim Li contents than other units.

Another piece of evidence is the Li content of plagioclase from Ito ignimbrite being significantly higher than in other units. Although analyses of plagioclase cores from all pre-AT and Osumi pumice clasts (besides some from A-08) lie along the same tight negative trend of Li versus An, plagioclase cores from Ito ignimbrite have two to three times higher Li (up to 60 ppm versus 20 ppm) for a given An content (Fig. 9). Because the plagioclase An contents, storage pressures, eruption temperatures, and zircon saturation temperatures of Ito ignimbrite and pre-AT units are similar, only differences in Li content of the melt could cause the observed differences in plagioclase Li. Observed plagioclase compositions are consistent with melt concentrations of 200 to 290 ppm for the Ito ignimbrite compared to only 50 to 80 ppm from the pre-AT eruptions, using the range in An-dependent partition coefficients from Cabato *et al.* (*73*). Because Li diffuses rapidly in plagioclase (*74*), the Li content of the melt does not necessarily need to be high during the crystallization of the plagioclase cores; it is possible that late-stage Li enrichment of the melt caused Li to diffuse into plagioclase just before eruption. However, we know that the host magma must have been enriched in Li before final ascent because we observe a clear exponential decrease in Li toward the rim for all Ito plagioclase (Fig. 9). We argue that this shows that Li diffused out of plagioclase into a depleted melt as it strongly partitions into a vapor phase or magmatic brine during decompression (*75*, *76*). Ultimately, this shows the Ito plagioclase cores preserve evidence of a preeruptive melt with much higher Li than pre-AT magmas. Li enrichment in plagioclase is also observed at Mt. St. Helens in the early stages of both the 1980 to 1986 and the 2004 to 2008 eruptions (*77*), which has been suggested to represent the buildup of volatiles transferred from deeper parts of the magma system (*76*). However, there is considerable variability in the literature regarding the degree to which Li is partitioned into the fluid phase at depth (*78*, *79*), so it is unclear at this time whether Li enrichment in the Ito melt is consistent with the transfer of more fluids into the defrosting mush.

Although the determination of the exact processes that led the AT eruption to expel orders of magnitude more magma than the preceding eruptions is beyond the intended scope of this study, we speculate potential causes based on the evidence above. We propose that hot fluid-rich recharge magmas may have ponded below the AT mush, experiencing rapid crystallization and vapor exsolution upon emplacement. As a result, the recharge magma transferred both enthalpy and supercritical fluids (potentially enriched in Li) to the mush, thereby reducing the liquidus and causing “chemical superheating” of the mush and enabling greater degrees of partial melting (*80*).

The combination of more fluids and higher temperatures of recharge could have led to relatively rapid production of eruptible AT magma. Spera and Bohrson (*72*) use thermodynamic modeling to show that a recharge magma could unlock 400 km^3^ of wet magma by enthalpy transfer alone in less than 1 kyr as long as the recharge magma were injected as 80 individual batches. Addition of volatiles to the magma would decrease timescales by both lowering the mush liquidus (*80*) and providing more advection of heat by gas sparging (*6*).

In summary, we conclude, based on the unique presence within the AT eruption of less evolved pumice, higher eruption temperatures, and higher Li content in plagioclase, that the recharge of hotter fluid-rich magma may have played an important role in causing a larger degree of rejuvenation of a crystal-rich magma mush to produce the 400-km^3^ melt-rich AT magma. Plagioclase zonation, commonly exhibited as patchy An40 cores, mantled by An80-94 interior, and then 200- to 500-μm rims of nearly constant An40 compositions, shows that a major dissolution event was followed by less than 2.5 kyr of crystallization with no additional major resorption or recharge events. The melt-rich magma produced by this rejuvenation event did not immediately erupt. Instead, it was mobilized, along with a proportion of partially resorbed plagioclase and quartz, ascending to a shallow storage region, and crystallized for up to 2.5 kyr before eruption. The final triggering mechanism and ascent timescales are beyond the scope of this paper, but ubiquitous resorbed rims of Osumi quartz suggest that a high-temperature recharge may have been the driver (*76*).

We investigated the timescales and storage conditions of four pre-caldera eruptions and the caldera-forming (AT) eruption from Aira to weigh in on the critical debate over whether eruptible silicic magmas are held in warm storage for extended periods of time (>100 kyr) or primarily held in a crystal-rich mush that is rejuvenated only centuries before eruption.

All eruptions are high-Si rhyolites (77.5 to 78.3 wt % SiO_2_) with minor differences in major and trace element compositions of glass. Because MI H_2_O-CO_2_ contents and the rhyolite-MELTS geobarometer give similar storage pressures (75 to 175 MPa), then either a large volume of eruptible magma was left untapped during the prior eruptions, or 400 km^3^ of eruptible magma was amassed in less than 1 kyr. Quartz diffusion chronometry gives similar residence times (<1 kyr, using C07) for all eruptions and there is no clear increase up-section as would be expected if the CFE magma was residing during previous eruptions.

Despite new Ti-in-quartz diffusion coefficients (*19*–*21*) that would imply that Aira magma would have been held in a melt-rich state (>750°C) for up to 400 kyr to 4 Myr, we hold that our study provides abundant converging evidence that these timescales are unlikely and that the submillennial timescales resulting from the faster C07 Ti diffusion coefficient (*18*) is more realistic (*39*, *70*). We demonstrate, using an independent diffusion chronometer, that plagioclase residence must have been less than 2.5 kyr. If the longer quartz residence timescales were correct, it would require the magma to contain quartz but not plagioclase for to 99.4 to 99.95% of the residence time, which is an assemblage never modeled or observed in nature. This would also lead to eruptible timescales that, in some cases, exceed the total magma crystallization timescales determined by zircon radiometric dating. Furthermore, the longer timescales would be inconsistent with observed rapid crystal growth textures and unfaceted MIs. We also argue that long timescales are unlikely because they would require the 400-km^3^ eruptible AT magma to remain specifically between 750°C (rheological lock-up) and 830°C (above which quartz fully dissolves) for hundreds of thousands to millions of years without being tapped during any of the previous eruptions, including the several pre-caldera eruptions demonstrated in this study to have had magma stored at similar shallow depths. Because similar conclusions of short magma storage using multiple independent chronometers have been reached by many previous studies at caldera systems worldwide, we strongly favor the cold storage model being more typical for large silicic systems.

We find three key differences in the AT samples that may help to explain why only the fourth eruption in 3.5 kyr was catastrophic: (i) a population of slightly less evolved gray pumice (76.5 to 77.2 versus 77.5 to 78.3 wt % SiO_2_), (ii) higher Fe-Ti oxide eruption temperatures (780° to 850°C versus 730° to 800°C), and (iii) higher Li abundance (60 versus 15 ppm) and Li/albite ratio within plagioclase interiors, consistent with Li (200 to 290 ppm) in the AT melt (versus 50 to 80 ppm in pre-CFE erupted magma). We suggest that a recharge of hotter, fluid-rich mafic magma rejuvenated a much larger mush volume than during pre-AT eruptions, amassing 400 km^3^ of eruptible, volatile-rich magma in less than 1 kyr.

## MATERIALS AND METHODS

### Radiocarbon dating

We determined the radiocarbon ages of three pumice fall deposits (Otsuka Pfa, Fukaminato Pfa, and Kenashino Pfa). These ages were obtained from the paleosol directly underlying these deposits. Bulk paleosol samples were cleaned chemically, using HCl acid treatment, and combusted to CO_2_. The purified CO_2_ was then reduced to graphite. The ^14^C/^13^C ratio of the graphite was then measured using a Tandetron accelerator (NEC Pelletron) mass spectrometer, at the Institute of Accelerator Analysis Ltd., using a similar method to that of Nishihara *et al.* (*81*). The conventional ages were calibrated using the IntCal 20 program (*82*).

### Sample collection, selection, and preparation

We collected pumice clasts from five pre-AT pyroclastic units (Iwato Pfl, Otsuka Pfa, Fukaminato Pfa, Arasaki Pfl, and Kenashino Pfa) and two AT pyroclastic units (Osumi Pfa and Ito ignimbrite) during October 2019. Clasts were collected from type localities or from locations mapped and described by Nagaoka *et al.* (*27*). No permission was necessary to collect these samples. For each pumice fall unit (Pfa), we sampled at least 20 pumice clasts from each clearly defined horizon, as identified by a marked change in clast size, lithic abundance, or grading. For pyroclastic flow units (Ito, Arasaki, and Iwato), we focused on sampling the major flow unit. For all pyroclastic flow and fall units, we were careful to collect at least 20 samples of each macroscopically identifiable pumice population. For example, we collected 20 each of crystal-rich, crystal-poor, and gray pumice populations within the Osumi pumice-fall deposit.

To minimize sampling bias and to ensure that magmatic heterogeneity was characterized, we selected 12 to 15 pumice clasts from each subunit or population for compositional analysis. We avoided pumice clasts that were hydrated or appeared to be hydrothermally altered. We sampled a 0.25- to 1-cm-diameter portion of the interior of each pumice clast and mounted these interior fragments in epoxy using a vacuum impregnation method, polished the hardened epoxy mounts, and carbon coated them for analysis.

### Glass major and trace element analysis

We collected major element compositional data at Vanderbilt University using an Oxford X-max 50-mm^2^ EDS system attached to a Tescan Vega 3 LM Variable Pressure SEM. We used the same analytical conditions for glass analysis as provided in Pitcher *et al.* (*13*) including a 15-kV accelerating voltage, 2.7- to 3.4-nA absorbed current, and 15 s of live analysis time. We analyzed 10 to 20 spots for each pumice clast, each larger than 30 μm by 30 μm, where possible, and quantification was performed using the AZtec software from Oxford Instruments, using internal standards for calibration. We assessed the quality of results by analyzing fused glass of the US Geological Survey (USGS) rhyolite reference standard (RGM-1) as a secondary standard repeatedly throughout analysis of unknown samples. The precision (1 SD) of EDS measurements was between 0.2 and 4.4% for most oxides, with lower precision of lower abundance (<0.3 wt %) oxides such as TiO_2_, MgO, and MnO. Accuracy compared to the USGS published value of RGM-1 was within 4% for all high abundance elements (>0.3 wt %) except CaO, which was 8.3% higher than the published value. A similar conclusion using the same instrument is demonstrated by Gualda *et al.* (*83*). All EDS data including replicate analyses of the secondary standard can be found in data S1.

We collected glass trace element data at Vanderbilt University, using the Photon Machine Excite 193-nm excimer laser ablation system and Thermo Fisher Scientific iCAP Qc quadrupole LA-ICPMS. We used an identical procedure to Pitcher *et al.* (*13*) to collect 7 to 15 spots, each 35 μm by 45 μm in size, for each pumice clast, being sure to analyze the same location as selected for EDS analysis. ^29^Si was the internal standard, and we used NIST-610 as the calibration standard and ATHO-G, NIST-612, and RGM-1 (USGS rhyolite of Glass Mountain) as secondary standards. Precision based on secondary standards (1 SD) was better than 10% for 22 of the 35 elements and better than 15% for 29 elements. Accuracy was within 15% for 24 and 30 elements compared to the published values of the RGM-1 and ATHO-G secondary standards, respectively. All LA-ICPMS data including replicate analyses of the secondary standards can be found in data S1.

We removed outlier analyses that had Al, Ca, Na, Fe, Ti, Sr, or Zr contents that were further than 1.5 times the IQR from the median. These likely represent analyses of crystals such as plagioclase, pyroxenes, or Fe-Ti oxides.

### Statistical analysis

We used an agglomerative hierarchical clustering method (*84*) to assess the compositional variability of pumice within and between each unit and to choose pumice populations for further analysis. This multivariate statistical method iteratively groups the two pumice clasts with the most similar multivariate compositions at each step until all clasts are joined together. The linkage method was the standardized Euclidean distance between unweighted centroids (UPGMA), and we used the upper-tail cutoff rule of Mojena (*85*) to objectively determine the step at which the clustering should stop, allowing us to form the final pumice groupings. For this work, we used the subset of major and trace elements (SiO_2_, Al_2_O_3_, CaO, FeO, Sc, Zr, Nb, Nd, and Th) that are best determined with regard to standards and are less susceptible to fluid mobility as to avoid the effect of chemical weathering on pumice glass compositions (*86*). On the basis of these statistics, we chose one representative pumice clast from each compositionally distinct pumice population of each sample from which to extract plagioclase and quartz for further analysis.

### Plagioclase trace element profiles

One representative pumice clast from each compositional group of each sample was carefully crushed in a plastic beaker using a wooden dowel, and quartz and plagioclase grains were separated from glass fragments by winnowing in water within a glass beaker. Crystals were selected under a binocular microscope, mounted in epoxy resin, and polished for SEM analysis.

We collected backscattered electron (BSE) images of between 10 and 30 plagioclase crystals per sample to characterize growth and resorption textures and to identify regions for trace element analysis. We collected trace element data across major zoning boundaries seen in the BSE images, using the LA-ICPMS methodology described above for glass analysis. This involved a series of consecutive spots, each measuring 7 μm by 60 μm, progressing rimward in a line of spots that were perpendicular to the zonation boundary (*14*). The transects spanned distances ranging from 80 to 720 μm, always encompassing at least 40 μm on both sides of a zone boundary. In addition to collecting trace element transects, we made sure to gather trace element data from all major core, rim, and interior zones of crystal, as identified by BSE images, ensuring a representative sampling of each crystal. In addition, we conducted full core-rim transects for several plagioclase crystals, resulting in a total of 227 trace element transects, with 13 to 46 transects per sample. To estimate the anorthite content in situ, we used the Ca/Si ratio determined by the LA-ICPMS and the stoichiometry method used by Cooper and Kent (*14*).

### Whole-rock compositions

Whole-rock chemical compositions were analyzed by a wavelength-dispersive, x-ray fluorescence spectrometer (XRF; PANalytical Axios Advanced) in the Geological Survey of Japan (GSJ), AIST. The XRF is equipped with a Rh end window-type, x-ray tube with a maximum 4-kW power.

For the major element analysis of the rock samples, a glass-bead method was used. A powdered sample (0.5 g) was mixed with tetraborate dilithium powder; the dilution ratio of sample against the flux was 1:10. Approximately 0.018 g of lithium bromide was added as a releasing agent to remove glass beads from the Pt crucible.

The conditions of acceleration voltage and tube current of the x-ray generator were set at 50 kV and 50 mA for detection of the Kα lines of major elements (Na, Mg, Al, Si, P, K, Ca, Ti, Mn, and Fe), 32 kV and 125 mA for the Kα line of Sc, 50 kV and 80 mA for Kα lines of V and Cr, 60 kV and 66 mA for the Kα lines of Co, Ni, Cu, Zn, Rb, Sr, Y, Zr, Nb, and Th, and 40 kV and 100 mA for the Lα line of Ba. A flow proportional counter was used for the elements between Na and Ti, a duplex detector system was used for Mn and Fe, and a scintillation detector for the elements larger than Co. Calibration was conducted with 12 standard reference samples (JA-1, JA-2, JA-3, JB-1a, JB-2, JB3, JG-1a, JG-2, JGb-1, JGb-2, JP-1, and JR-1) issued by the GSJ. Background and line overlapping corrections were applied before calculation of the calibration lines. The calibration lines were prepared with full matrix correction, using classic theoretical alpha values. The precision (reproducibility) of analysis was evaluated with 10 repeat analyses of a glass bead of JB-1a standard (data S1). The relative SDs were less than 1% for all elements.

### MI volatile contents

Using a microscope, we handpicked quartz crystals containing MIs that were visible in mineral oil. We mounted each quartz crystal onto a glass slide using crystal bond and only polished (using 15-, 9-, 6-, and 3-μm grit sizes) until the maximum width of the crystal was exposed. We then heated the slide, flipped the crystal in the crystal bond, let it solidify overnight, and then repeated the polishing procedure. As a result, MIs were not exposed on either side, and each doubly polished crystal wafer contained 1 to 10 MIs that we could analyze. Crystal wafers ranged between 200 and 700 μm in thickness.

To collect H_2_O and CO_2_ concentrations from these unexposed MIs using FTIR, we followed the methodology developed by Tollan *et al.* (*87*). Because we do not have to double polish to expose only one inclusion, this method has the advantage that we can measure many MIs per quartz host. This method also reduces the inevitable attrition of samples that occurs when working with thin (∼10 to 50 μm), doubly exposed inclusions. For each MI, we carefully measured the thickness of the quartz + MI seven times and used the average value. The SD was used to calculate uncertainty in volatile content. MIs were all less than 50% of the total thickness, as advised by Tollan *et al.* (*87*). Using this approach, we calculated the percent difference in the quartz overtone absorbance at 2136 cm^−1^ (integrated between 2111 and 2171 cm^−1^) in spectra collected through an unexposed MI and the absorbance through an adjacent region of pure quartz to get the percent of the total thickness that is MI. Then, by multiplying by the total thickness, as measured using a calibrated digital micrometer, we calculated the MI thickness.

We collected absorbance spectra by unpolarized transmission FTIR spectrometry at the Lamont Doherty Earth Observatory, using a nitrogen-cooled, Thermo Fisher Scientific Nicolet iN10 MX Infrared Imaging Microscope. We collected data for infrared wavenumbers between 1000 and 6000 cm^−1^ with a 4-cm^−1^ resolution, and the sample compartment was continuously purged with dry air. We chose the maximum aperture size for each inclusion such that we avoided the edge of the inclusion, vapor bubble, or microlite. Each spectrum was the average over 128 or 256 scans depending on the aperture size. For each MI, we sequentially performed a background measurement, then the enclosed MI, and then the adjacent inclusion-free quartz, all with the same aperture and scan speed.

H_2_O concentration was calculated using equation 8 from Zhang *et al.* (*88*), using the nonintegrated absorbance at 5245 and 4510 cm^−1^, with straight baselines drawn between 5348 and 4813 cm^−1^ and 4660 and 4259 cm^−1^, respectively. CO_2_ concentration was calculated with the method from Behrens *et al.* (*89*), using the absorbance at 2345 cm^−1^ and a straight baseline drawn between the minimum values on either side of this peak between 2300 and 2370 cm^−1^. Uncertainty was propagated using uncertainties from: measured total thickness, the coefficients in equation 8 from Zheng *et al.* (*88*), and the molar coefficient from Behrens *et al.* (*89*).

### Geobarometry and geothermometry

We estimated storage pressures for all units using nonoutlier major element glass compositions and following the rhyolite-MELTS geobarometer procedure given in Gualda and Ghiorso (*30*). The barometer determines the pressure at which the saturation surfaces of the observed major phases (quartz and plagioclase) intersect for each melt (glass) composition. We investigated a temperature range of 1100° to 730°C, with steps of 1°C, and a pressure range of 400 to 25 MPa in 25-MPa steps. Oxygen fugacity was constrained to FMQ+0.5 for all runs, which is within the range of NNO−0.5 to NNO determined for Aira Fe-Ti oxides by Geshi *et al.* (*23*) using the geothermometer of Ghiorso and Evans (*90*). We used an identical procedure as Pitcher *et al.* (*13*); additional details of the methodology can be found in that paper.

We calculated saturation pressures of MI H_2_O and CO_2_ concentrations using both MagmaSat (*31*) and VolatileCalc (*33*). To calculate pressures using MagmaSat, we used the Python program VESIcal (*91*). Although we used the average glass composition as an input for MagmaSat, we found that using the whole-rock composition only decreases the calculated saturation pressure by less than 5 MPa. We can therefore be confident that the MI composition would not lead to noticeably different pressures. We calculated pressures using 770°C, which is intermediate between temperatures of quartz saturation (∼800°C) and rheological lock-up (>60% crystals) (∼760°C) as estimated for Aira whole-rock compositions using rhyolite-MELTS. In addition, we accounted for uncertainty in volatile contents (due to uncertainty in thickness) and temperature and by calculating, for each MI, a lower pressure bound using the high temperature (800°C) and minus-2σ values of H_2_O and CO_2_ and a higher pressure bound using the low temperature (750°C) and plus-2σ values of H_2_O and CO_2_. To estimate pressures from VolatileCalc, we calculated 50- to 500-MPa isobars at 25-MPa intervals, for a rhyolite at 770°C. We then estimated the equilibration pressure using the relative position of each inclusion’s H_2_O and CO_2_ content to the nearest isobars. Uncertainty from visually estimating pressures is minute compared to uncertainty in H_2_O, CO_2_, and the isobars themselves.

We calculated zircon saturation temperature with the Watson and Harrison (*34*) geothermometer, using the average major element and Zr content for each pumice clast. We also calculated individual temperatures for each glass spot for which we had collected both datasets. In addition, we calculated zircon saturation temperatures using the thermometers of Boehnke *et al.* (*35*) and Gervasoni *et al.* (*36*). However, we focus on the Watson and Harrison (*34*) temperatures because they tend to give more realistic temperatures (i.e., above solidus), as discussed by Pitcher *et al.* (*13*).

### Quartz diffusion modeling

CL imaging was done at Vanderbilt University using a Tescan Vega 3 LM Variable Pressure SEM equipped with a Tescan panchromatic CL detector. Full-crystal 16-bit CL grayscale images for 20 to 25 quartz crystals per sample were captured using a working distance of 15 mm, a beam current of ∼17 nA, and a dwell time of ∼1 ms per pixel. The field of view for these full-crystal images ranged from 700 to 3000 μm, depending on the size of the crystal. For diffusion timescale analysis, we captured higher-resolution (0.4 μm/pixel) CL images of any core zone boundary or rim zone boundary that had sufficient CL contrast. This magnification minimizes pixel size without being smaller than the incident beam diameter of ∼0.4 μm. Because the SEM scans left to right, we always kept the brighter CL zone on the right side to avoid afterglow, which causes tails of high intensity that leads to inherent blurring and averaging of CL intensity.

We used an IDL script to quantify changes in CL intensity along a line perpendicular to each selected zonation boundary. The length of these lines was carefully selected to ensure that the CL intensity reached a stable level on both sides. Following the procedure outlined by Gualda and Sutton (*38*), we used an IDL script to draw five CL intensity profiles on each side of the chosen line, resulting in a total of 11 parallel profiles for each selected boundary. To calculate the residence times of each, we adopted the same approach as Gualda *et al.* (*32*) and Gualda and Sutton (*38*), which involved using a 1D diffusion model using Fick’s second law where the diffusion coefficient is constant. The analytical solution to this diffusion equation contains the complementary error function (*92*), assumes an initial step function in Ti between two semi-infinite zones of a quartz crystal, each initially having a constant, but different, concentration of Ti.

We use a similar methodology as in (*10*, *13*) to estimate the percentage of the total crystallization time that quartz was crystallizing within each rhyolite. We use rhyolite-MELTS to model a cooling magma with the whole-rock compositions in this study and divide the change in enthalpy (Δ*H*) from the liquidus to quartz saturation by the total Δ*H* from the liquidus to the observed crystal percentage. Assuming that the cooling magma body loses enthalpy at a constant rate then this ratio gives the percentage of total rhyolite crystallization time that would have been recorded by quartz.

### Plagioclase diffusion modeling

Observed Mg and Ba compositional profiles of Aira plagioclase are unsuitable for diffusion modeling. In Aira plagioclase, Mg correlates positively with An content in the low-An regions and correlates negatively with An in the high-An regions, forming a “kink” around An60 (fig. S8). This reflects the change in partitioning behavior of Mg in plagioclase at the transition between the I1 and C1 domain, which tends to be around An60, as discussed by Mutch *et al.* (*93*). Because the core boundaries span across this inflection point, it is harder to confidently model the equilibrium conditions. In addition, because Mg is incompatible in plagioclase, it is difficult to discern whether the observed positive relationship between Mg and An in the low-An zones of plagioclase is the result of growth or diffusion (*44*, *46*, *94*). Last, Mg diffuses ∼40 times faster than Sr in An_32_ plagioclase at 750°C (*59*, *95*) so Mg profiles would reach equilibrium faster, after ∼10^3^ years (*44*), inhibiting comparisons to the longer quartz timescales suggested by the new Ti_Qtz_ diffusion coefficients.

No sanidine is observed in any of the samples, so Ba is incompatible and increases in concentration in the melt during crystallization. Fractionation would cause a negative correlation of Ba with anorthite within plagioclase. We observe a tight negative exponential correlation between An versus Ba (*r*^2^ = 0.79). However, because such a relationship is also expected by normal equilibrium partitioning (*47*) and by diffusive reequilibration (*96*), the initial profile closely matches the equilibrium profile and we cannot confidently model diffusion timescales.

Instead, we focus on Sr diffusion in plagioclase. Because Sr is compatible in plagioclase, during fractionation, the melt will become depleted in Sr as plagioclase decreases in An mol %, leading to a positive correlation between Sr and An. However, equilibrium partitioning and diffusive reequilibration should cause a negative correlation. If observed Sr is positively correlated with a change in An, it is simple to establish initial Sr conditions and use a finite difference methodology to model diffusion time that fits the observed profile (*41*, *43*, *44*).

In the rare cases (*N* = 3) in which the observed Sr profile within the core was positively correlated with An content, we could use the 1D finite difference modeling approach of Schlieder *et al.* (*41*) and Lubbers *et al.* (*43*). The positive correlation between Sr and An allowed us to confidently choose initial conditions of a step function that brackets the minimum and maximum Sr values. We followed an identical 1D diffusion forward modeling procedure as Schlieder *et al.* (*41*), using the author’s Jupyter notebook Python script, which uses the diffusion equation of Costa *et al.* (*96*) and the An-dependent diffusion coefficients of Giletti and Casserly (*59*). We compared results using the Sr partition coefficients from three studies: Nielsen *et al.* (*47*), Bindeman *et al.* (*45*), and Dohmen and Blundy (*46*). The model boundary conditions assume an infinite reservoir where the rim-most point is in equilibrium with the host melt and thus have a fixed Sr content at the rim. We modeled the diffusion profile at 750° and 800°C for timescales of 1 to 100,000 years. The modeled timescale with the minimum chi-square value was selected as the best fit modeled timescale, and this process was repeated 1000 times, varying the Sr content by the analytical uncertainty for each Monte Carlo simulation, giving us an average time and uncertainty. For each core profile, we completed this procedure for several different initial profile conditions, varying the step positions and min and max Sr, as a sensitivity analysis.

However, in most cases, the observed Sr was negatively correlated with An, which makes it impossible to confidently estimate the initial profile. Observed Sr profiles within plagioclase cores tend to be nearly flat, often varying by less than 100 ppm, despite sharp changes in An, between ∼An40 and ∼An70-90. Such substantial changes in An mean that equilibrium Sr profiles should have Sr changes of 600 to 1000 ppm, using partition coefficients from Nielsen *et al.* (*47*) and glass (80 ppm) and whole-rock (180 ppm) Sr values, respectively. Therefore, observed Sr profiles are not in equilibrium with the range of possible melt values (bracketed by glass and whole-rock). In contrast, rim transects always show a positive slope, making it much easier to assume initial conditions and model diffusion using finite difference modeling approach of Schlieder *et al.* (*41*).

Although we may not be able to determine the best fit diffusion timescale using more traditional approaches, we can establish maximum timescales of diffusion using a different forward modeling approach, adapted from Cooper and Kent (*14*). We take advantage of the fact that, if a plagioclase crystallizes in a fractionating melt (decreasing Sr and An) and consequently begins with a strongly positive slope of Sr versus An, diffusive reequilibration will cause the slope and the correlation to move progressively toward a strongly negative trend until reaching equilibrium (*14*). We used the 1D forward modeling procedure (*41*) discussed above to forward model Sr diffusion for several initial profiles, each with strong positive correlations that are similar to observed rim profiles. We can then compare the slope and correlation coefficient (*r* value) of the forward models to the observed values for plagioclase cores to estimate the maximum diffusion time. These are maxima because they assume diffusion occurred at only 750°C and assume that plagioclase started much further from equilibrium (more positive slope) than any observed rim profiles, so that, if the true initial profile were to have had a smaller slope, it would have required a shorter diffusion time to reach the observed slope.

We compare the observed slopes to the slopes expected if plagioclase had reached equilibrium within a melt with Sr between the observed glass and whole-rock values (80 and 180 ppm, respectively). For each melt composition, we calculate the expected Sr at each An content by multiplying the melt Sr by the partition coefficient at that *X*_An_ (0.2 to 0.95 at increments of 0.5). We repeat this process using each of three partition coefficients (*45*–*47*). We then fit a linear regression to these curves over the interval between *X*_An_ = 0.5 and 0.8, which is the most commonly observed An range within the cores of Aira plagioclase. We then compare these equilibrium Sr/An slopes to slopes observed within Aira plagioclase core and rim transects. For the *K*_d_ of Dohmen and Blundy (*46*), we assume a constant temperature of 750°C and a melt SiO_2_ of 75 wt %. Using higher temperatures leads to even steeper equilibrium profiles.

## Supplementary Materials

### The PDF file includes:

Figs. S1 to S13 Table S1 Legends for data S1 to S7 Data S8 References
