# Supplementary material for: Submillennial timescales of eruptible rhyolite magma: Converging evidence from Aira Caldera, Japan
Source: Sci Adv. 2026 Aug 7;12(32):eaea2075. doi: 10.1126/sciadv.aea2075 (PMC13450310; doi:10.1126/sciadv.aea2075)
Supplement: Supplementary file 1 [file sciadv.aea2075_sm.pdf]

Science Advances
AAAS

Supplementary Materials for

Submillennial timescales of eruptible rhyolite magma: Converging evidence from Aira Caldera, Japan

Bradley W. Pitcher *et al*.

Corresponding author: Bradley W. Pitcher, bradwpitcher@gmail.com

Sci. Adv. **12**, eaea2075 (2026)
DOI: 10.1126/sciadv.aea2075

The PDF file includes:

Figs. S1 to S13
Table S1
Legends for data S1 to S7
Data S8
References

Other Supplementary Material for this manuscript includes the following:

Data S1 to S7

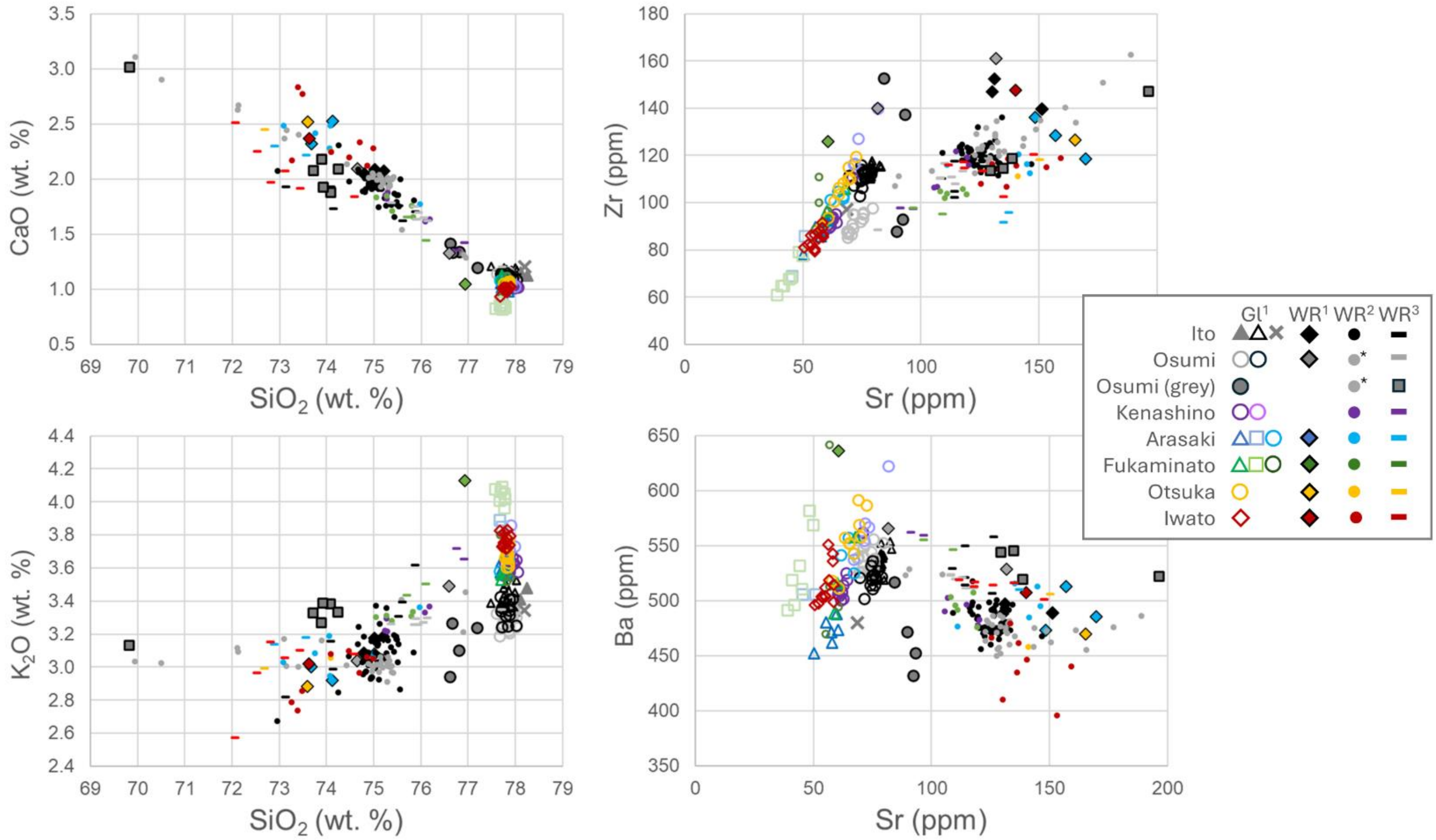

Fig. S1.

Bivariate plots comparing glass data (Gl) and whole pumice (WR) data from this study and from the literature. Sources are shown in the legend as superscripts denoting: [1]This study, [2]Geshi et al. (23), [3]Kuritani, (49). Note that Geshi et al (23) did not separate grey from white pumice within Osumi and thus are shown the same grey circles in this figure.

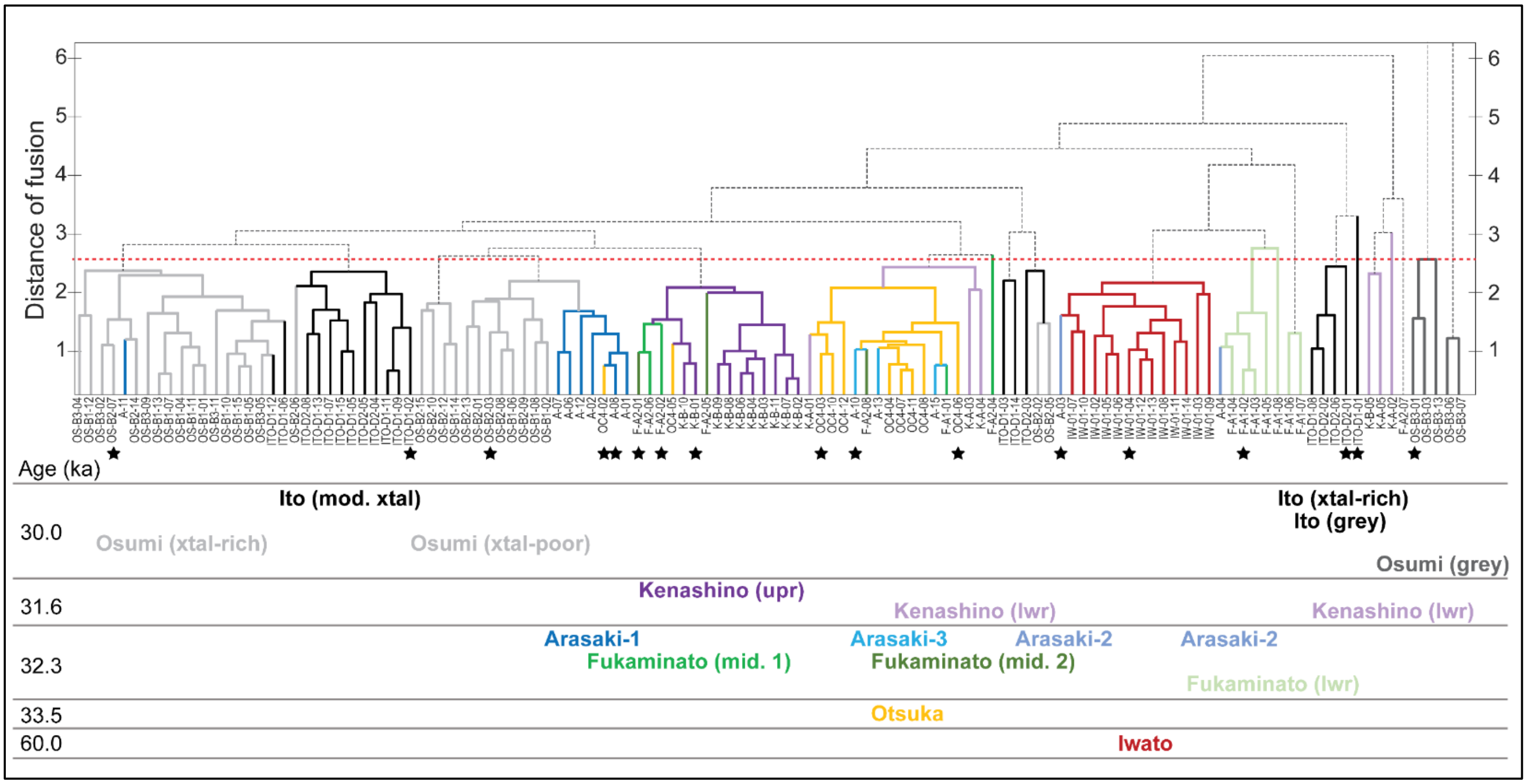

Fig. S2.

Dendrogram depicting the results of our hierarchical clustering analysis. The Euclidean distance at which two samples are clustered together (distance of fusion) is a measure of the similarity between them, such that samples that are more similar are joined at earlier steps with smaller distances of fusion. The red dotted line is the distance cutoff determined using the methodology of Mojena (85) to determine when to stop clustering. Labels of units within each statistically-determined group are shown below the dendrogram, in stratigraphic order, with approximate radiometric eruption ages shown on the left. Black stars denote the pumice clasts that were chosen as representatives from each pumice population for diffusion study.

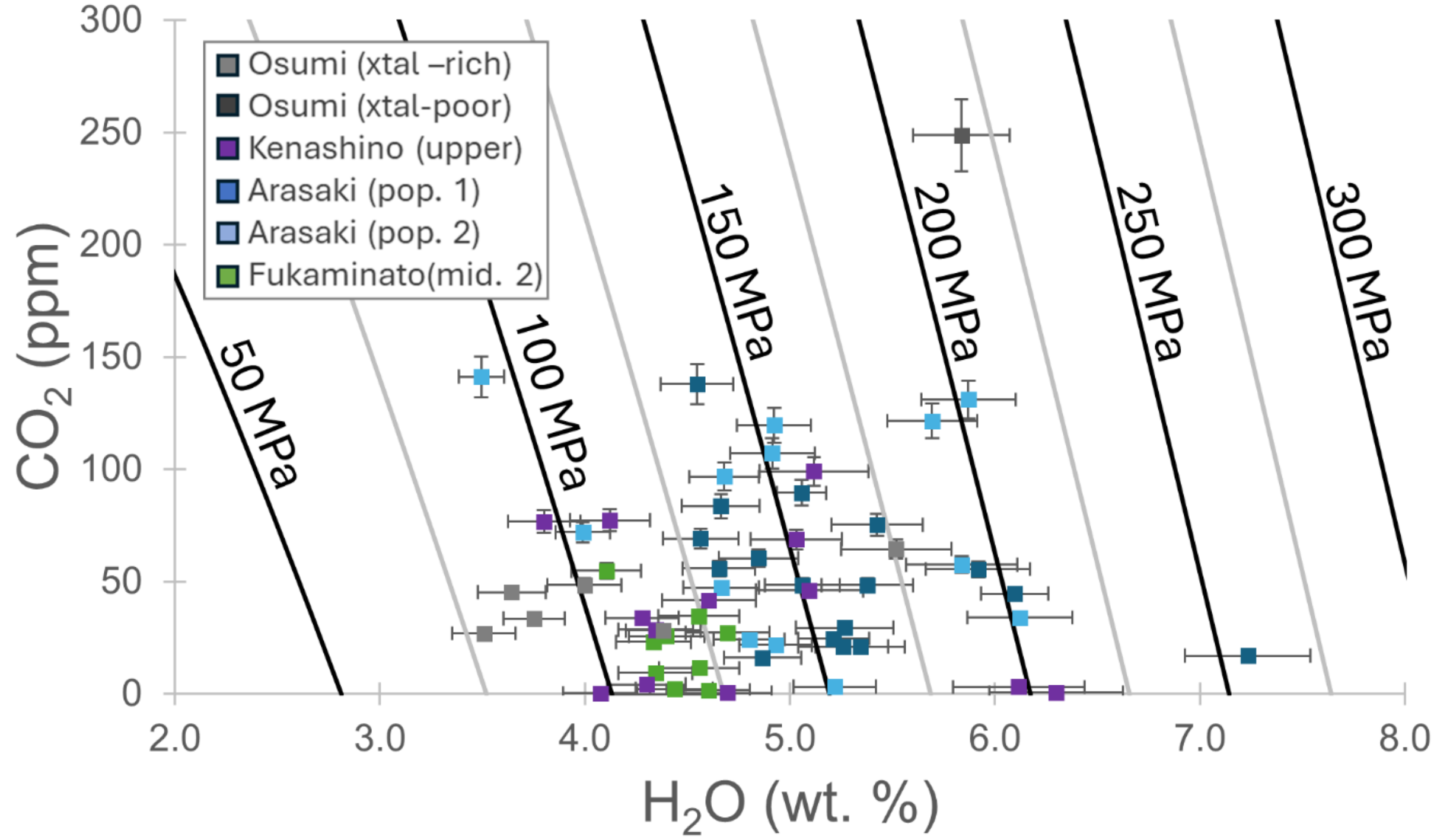

Fig. S3.

Melt inclusion H_2O and CO_2 contents with isobars calculated for rhyolite with VolatileCalc (33). Error bars represent propagated uncertainty in thickness and in the terms of the respective equations (see methods).

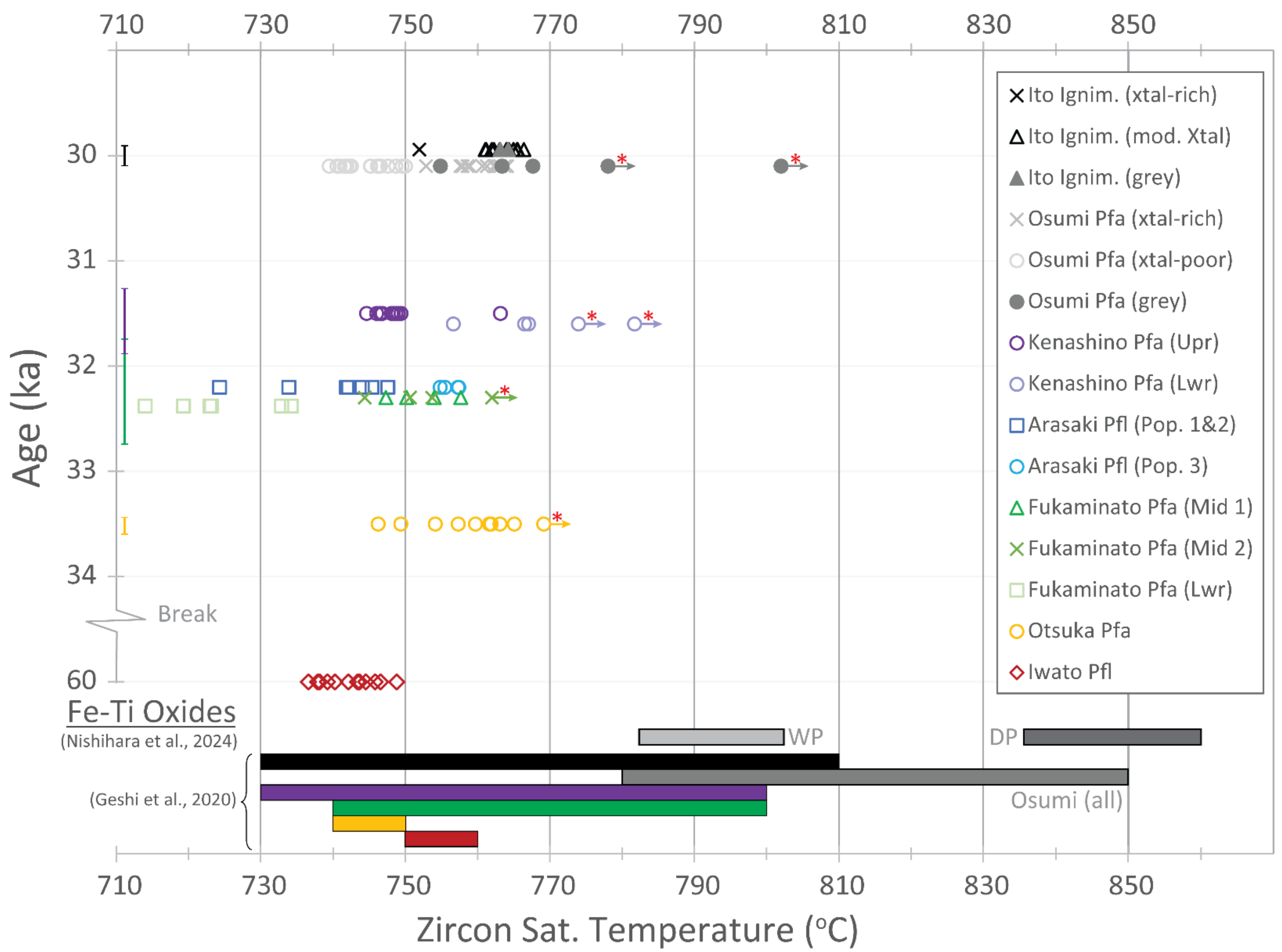

Fig. S4.

Geothermometry results. Mean zircon saturation temperatures for each pumice clast plotted vs. age, calculated using the thermometer of Watson & Harrison (34). Uncertainty in pre-CFE ages are given by thin colored error bars on the left. Glass analyses with higher Zr than whole rock (denoted with red *) may be zircon-undersaturated and would thus be minimum temperatures. Range of Fe-Ti Oxide eruption temperatures from the literature (23, 40) calculated using Ghiorso & Evans (90), are shown below in same colors. Note that while Geshi et al (23) did not distinguish between grey and white pumice from Osumi Pfa, Nishihara et al (40), did provide separate temperature ranges for dark pumice (DP) and white pumice (WP), respectively.

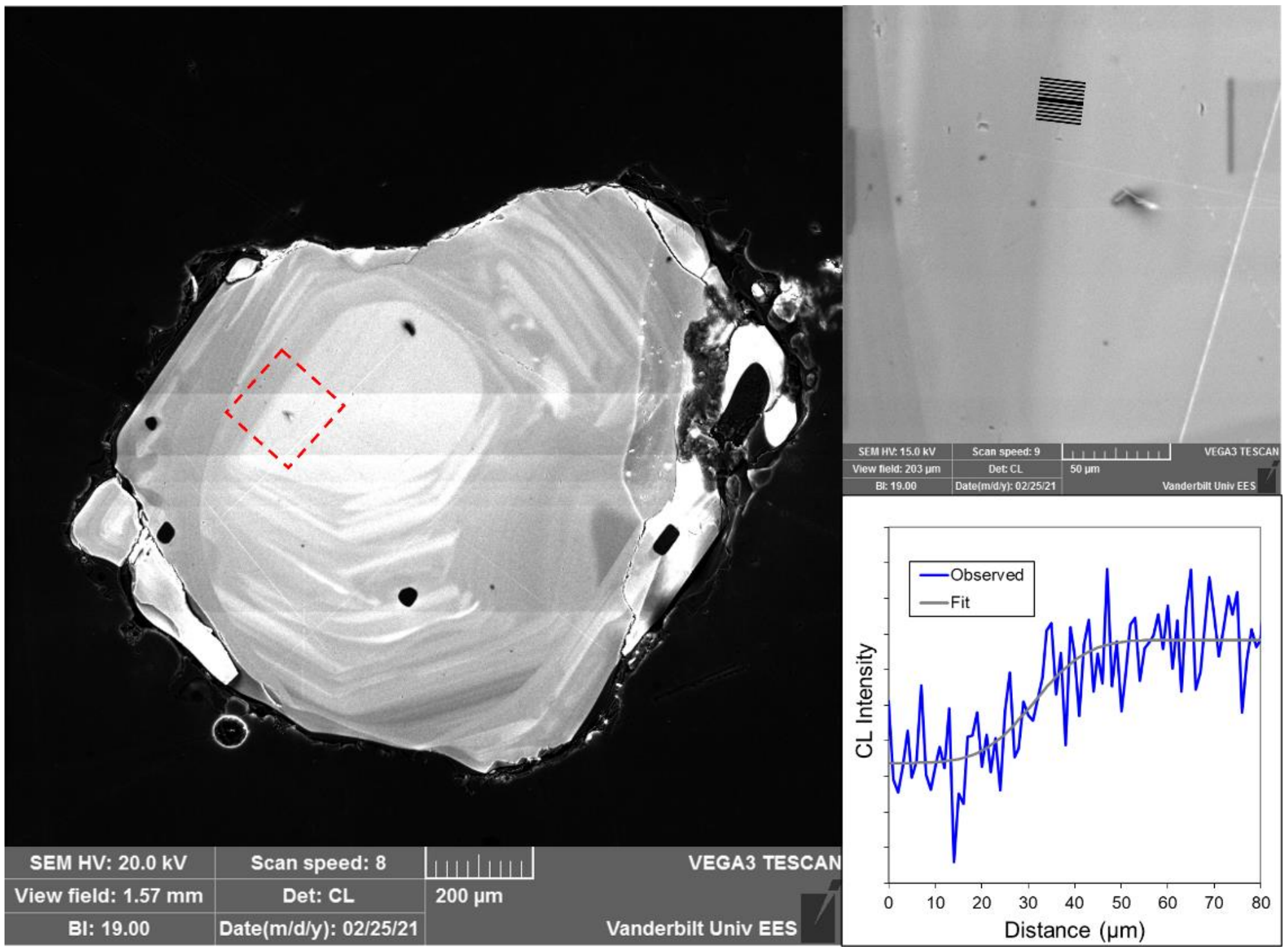

Fig. S5.
Methodology for calculating Ti diffusion timescales for quartz using cathodoluminescence brightness.

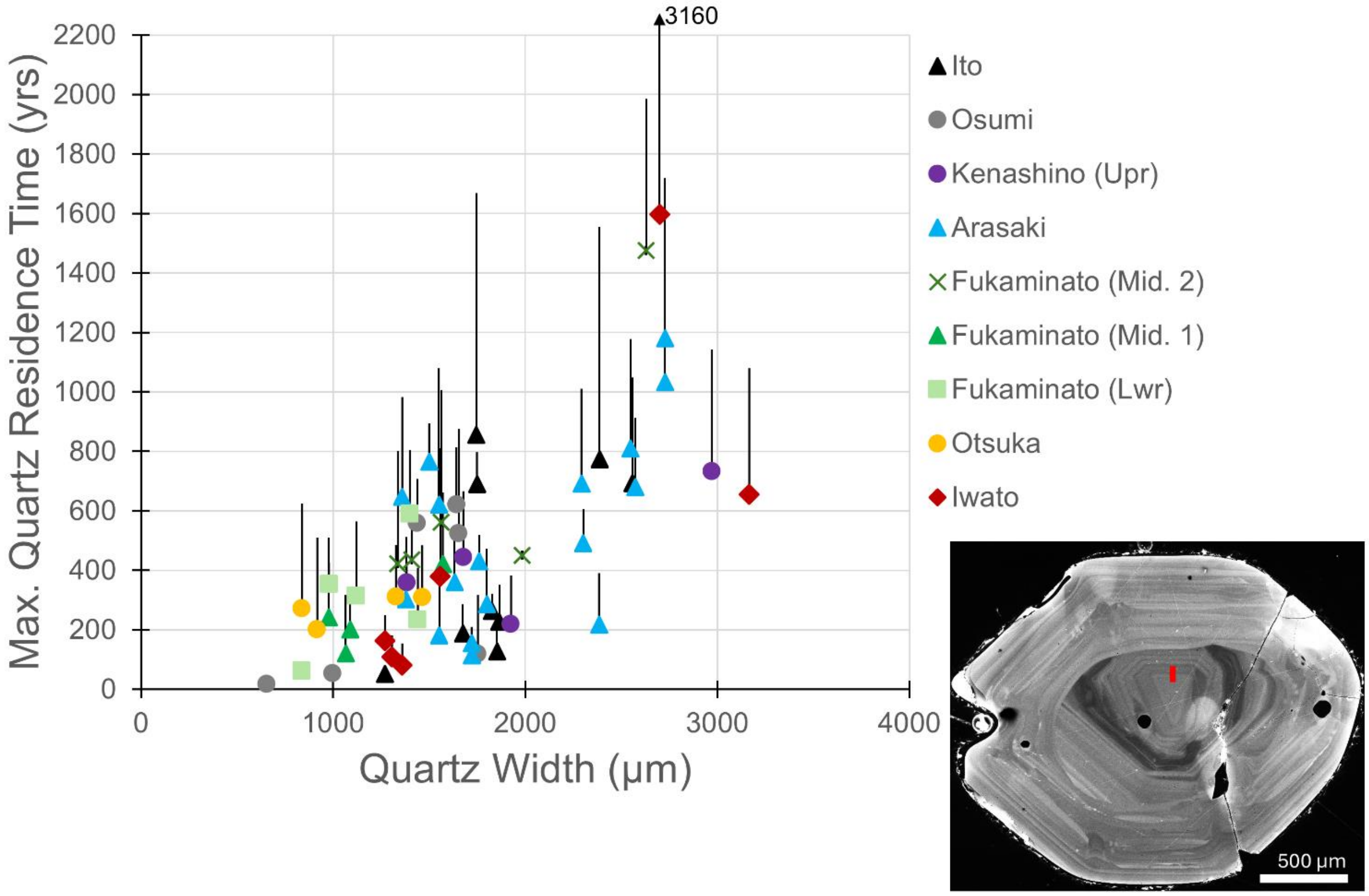

Fig. S6.

Maximum quartz residence times (at 750 °C). These are maximum timescales because of the averaging effect of beam interaction volume, 1D diffusion, sectioning effects, and assumption of an initial step-function. Only diffusion timescales for zone boundaries interior of the first dissolution event (truncation) are shown because they represent the longest diffusion history for each crystal. Symbols represent the median timescale out of the 11 parallel profiles drawn across a quartz zoning boundary; the error bar extend to the maximum timescale from each set of 11 profiles. Example of a cathodoluminescence image of quartz grain is shown – note that the modeled zone boundary (red line) is interior of the first resorption (truncation). This is the same image as shown in Fig. 5 in the main text and the data are the same as the circles in that figure.

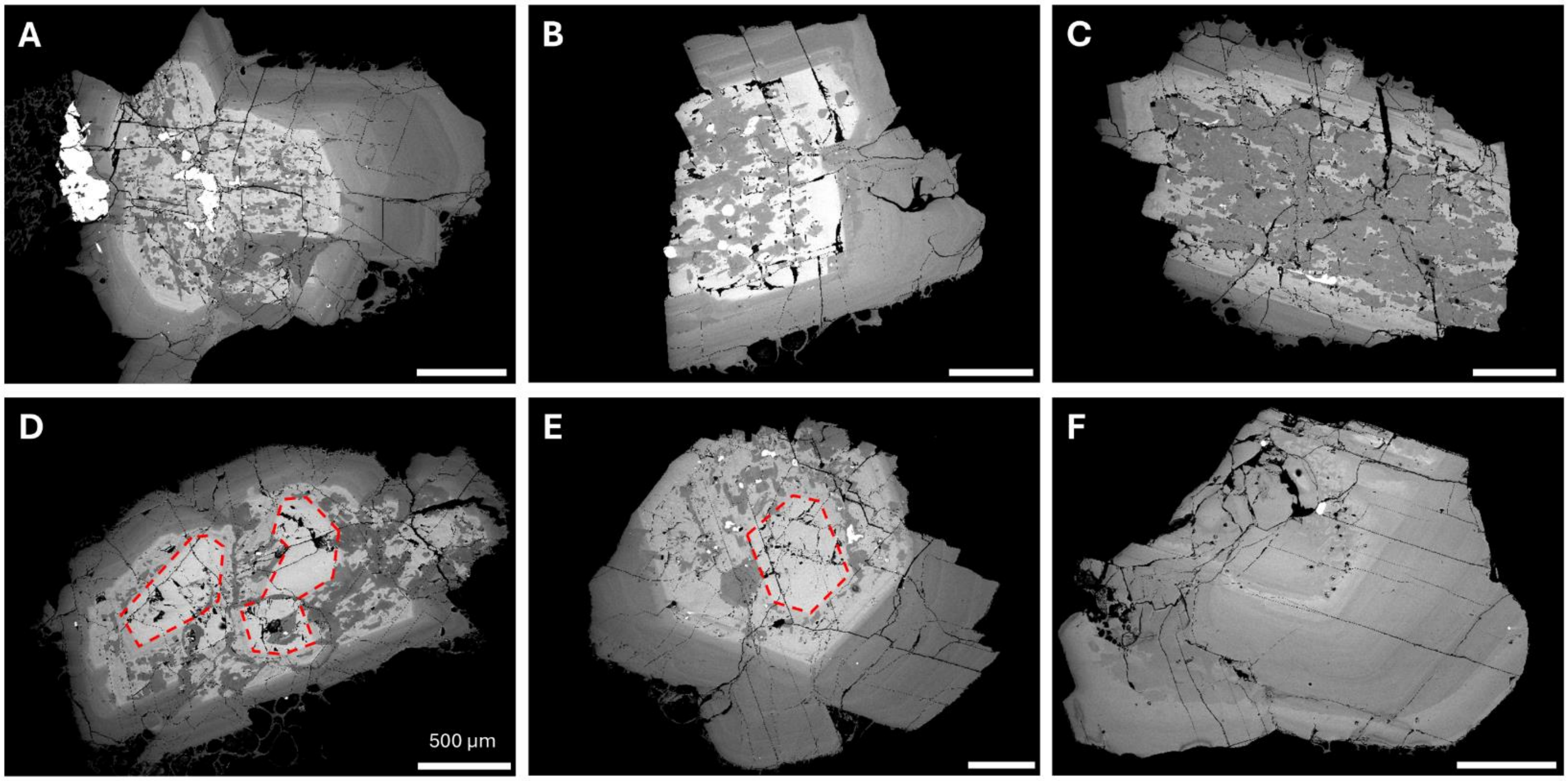

Fig. S7.

Backscatter electron images of plagioclase from pre-AT and AT units. Brighter indicates higher Anorthite content (An). Many plagioclase crystals contain an An40 patchy core, mantled by a An75-90 zone, with a sharp euhedral decrease to An40 interior (A – C). Some rare plagioclase crystals contain a euhedral, monotonous An70-90 core-most zone interior of the patchy zone (red outlined regions in D & E). Most rims tend to be monotonous ~An40 or contain low amplitude oscillatory zoning, with changes of less than An5 (B, C, E). However, some have a resorption event recorded in rim with an anorthite increase of 5 – 10 (F). Images B and D are also shown in Figure 7 in the main text.

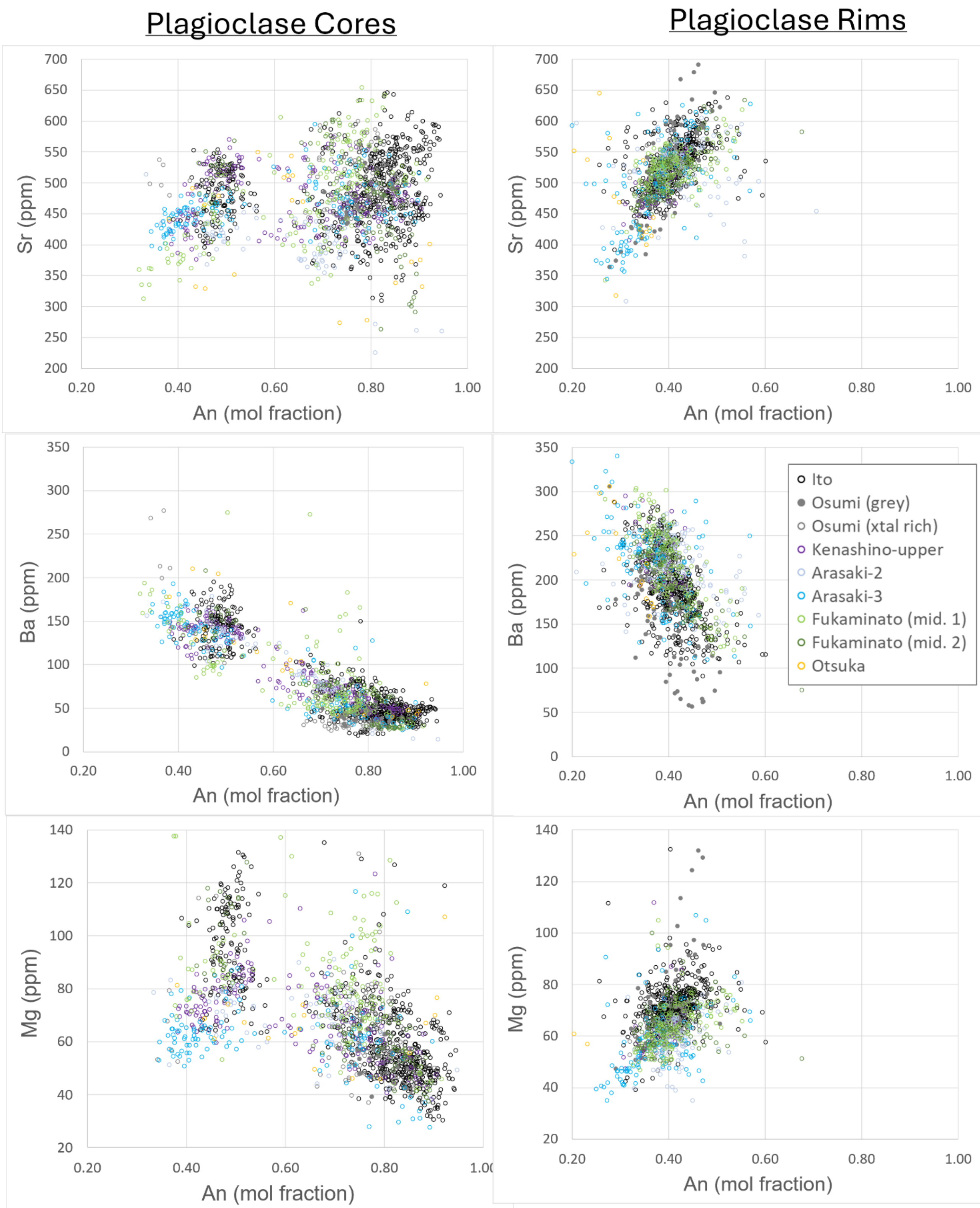

Fig. S8.

Plagioclase Sr, Ba, and Mg vs. Anorthite content for cores (left) and rims (right). Note that the analyses of interior regions of plagioclase are not shown in any plots.

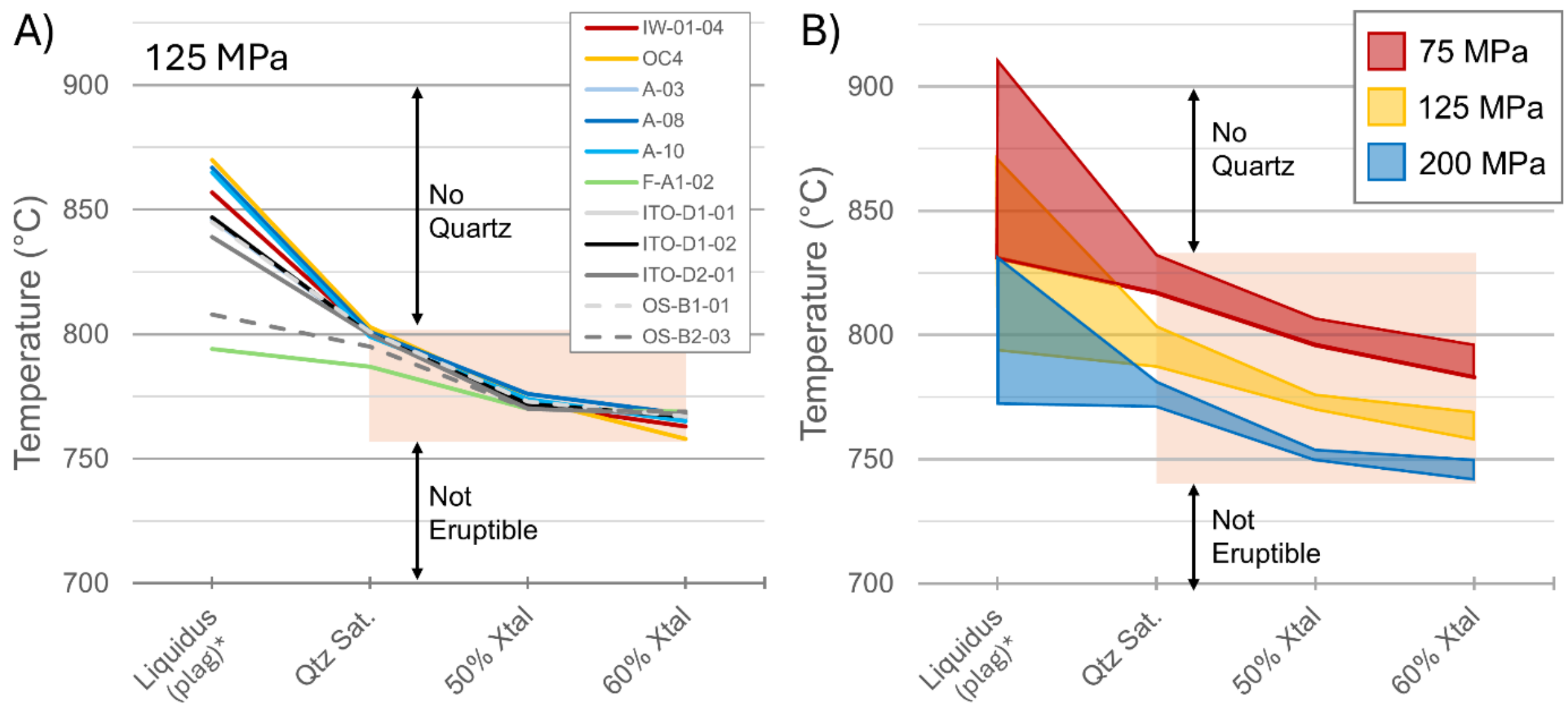

Fig. S9.

Evolution of Aira magmas during crystallization as determined by rhyolite-MELTS modelling. Each line in (A) shows the temperatures at which magma first begins crystallizing (liquidus)*, quartz saturates, and the magma reaches 50% and 60% crystals for all whole rock compositions in this study, modelled at 125 MPa. The pink box represents the maximum temperature range for which quartz is saturated and the magma is eruptible (< 60 % crystals). These lines are represented in (B) as the yellow field, along with red and blue fields denoting the range of models at 75 and 200 MPa, respectively (pressure range determined by the rhyolite-MELTS geobarometer). *Plagioclase is the first phase to crystallize in all model runs except three AT samples at 200 MPa, wherein plagioclase saturates 3-6°C lower than spinel.

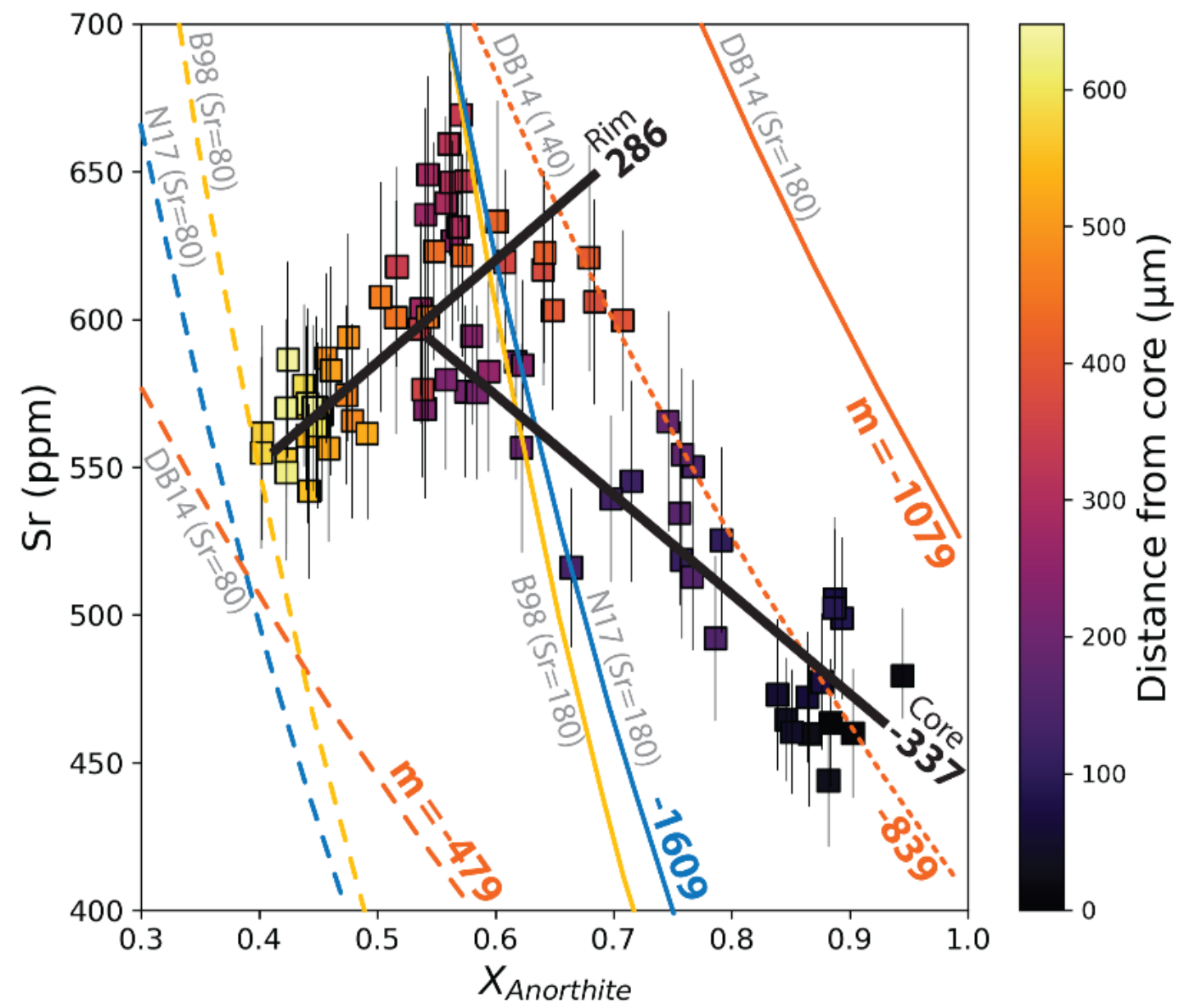

Fig. S10.

Example comparing observed Sr vs. An from one plagioclase grain to several modelled equilibrium slopes. Plagioclase Sr vs. An data observed in ITO-D1-02_Plag-12 core and rim (the same as shown in Fig. 7D), but compared to the equilibrium compositions calculated using three different partitioning relationships (DB14 = Dohmen & Blundy (46); Nielsen et al (47); B98 = Bindeman et al (45)). These are the slopes expected if plagioclase reached full equilibrium with a magma with 80 ppm Sr (average glass Sr) or 180 ppm Sr (average whole-rock Sr), (dashed and solid lines, respectively). The equilibrium compositions with an intermediate Sr = 140 ppm, using DB14, is also shown for comparison. The solid black lines are linear regressions for the core and rim portions of this plagioclase example. Bold colored values give the slope of each line. Note that the core is not as steeply negative as any of the equilibrium lines. The slopes of these lines are compared to all plagioclase cores and rims on Figure 7E (with the same color scheme).

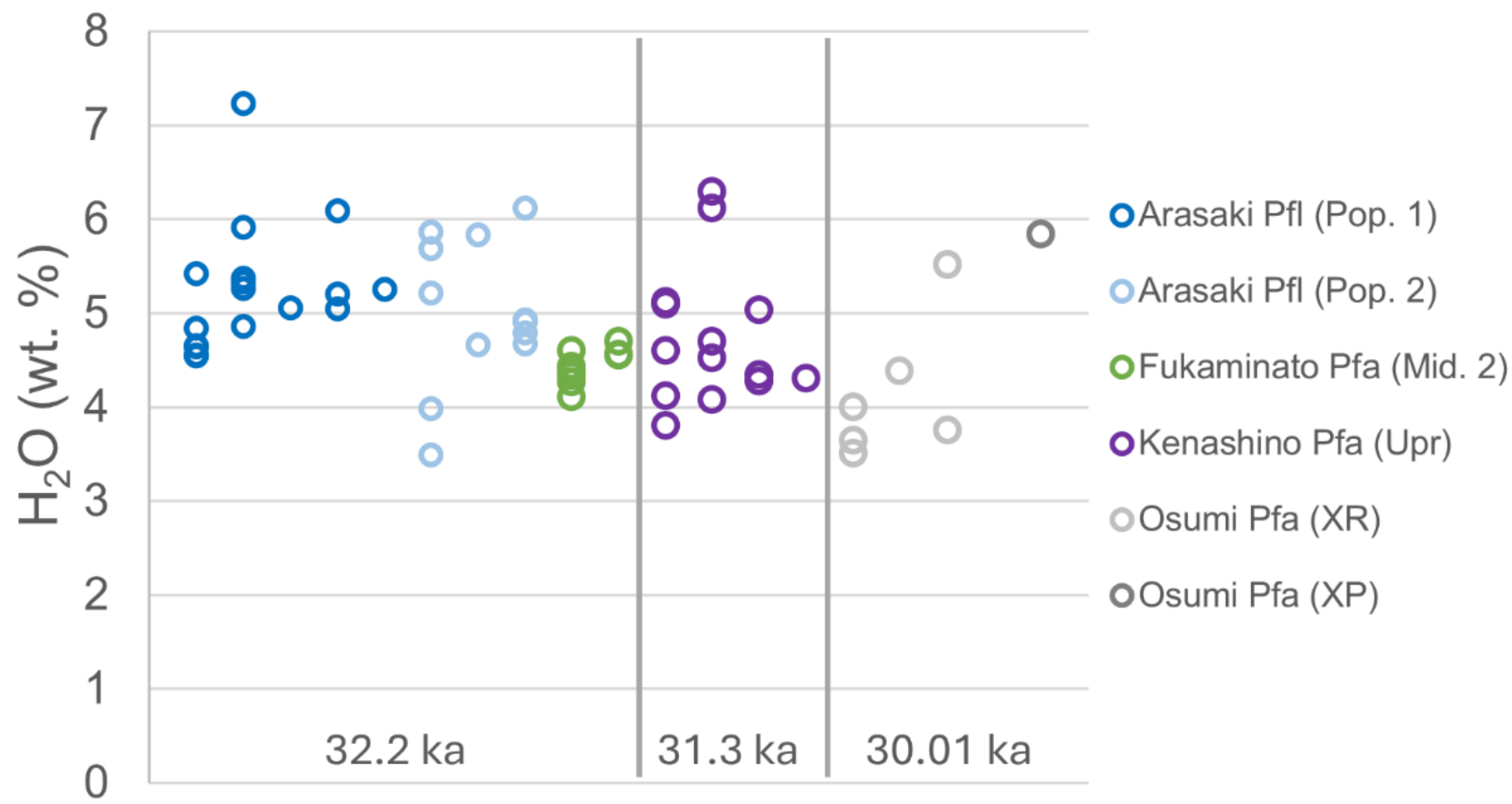

Fig. S11.

Melt inclusion H_2O contents determined by FTIR. Concentrations are shown such that each vertical line represents melt inclusions from a single quartz host. XR = Osumi crystal-rich, XP = Osumi crystal poor.

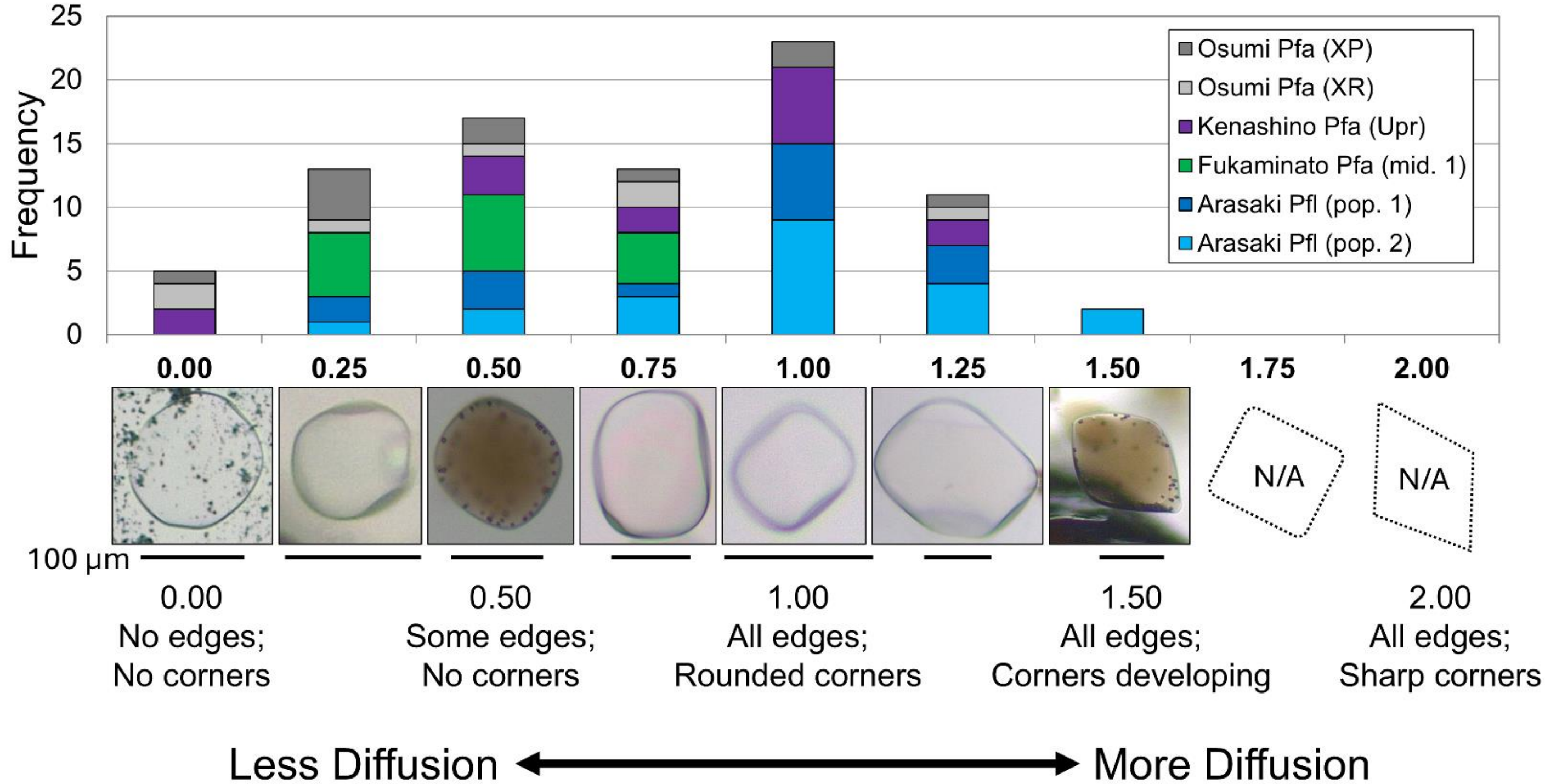

Fig. S12.

Faceting of melt inclusions. Histogram showing the faceting index (62) of quartz-hosted melt inclusions observed within six Aira samples (N=84 melt inclusions, within 21 quartz crystals). Examples of Aira melt inclusions are given beneath each faceting index and the descriptions are directly from Boro et al (62). Scale bars beneath images represent 100 µm. No highly faceted melt inclusions (index = 1.75 – 2) were observed in Aira quartz.

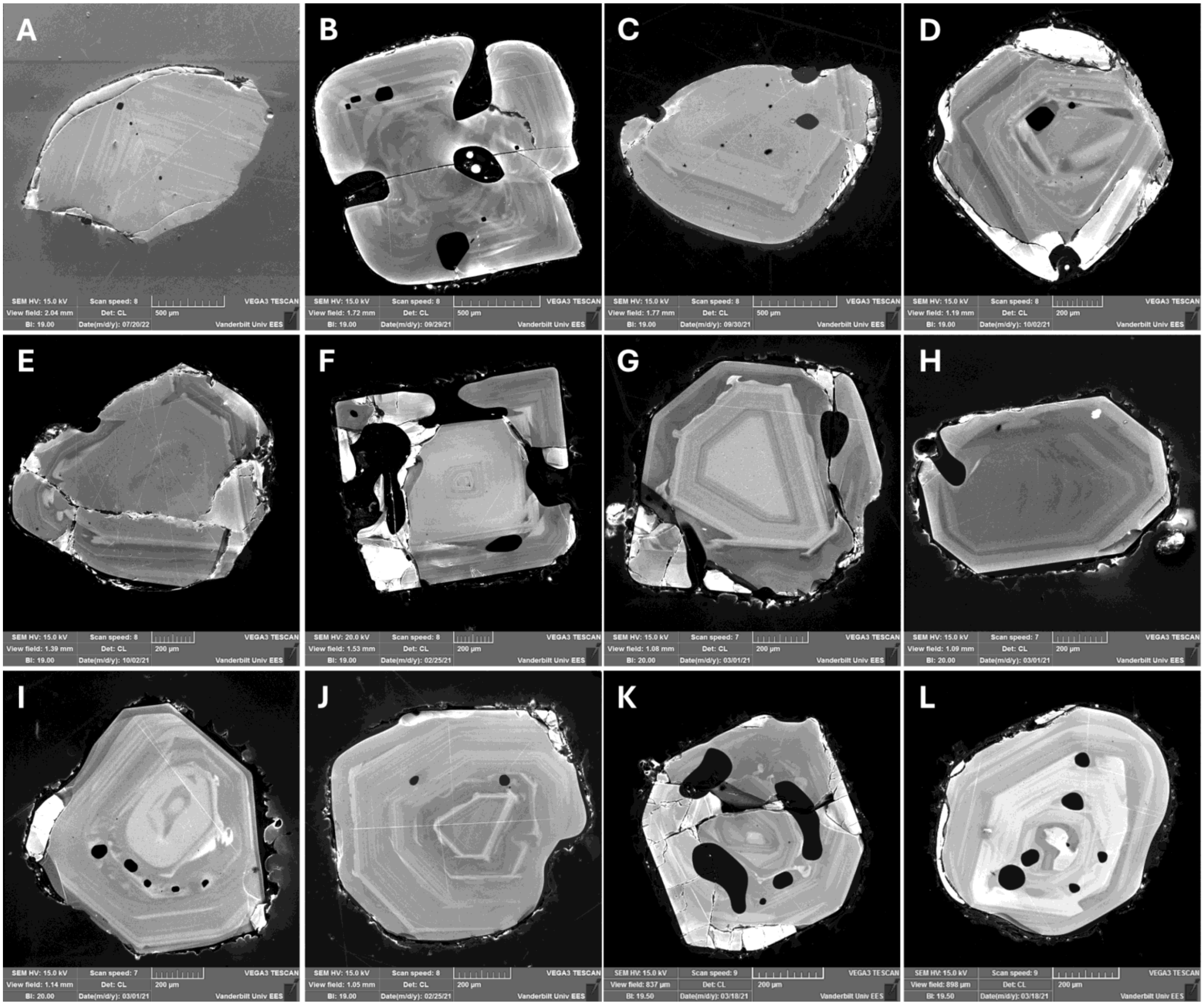

Fig. S13.

Cathodoluminescence images of quartz crystals that exhibit skeletal growth textures of various morphologies. Examples shown are from Osumi (crystal-rich) (A), Arasaki-3 (B), Otsuka (C, D, E), Fukaminato-lower (G, H, I), and Kenashino (J, K, L). Images B and F are also shown in Fig. 4 in the main text.

Table S1.

Radiocarbon Ages. Details are given in Supplementary Data File S7.

Tephra	Sampling Location	δ13C (‰) (AMS)	Calibrated Age 2σ range (yrBP)	Average Age (yrBP)
Kenashino Pfa	31.773586°N, 130.77387°E	-19.55 ± 0.21	31298 - 30921	31110
Fukaminato Pfa	31.554631°N, 130.740021°E	-20.33 ± 0.24	32755 - 31743	32249
Otsuka Pfa	31.773586°N, 130.77387°E	-20.84 ± 0.20	33795 - 33104	33450

Supplementary Data Files:

Data S1. (separate file)

Major and trace element analyses for pumice glass and whole-pumice, determined by SEM-EDS, LA-ICP-MS, and XRF.

Sheet 1: Pumice Glass major and trace element analyses
Sheet 2: Plagioclase major and trace element analyses
Sheet 3: SEM-EDS secondary standards
Sheet 4: LA-ICP-MS secondary standards
Sheet 5: Whole pumice XRF data
Sheet 6: XRF standard analyses

Data S2. (separate file)

Geobarometry results.

Sheet 1: Rhyolite Melts equilibration pressures.
Sheet 2: Melt inclusion H_2O and CO_2 and geobarometry results

Data S3. (separate file)

Zircon saturation temperatures, using the average pumice clast Zr (by LA-ICP-MS) and major element (by SEM-EDS). Temperatures were calculated using the thermometers of Watson and Harrison (34) ("BH83"), Boehnke et al., (35) ("B13"), and Gervasoni et al. (36) ("G16").

Data S4. (separate file)

Quartz diffusion results. Diffusion timescales are given for 750 °C, using C07, A23, J20, and A21.

Data S5. (separate file)

Plagioclase diffusion results.

Sheet 1: Plagioclase LA-ICP-MS results
Sheet 2: Slope and R of profiles
Sheet 3: Forward modelling results
Sheet 4: Successful FD models

Data S6. (separate file)

Compilation of literature zircon ages.

Sheet 1: Total residence time of magma (oldest U-Pb, U-Th, Rb-Sr age minus eruption age)
Sheet 2: Zircon crystallization ranges (max zircon U-Pb age minus min zircon U-Pb age, compilation from Curry et al. (97))

Data S7. (separate file)

Pre-AT tephra 14C age determinations.

Data S8.

Plots showing the observed Sr and An vs. distance and Sr vs. An for all plagioclase profiles used to calculate Sr/An slopes in Figure 7. The equilibrium profile and slope using the partition coefficient of Nielsen et al. (47) is also shown.

ITO-D1-01_Plag-1_CORE-2

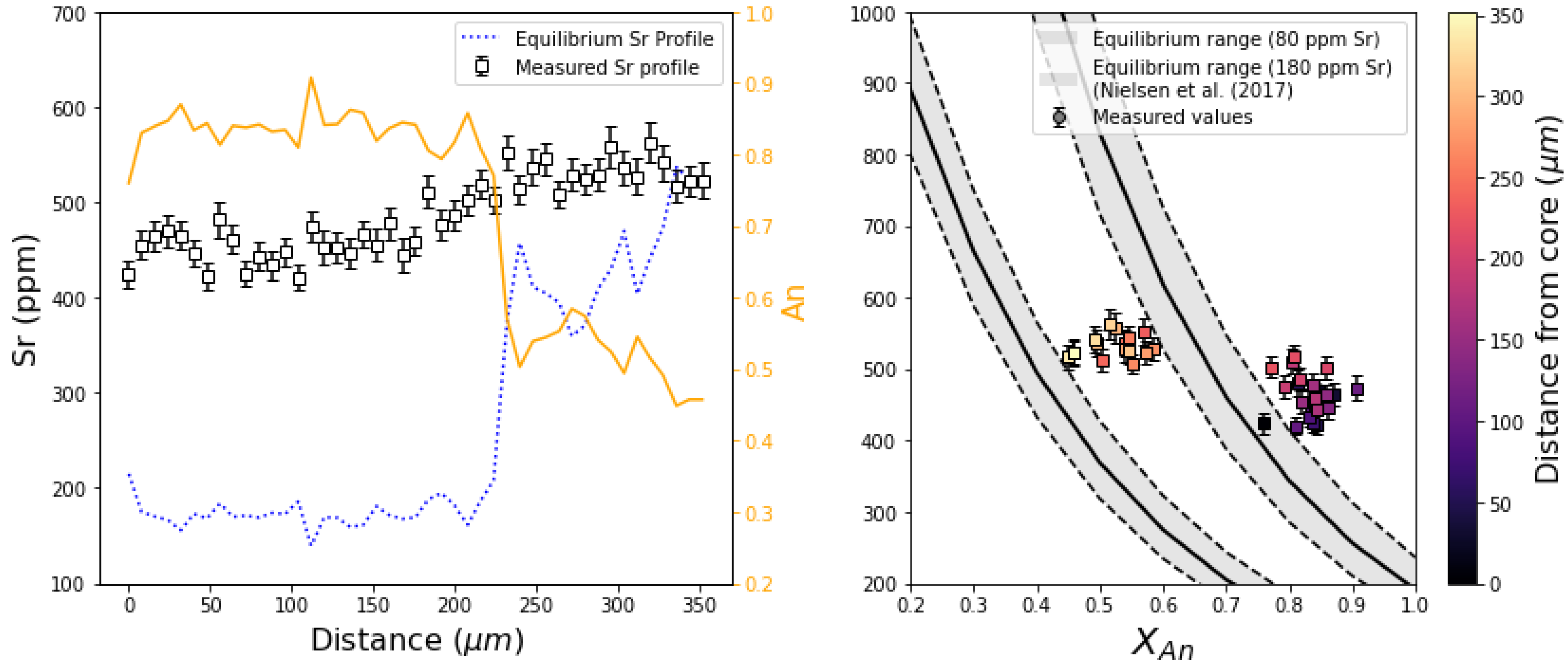

ITO-D1-01_Plag-1_CORE-1

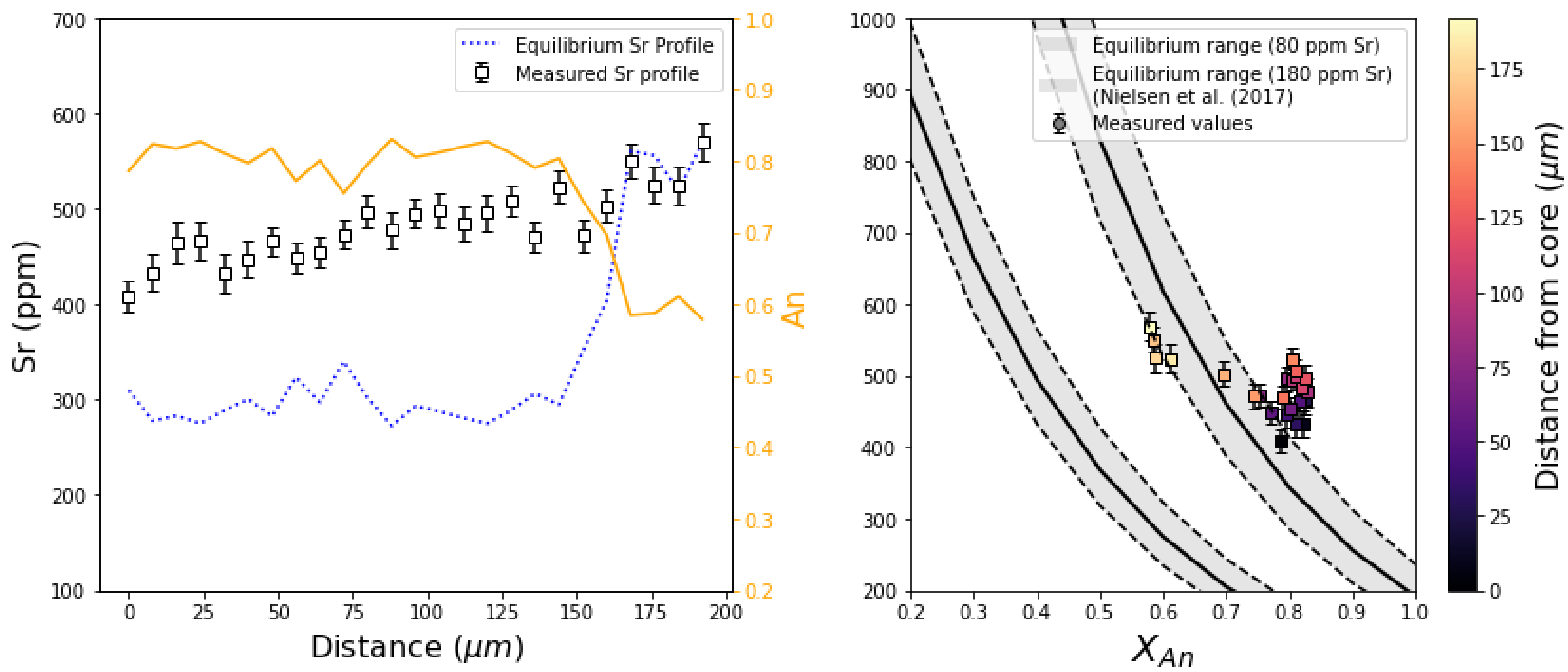

ITO-D1-01_Plag-2_CORE-2

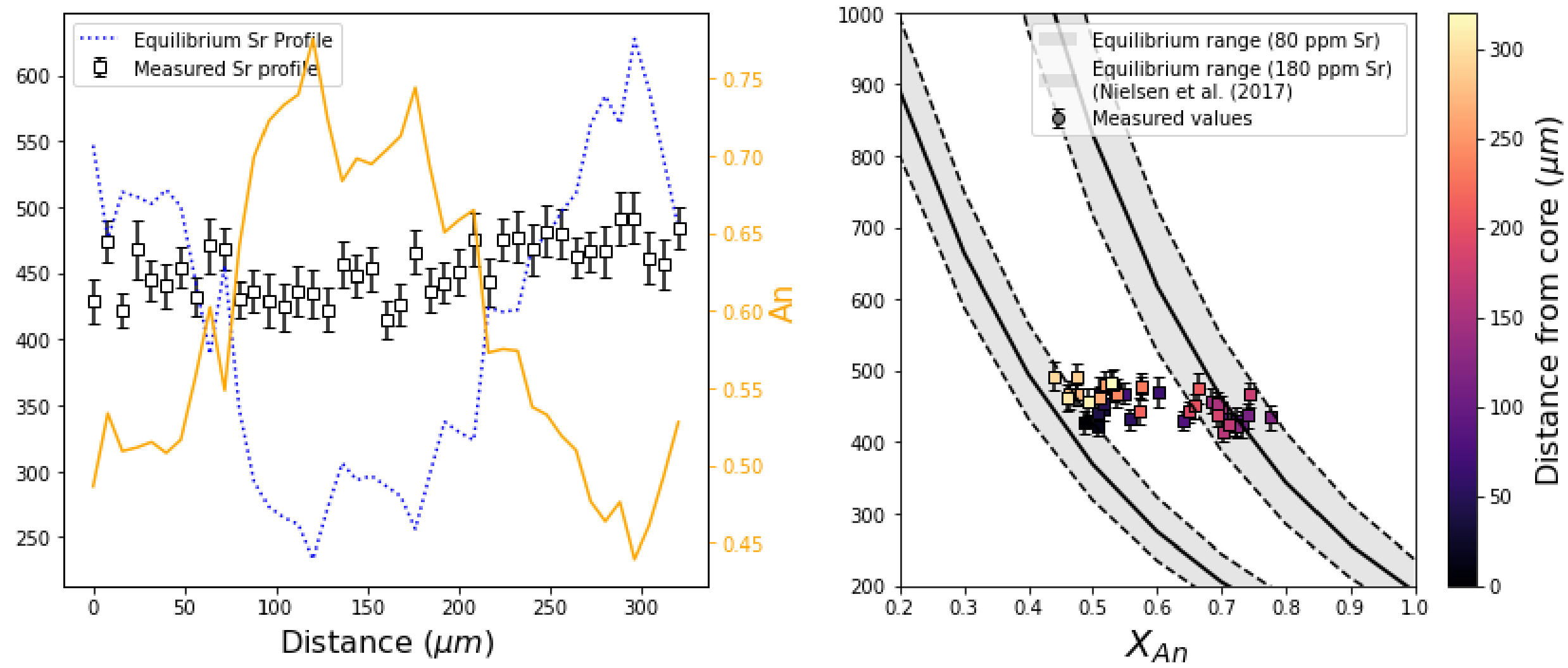

ITO-D1-01_Plag-11_CORE-1

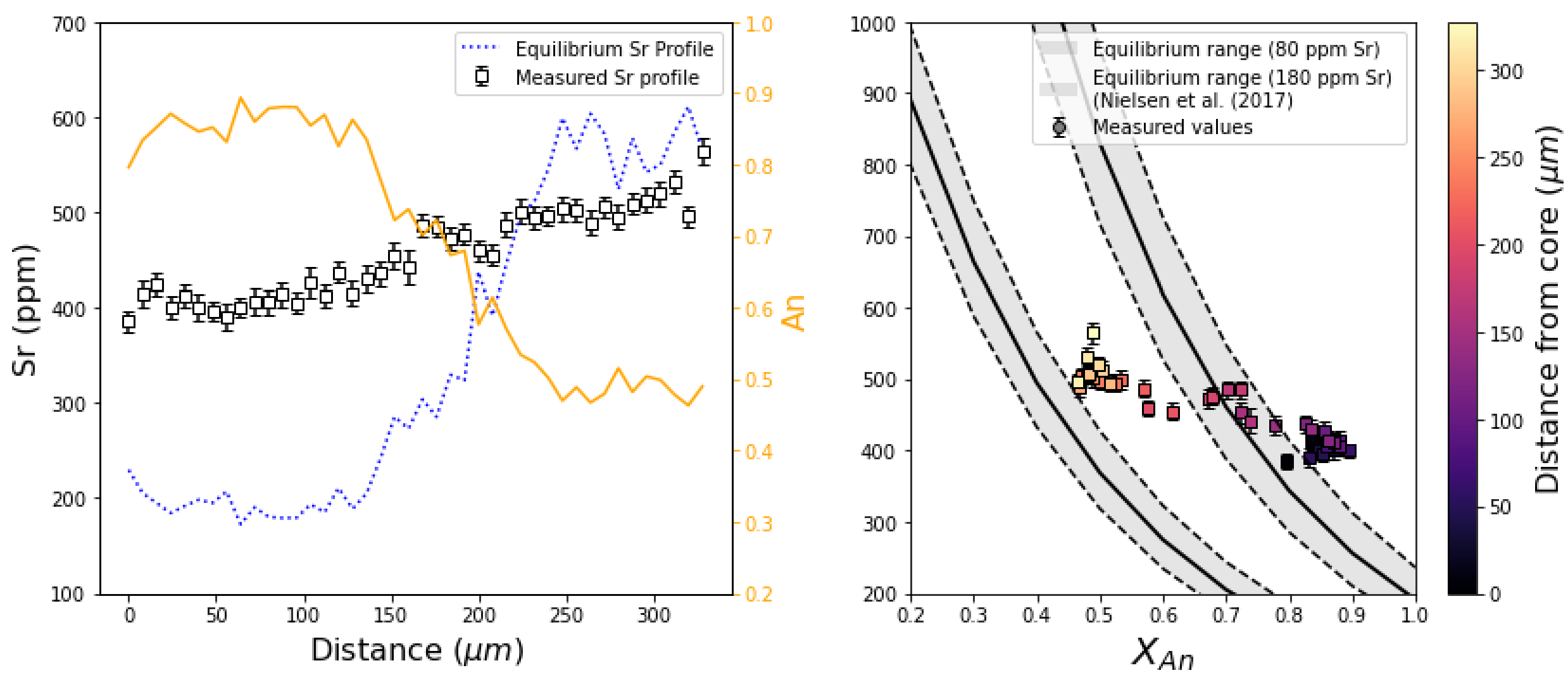

ITO-D1-02_Plag-4_CORE-1

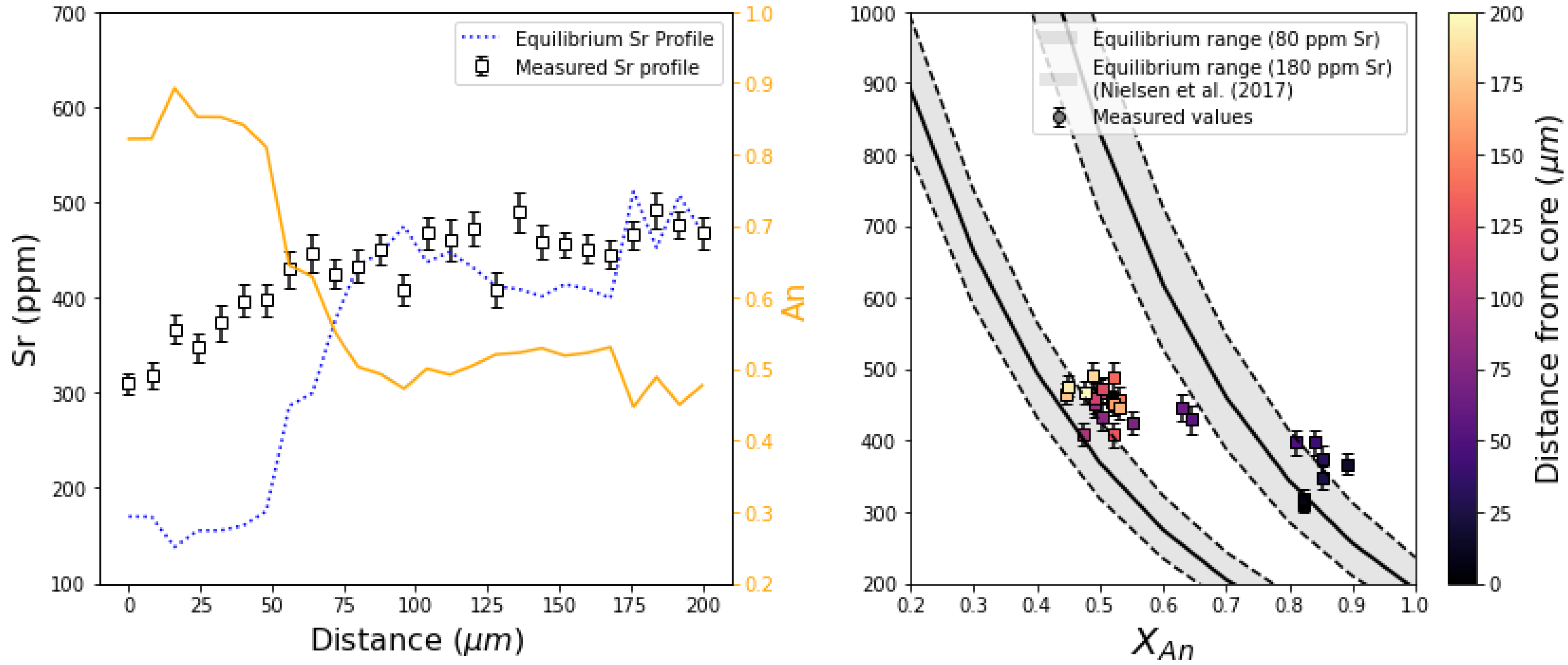

ITO-D1-02_Plag-12_CORE-1

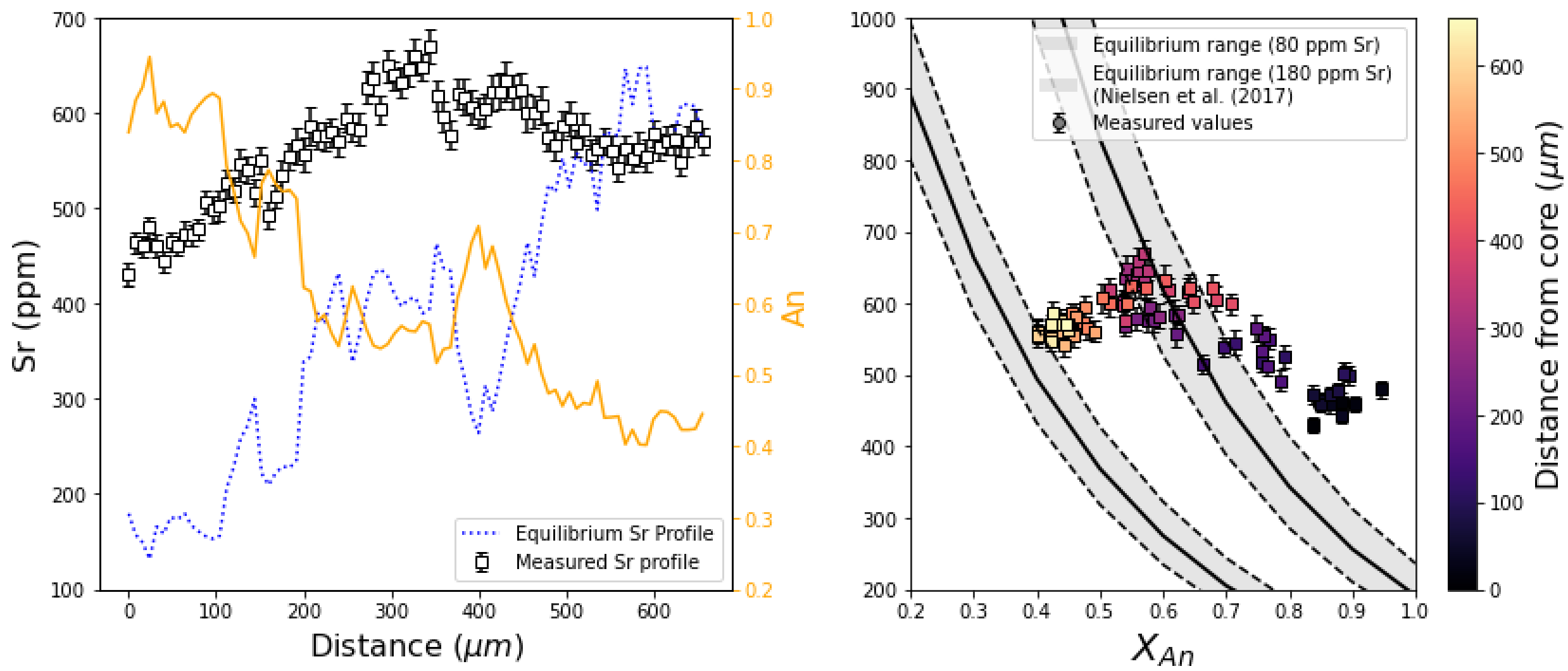

ITO-D2-01_Plag-7_CORE-3

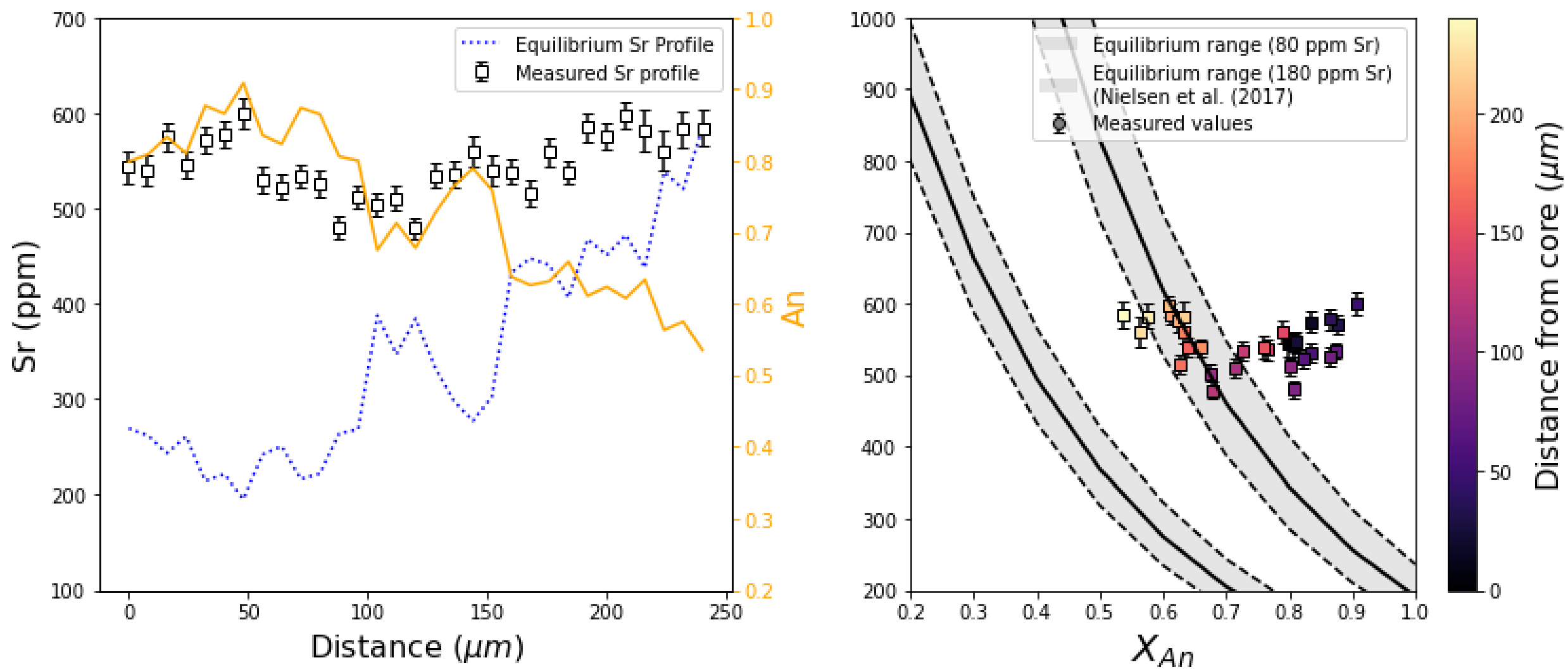

ITO-D2-01_Plag-8_CORE-1

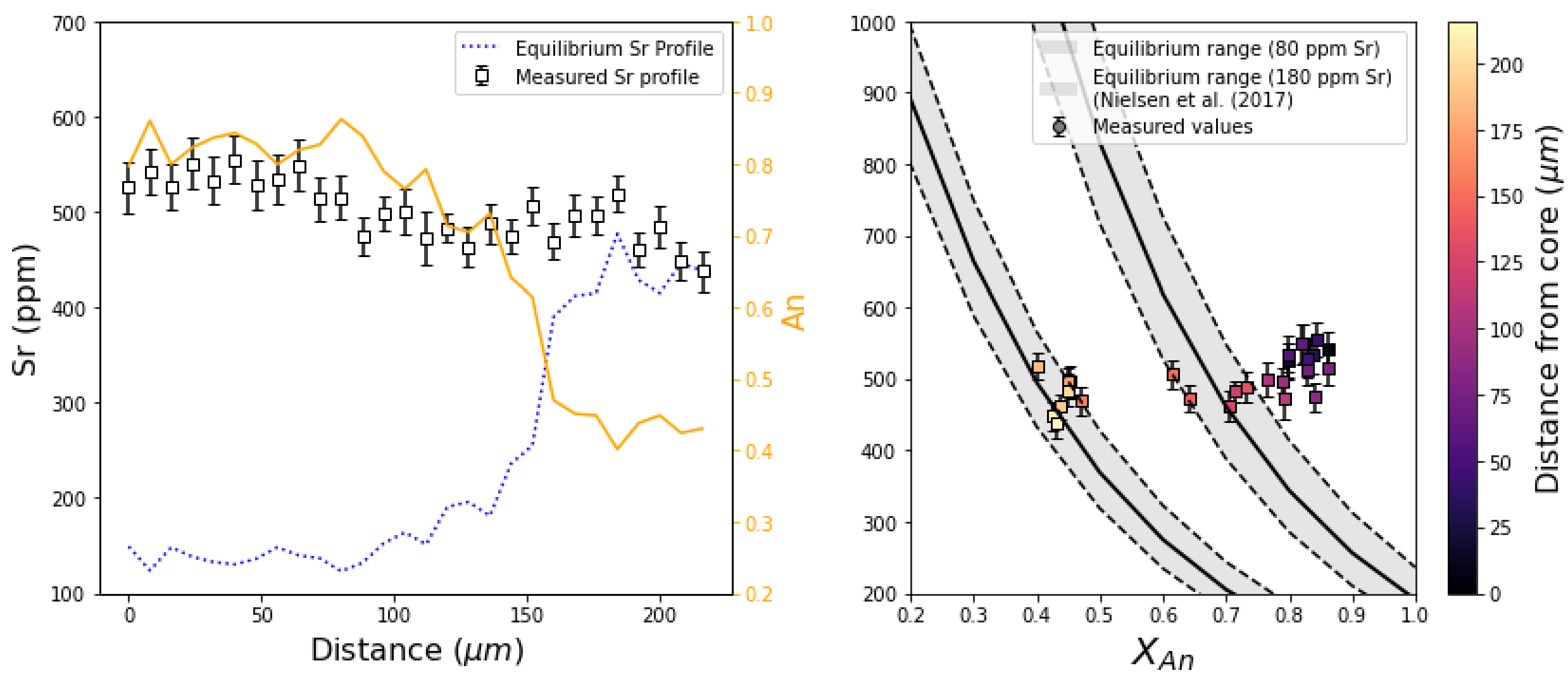

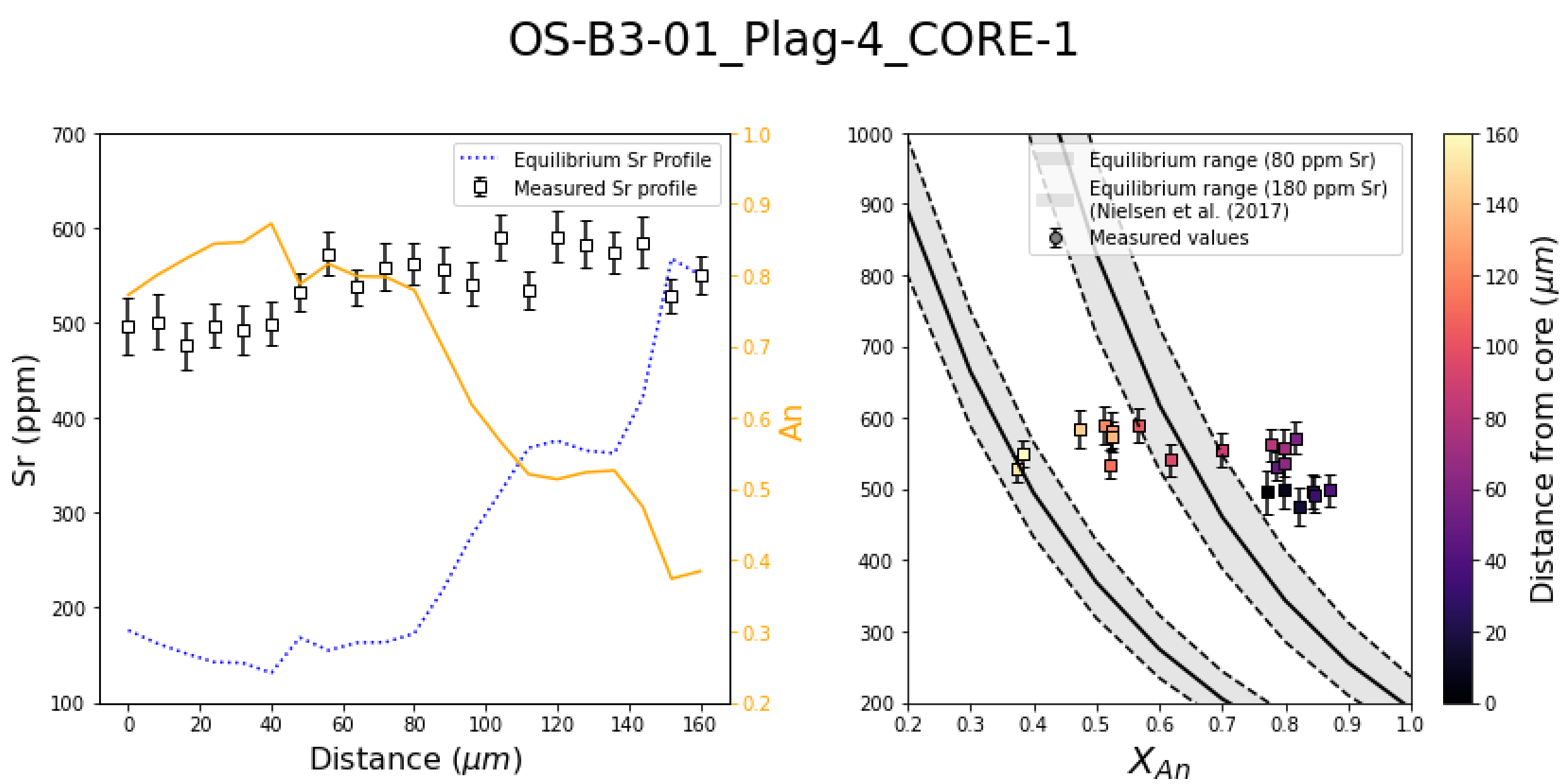

OS-B3-01_Plag-4_CORE-1
Equilibrium Sr Profile
Measured Sr profile
Sr (ppm)
An
Distance (μm)
Equilibrium range (80 ppm Sr)
Equilibrium range (180 ppm Sr)
(Nielsen et al. (2017)
Measured values
X_{An}
Distance from core (μm)

OS-B1-01_Plag-1_CORE-2

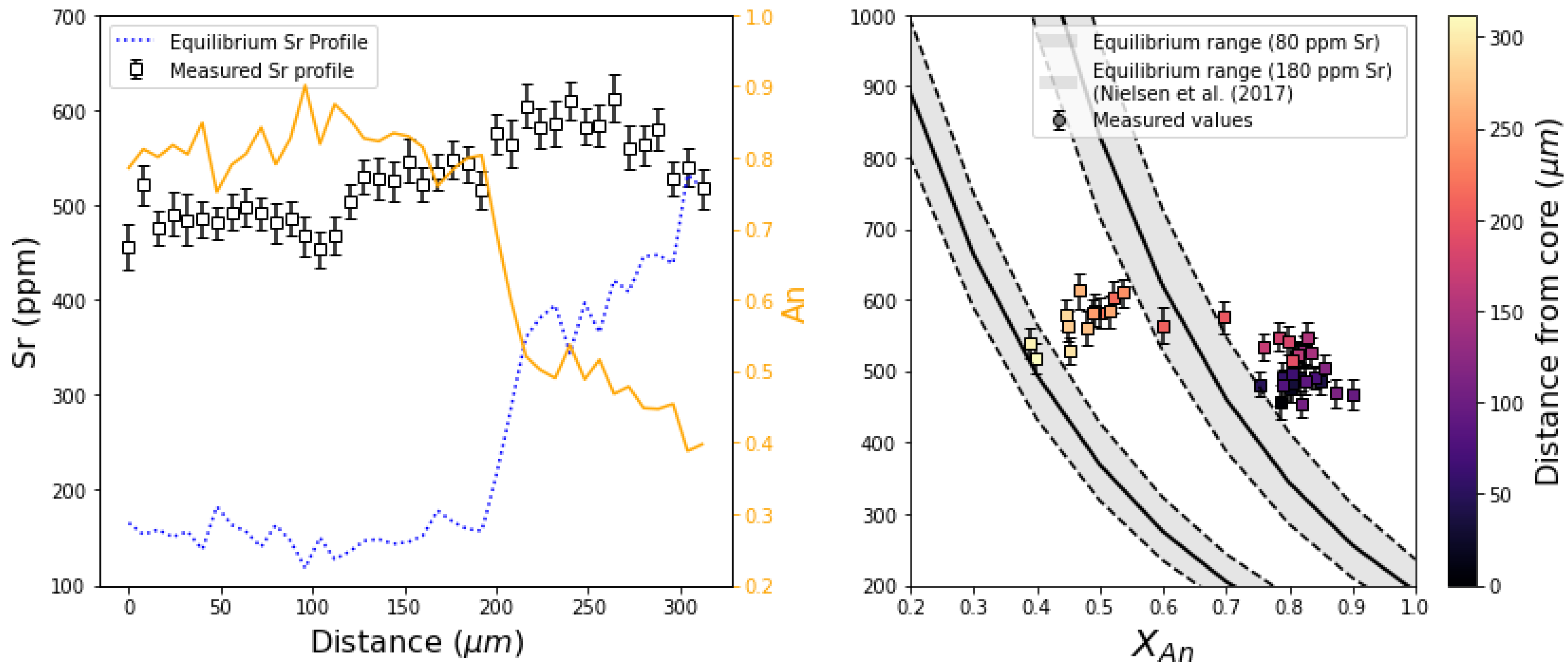

OS-B1-01_Plag-2_CORE-1

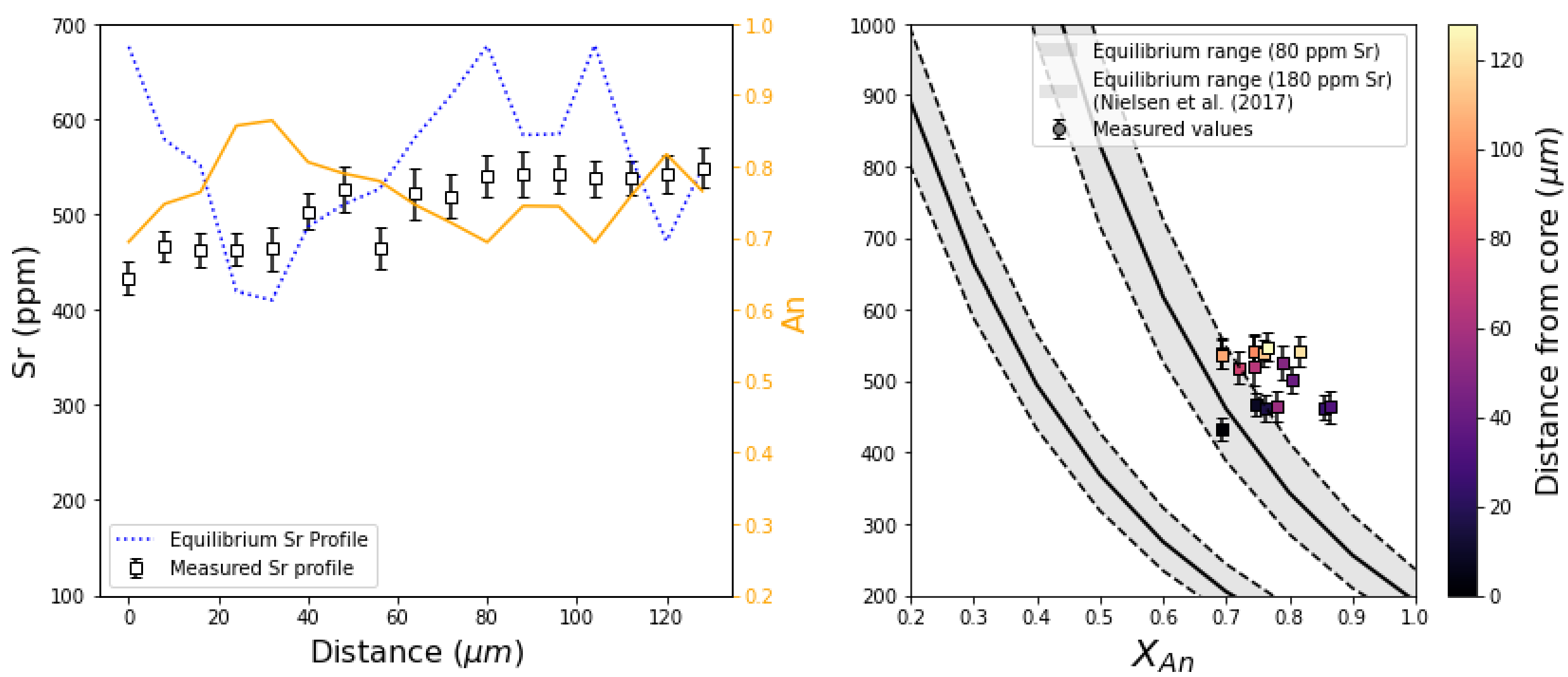

OS-B1-01_Plag-2_CORE-2

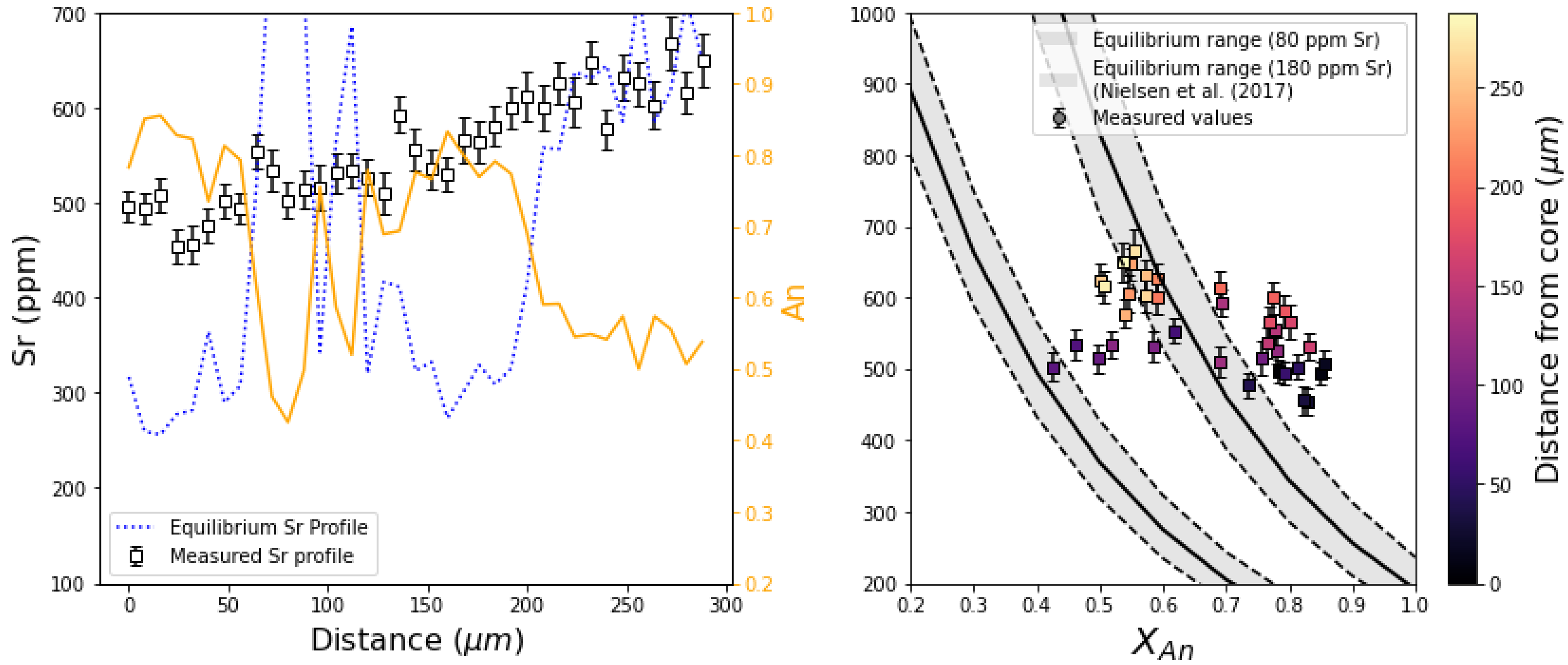

OS-B1-01_Plag-2_CORE-3

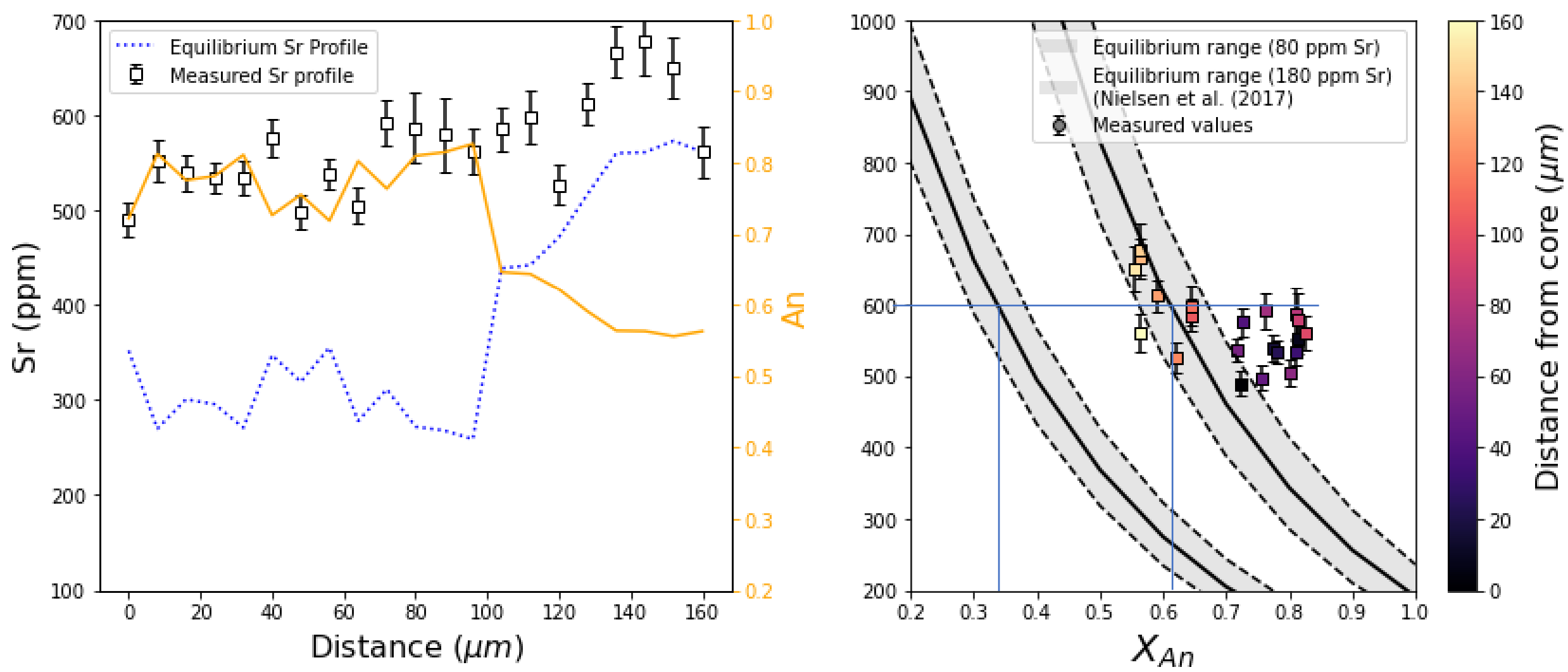

K-B-01_Plag-5_CORE-1

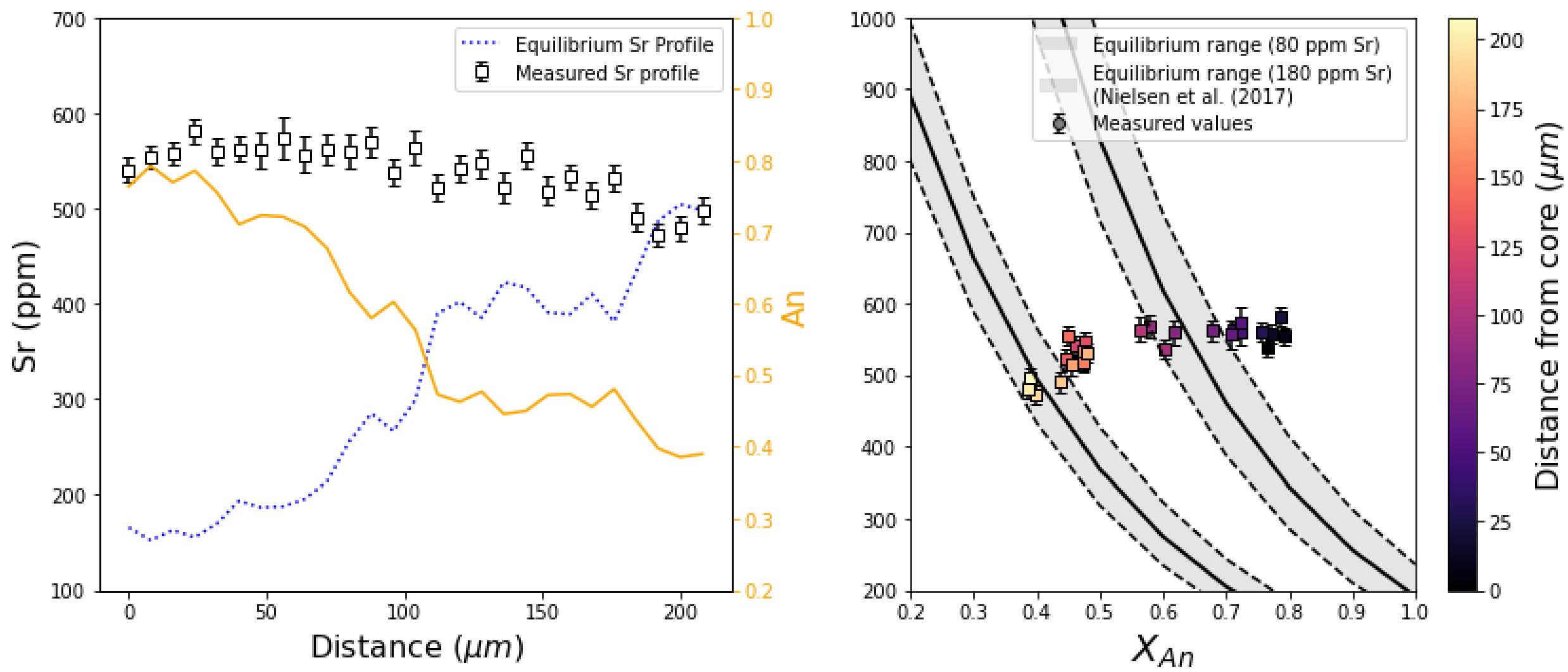

K-B-01_Plag-7_CORE-1

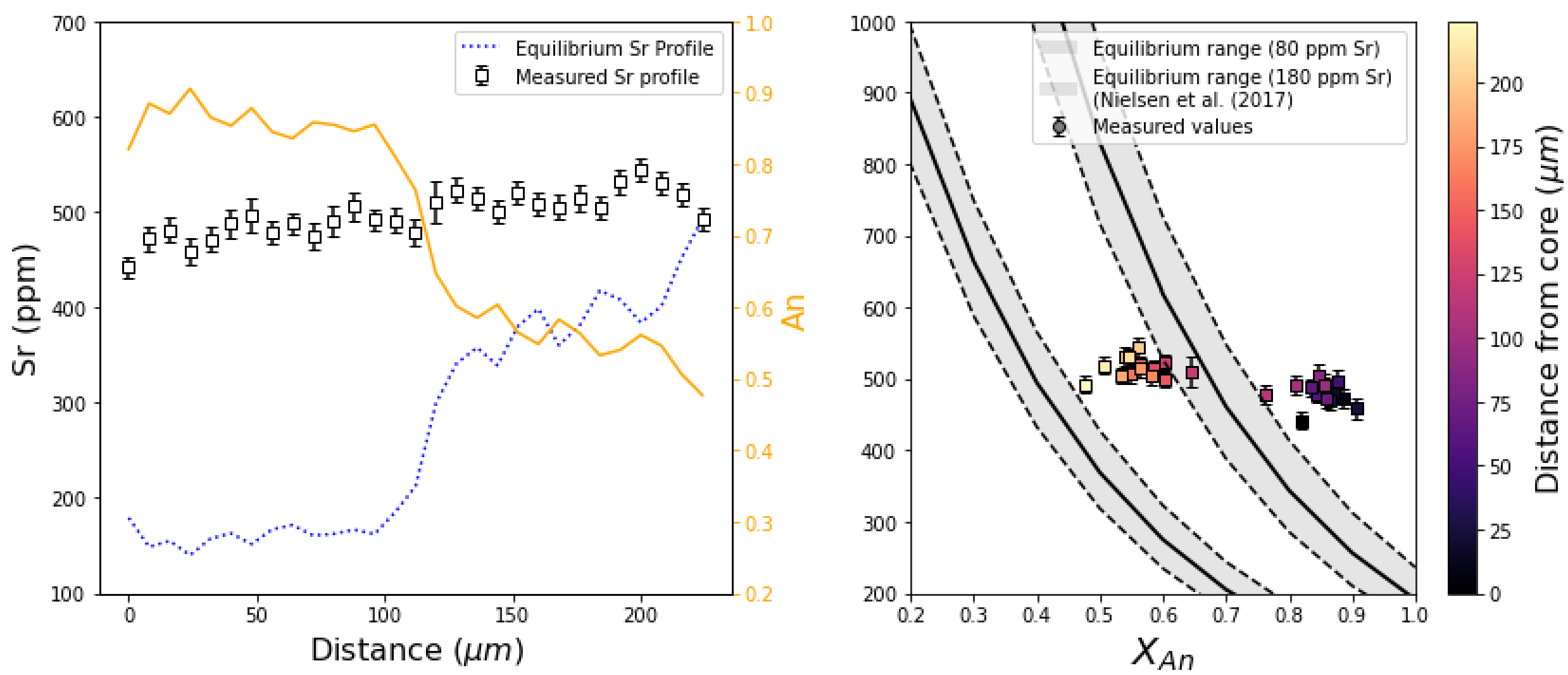

F-A2-01_Plag-3_CORE-1

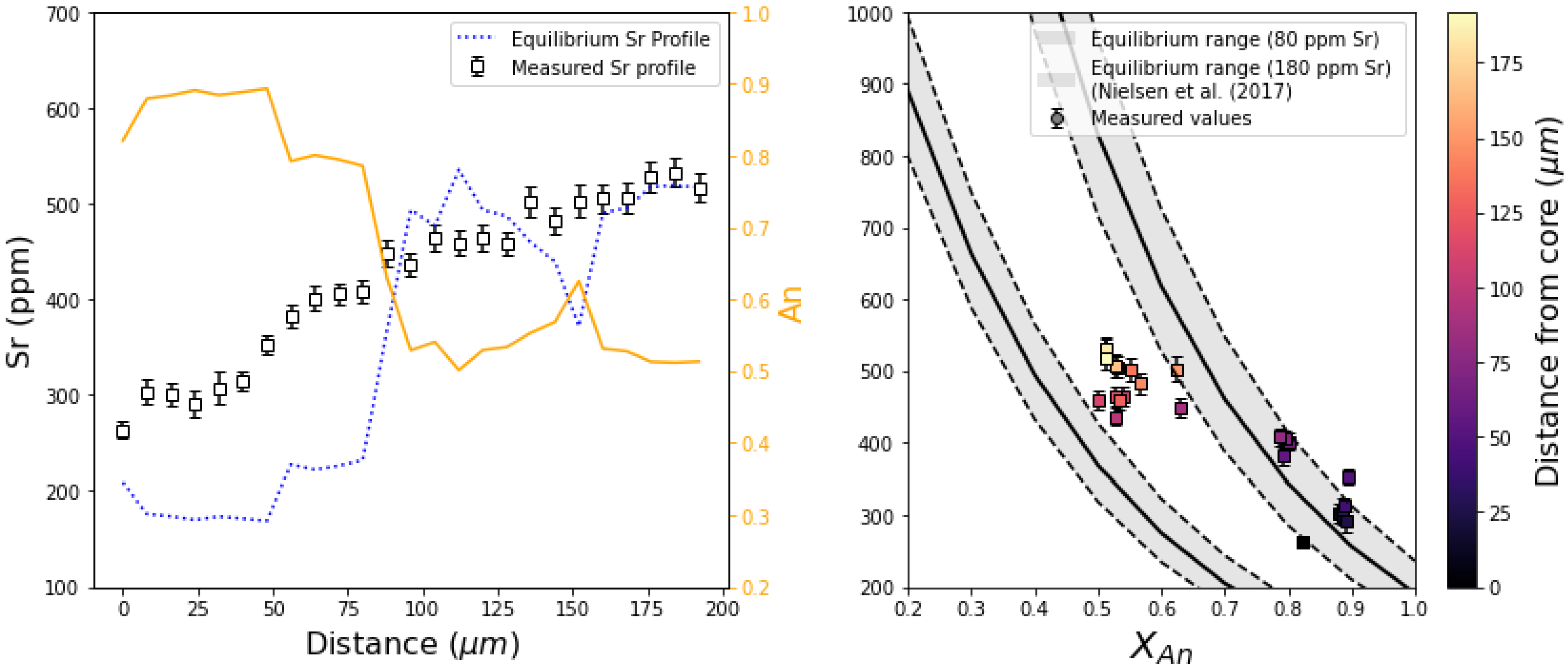

F-A2-01_Plag-5_CORE-1

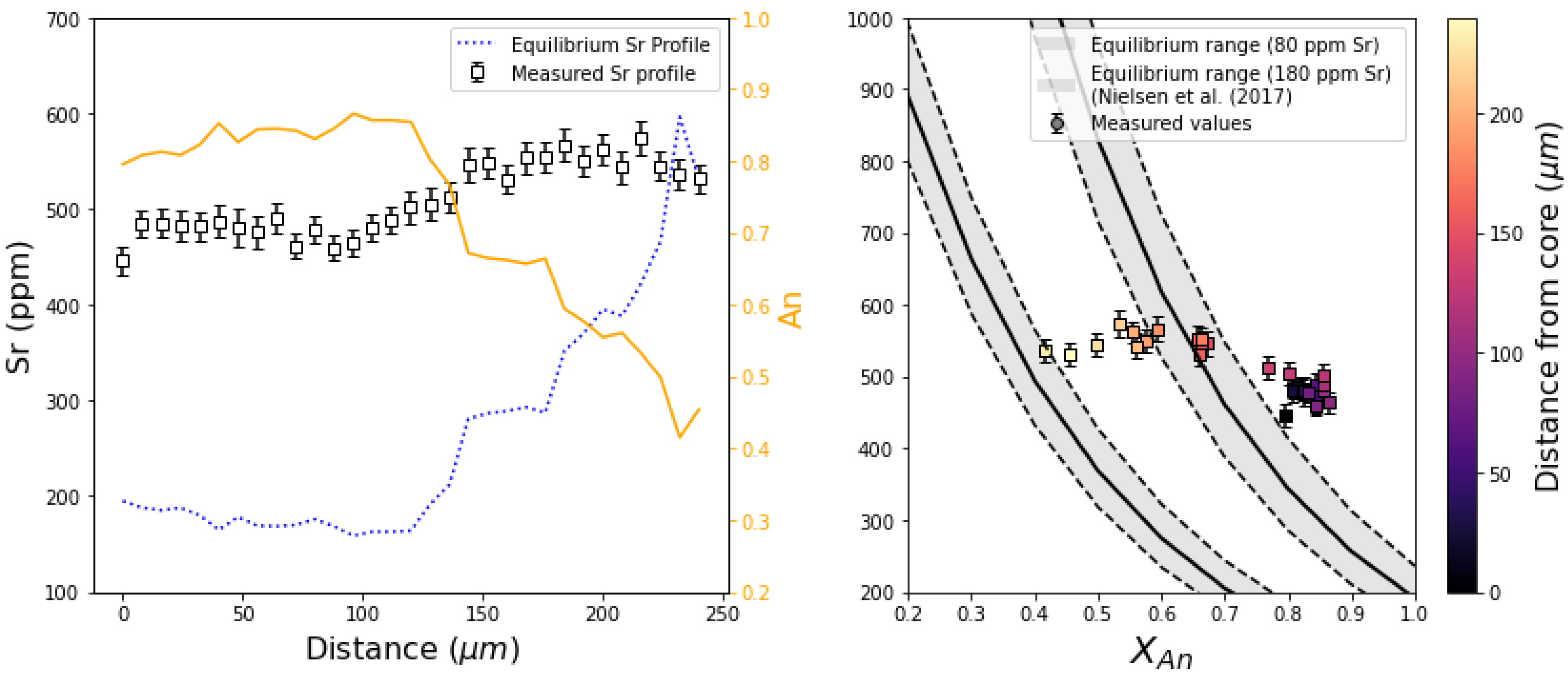

F-A2-01_Plag-8_CORE-1

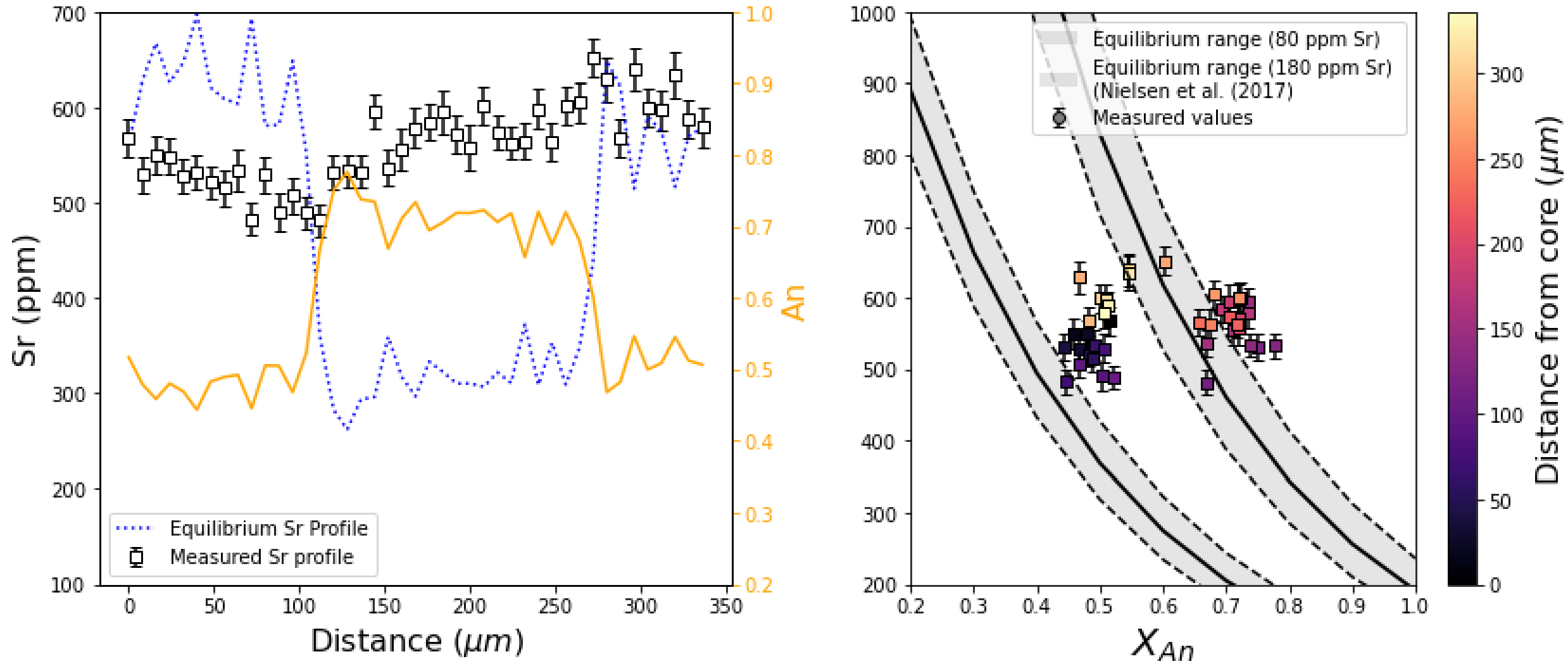

F-A2-01_Plag-15_CORE-1

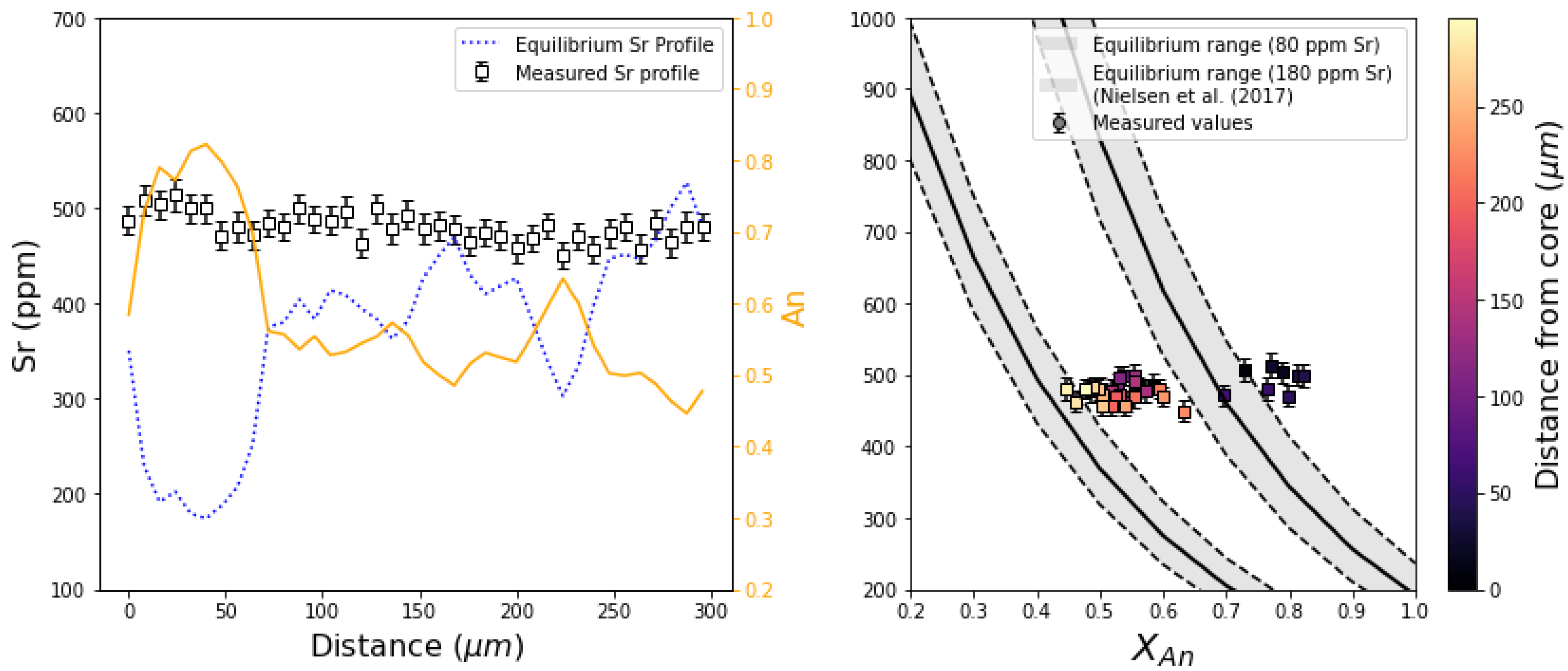

F-A2-02_Plag-1_CORE-1

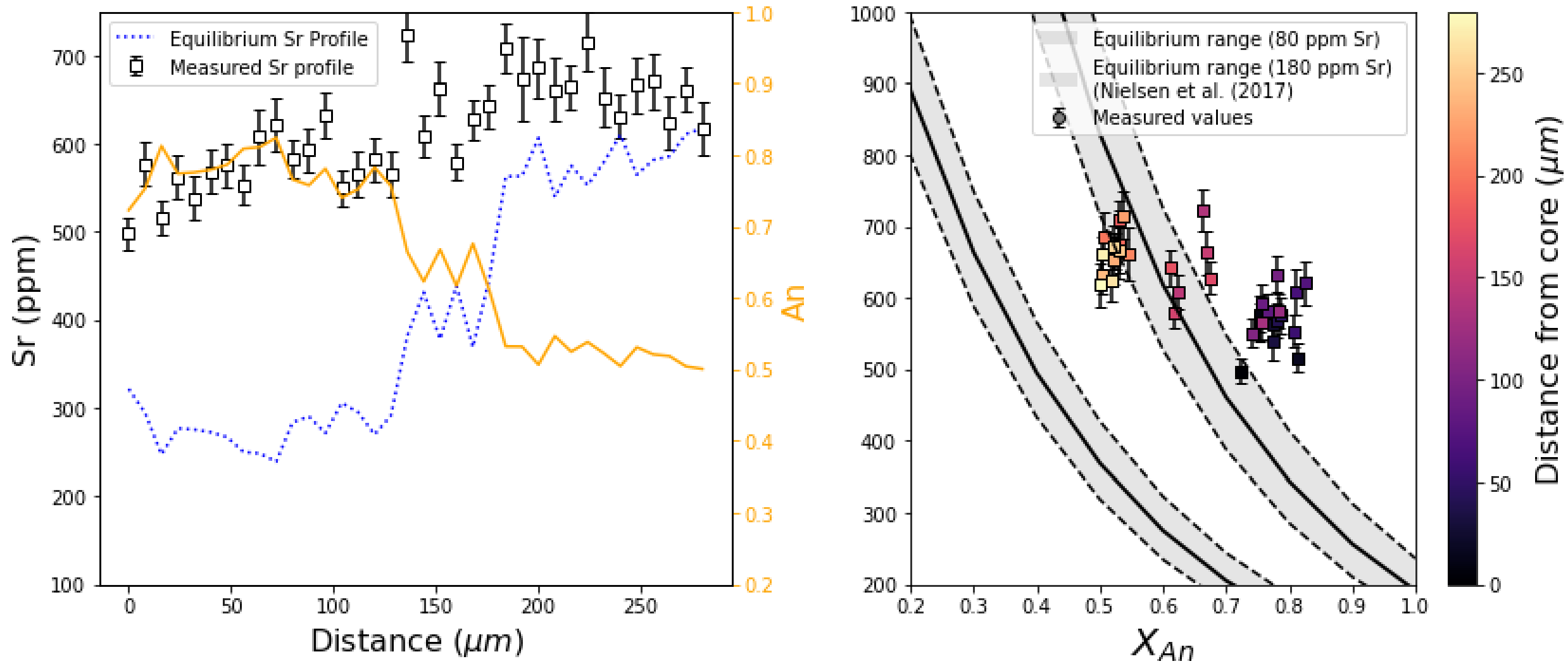

F-A2-02_Plag-4_CORE-1

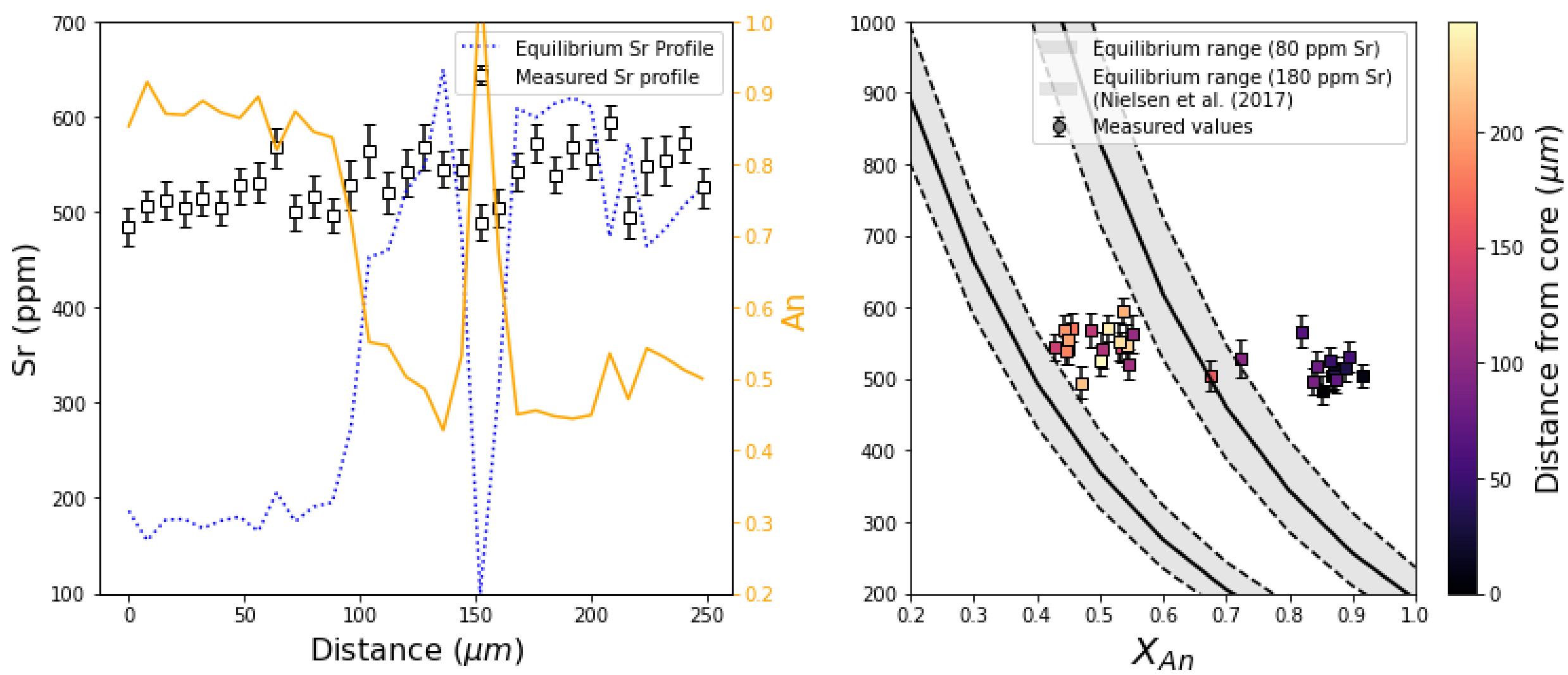

F-A2-02_Plag-9_CORE-1

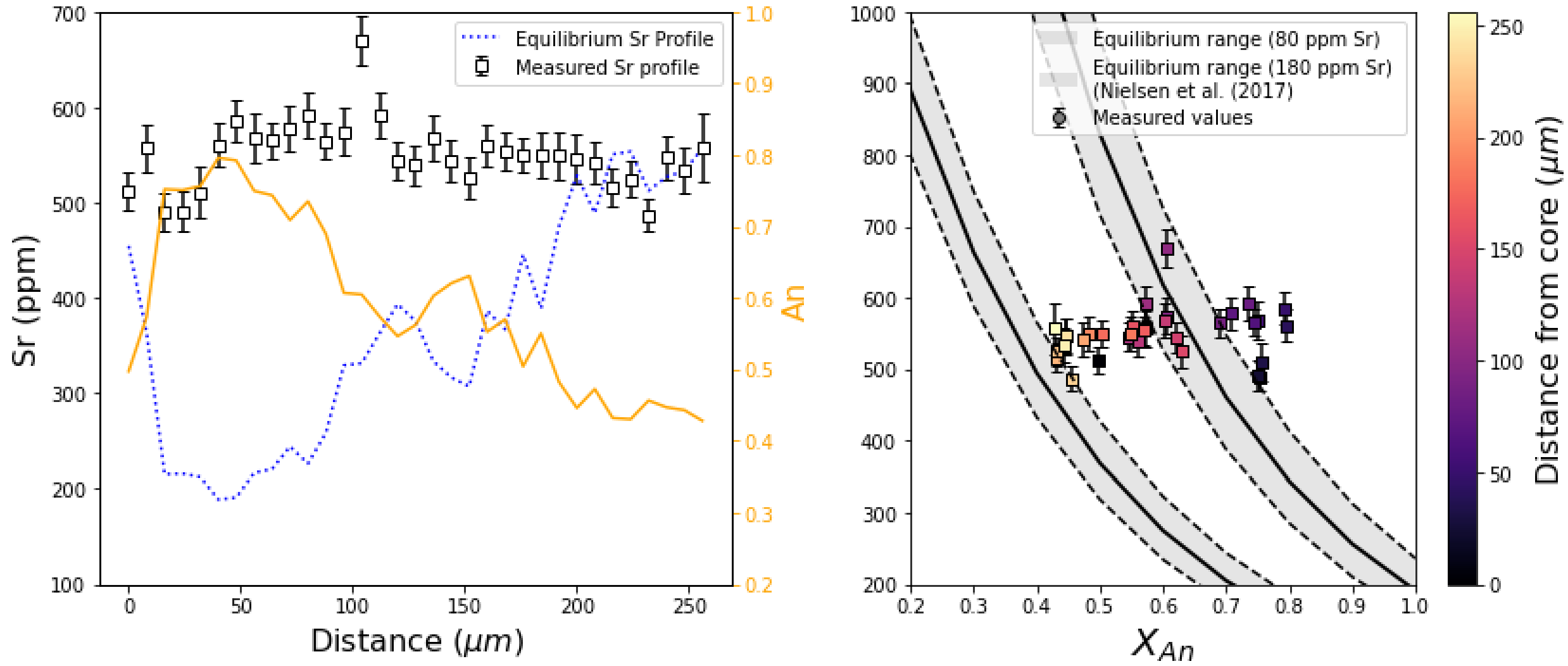

F-A2-02_Plag-9_CORE-6

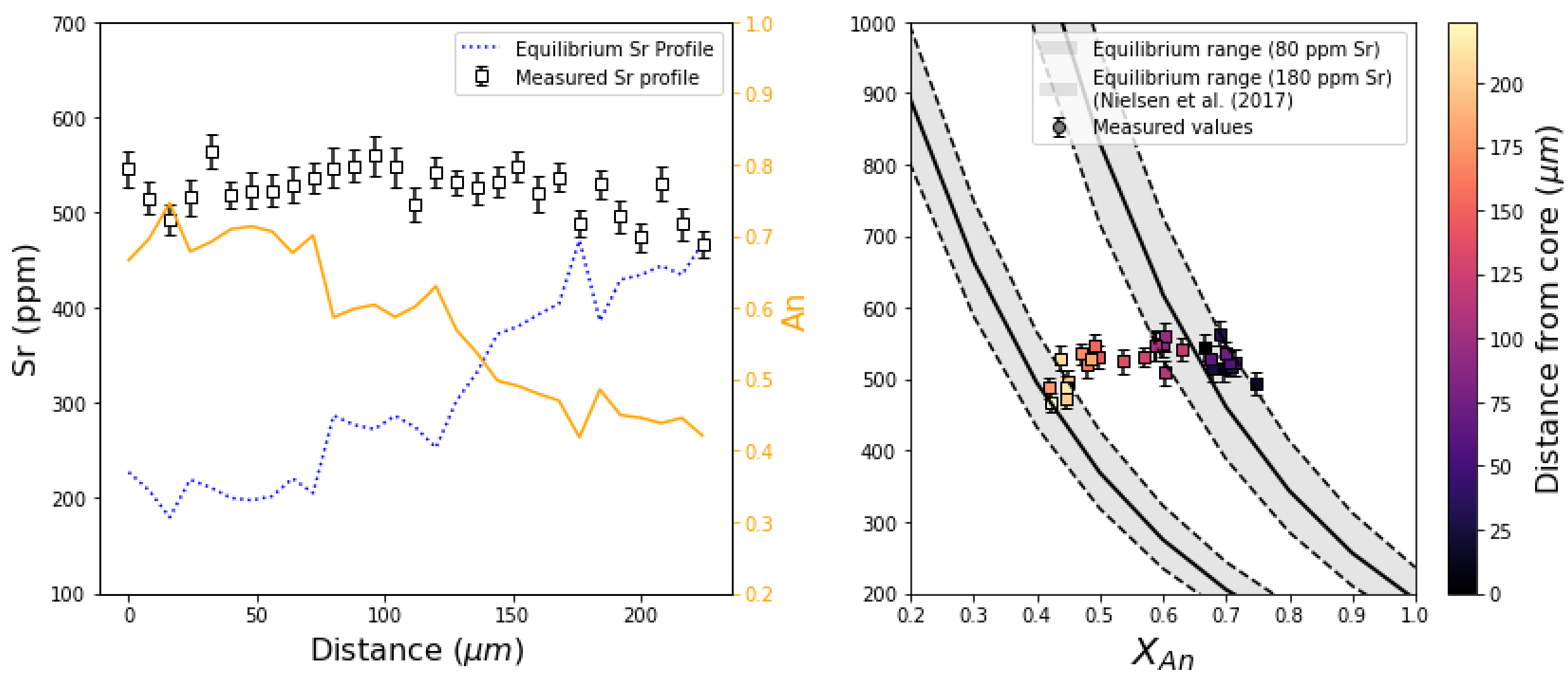

F-A2-02_Plag-14_CORE-1

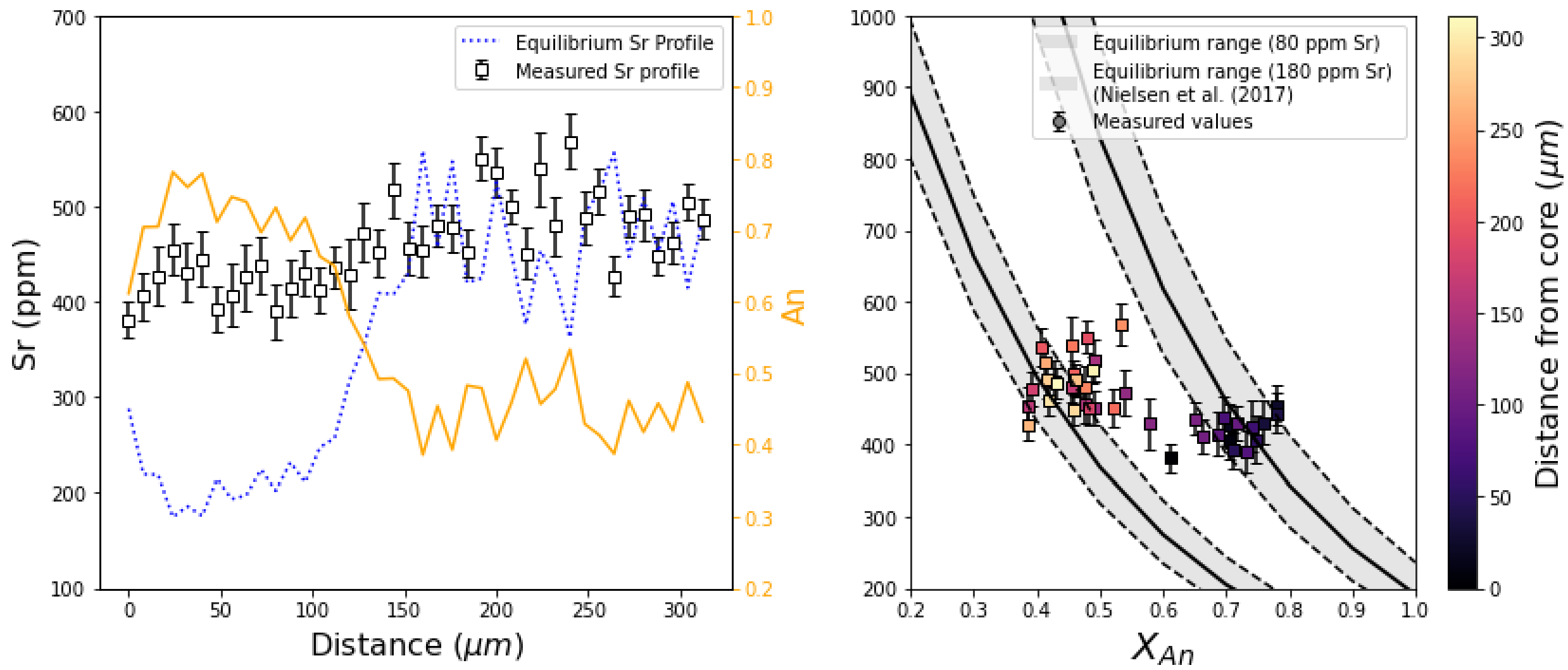

F-A2-02_Plag-14_CORE-2

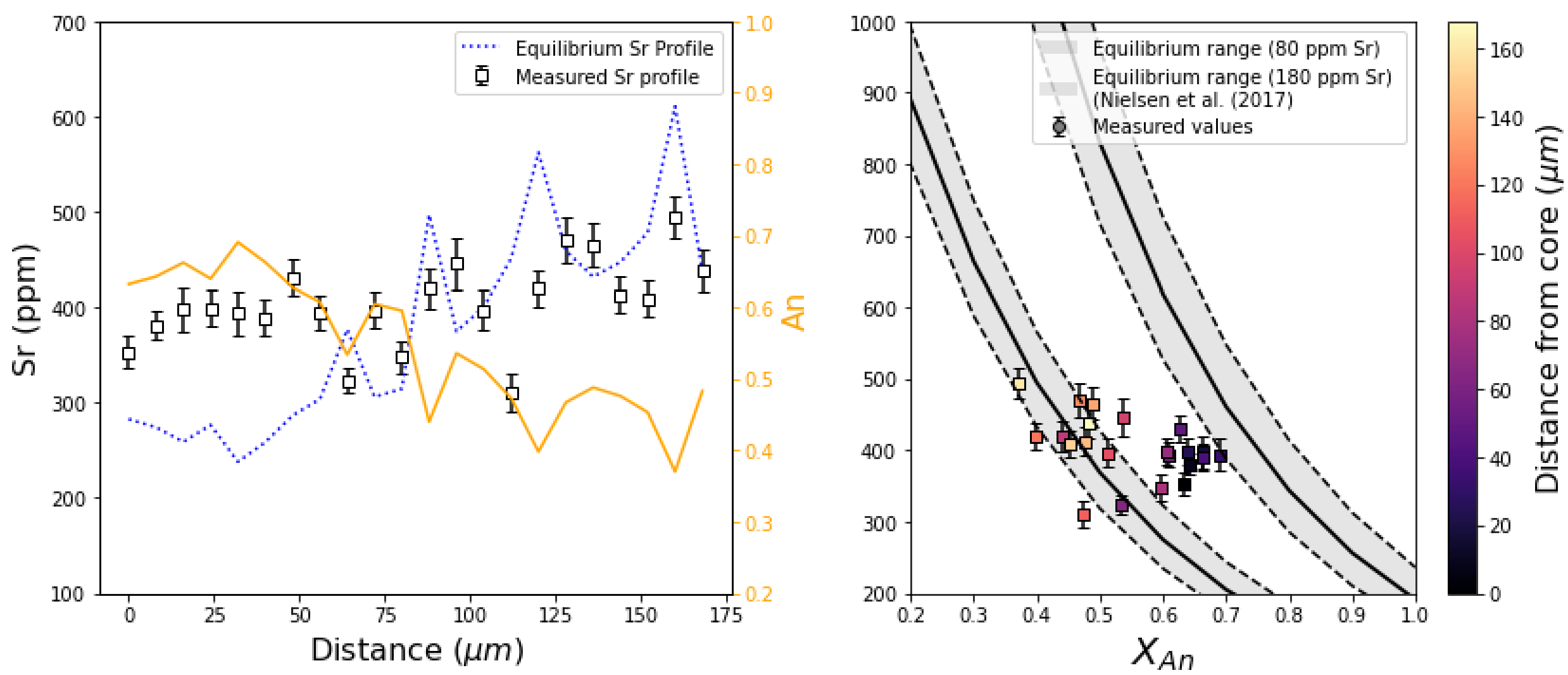

F-A2-02_Plag-3_CORE-2

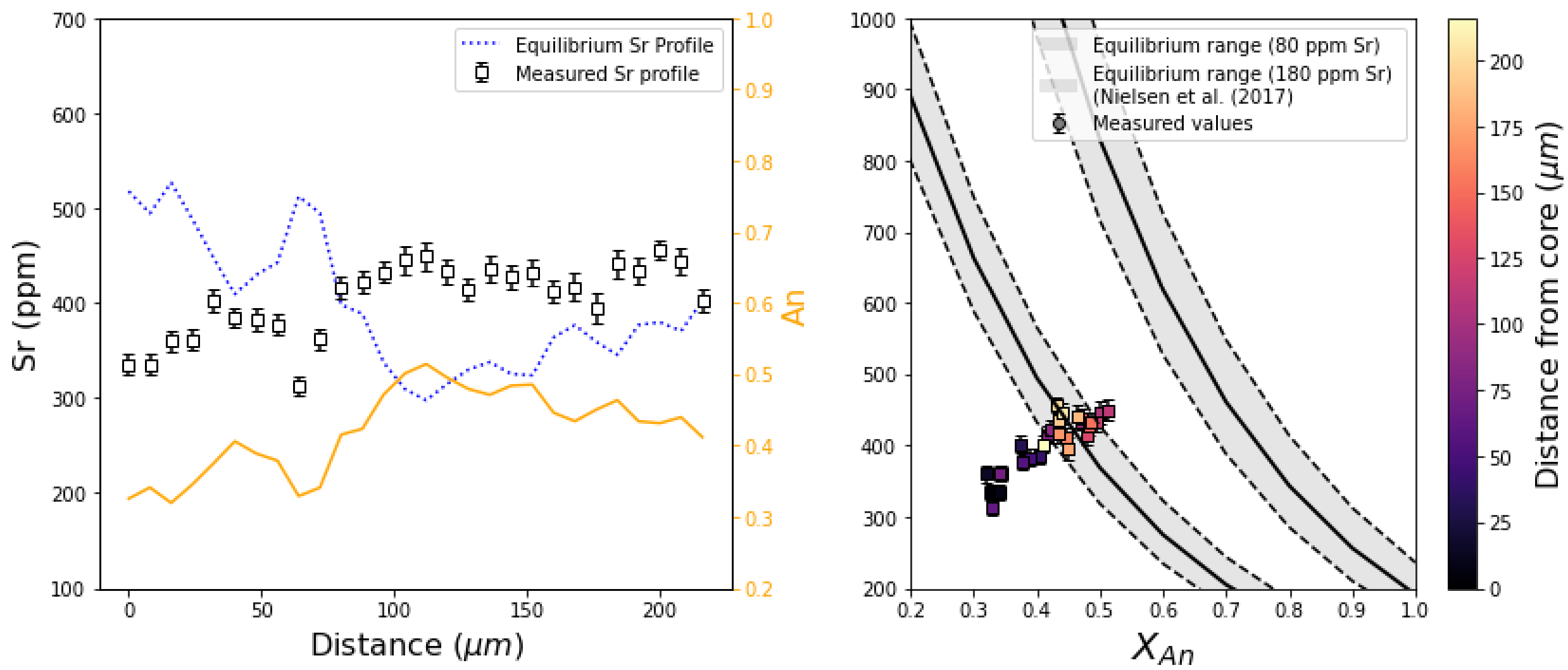

A-03_Plag-4_CORE-1

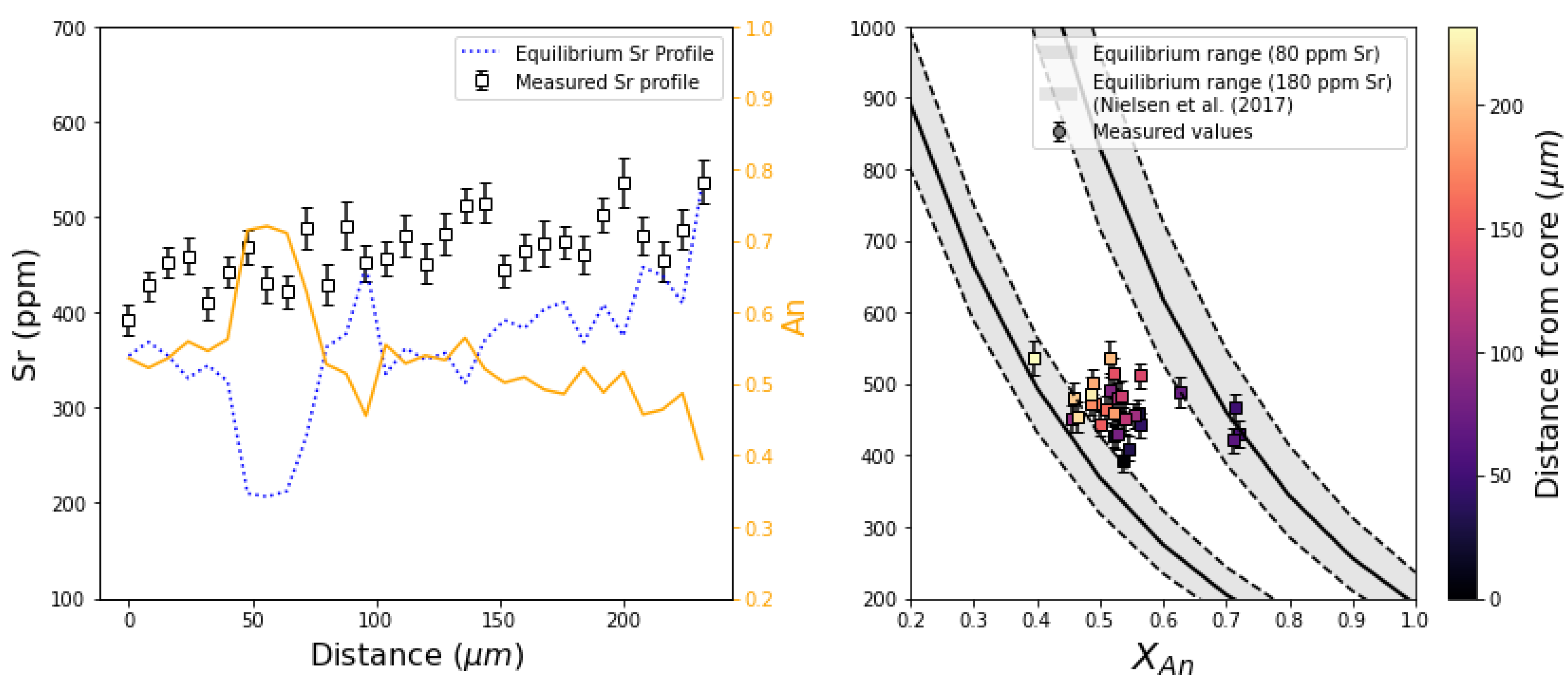

A-03_Plag-4_CORE-2

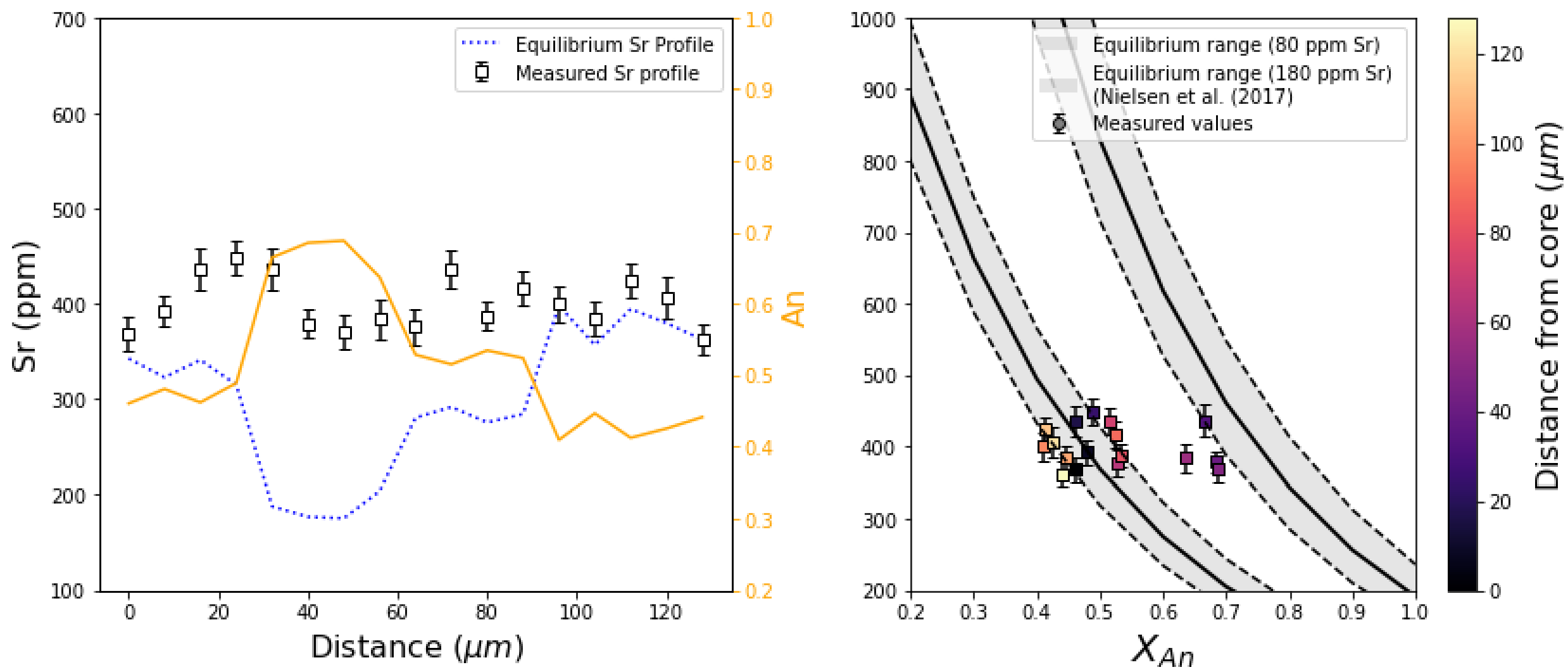

A-03_Plag-5_CORE-2

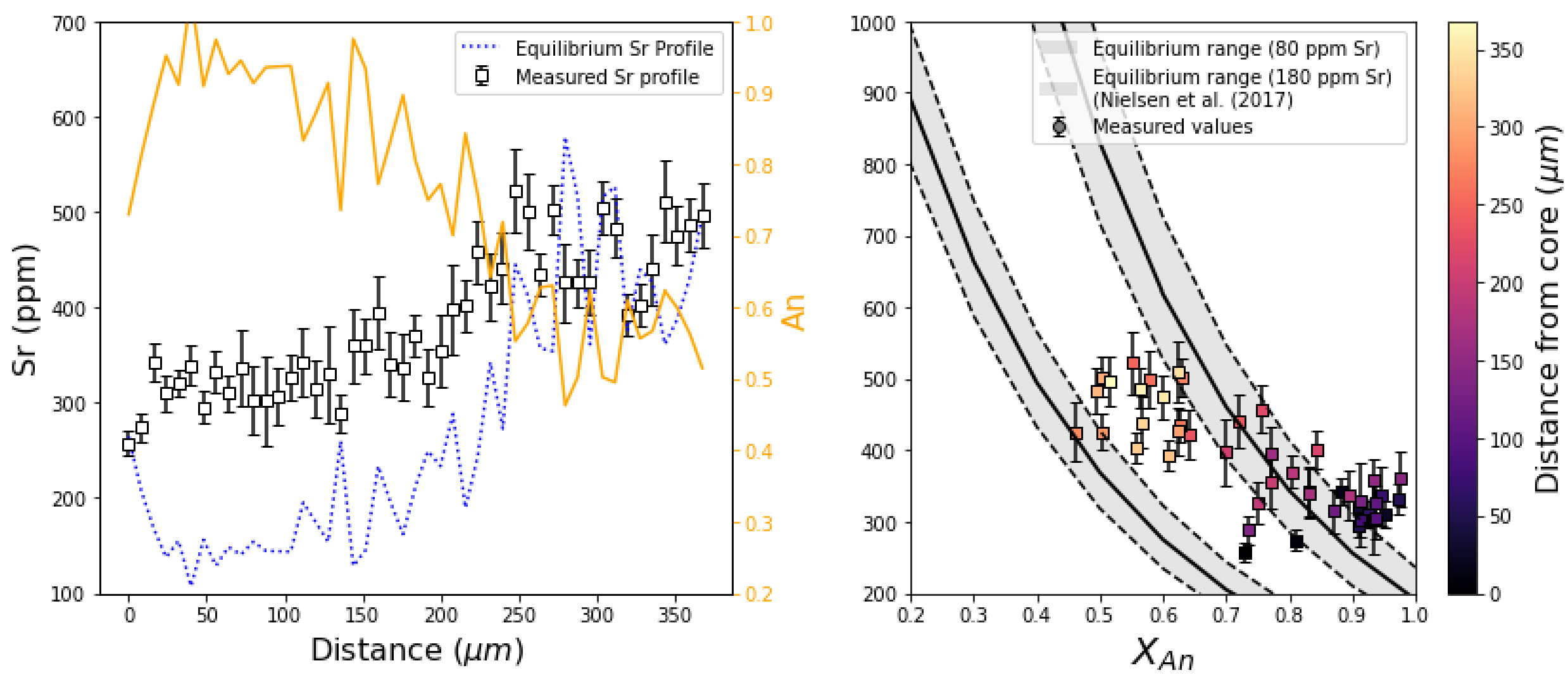

A-03_Plag-5_CORE-1

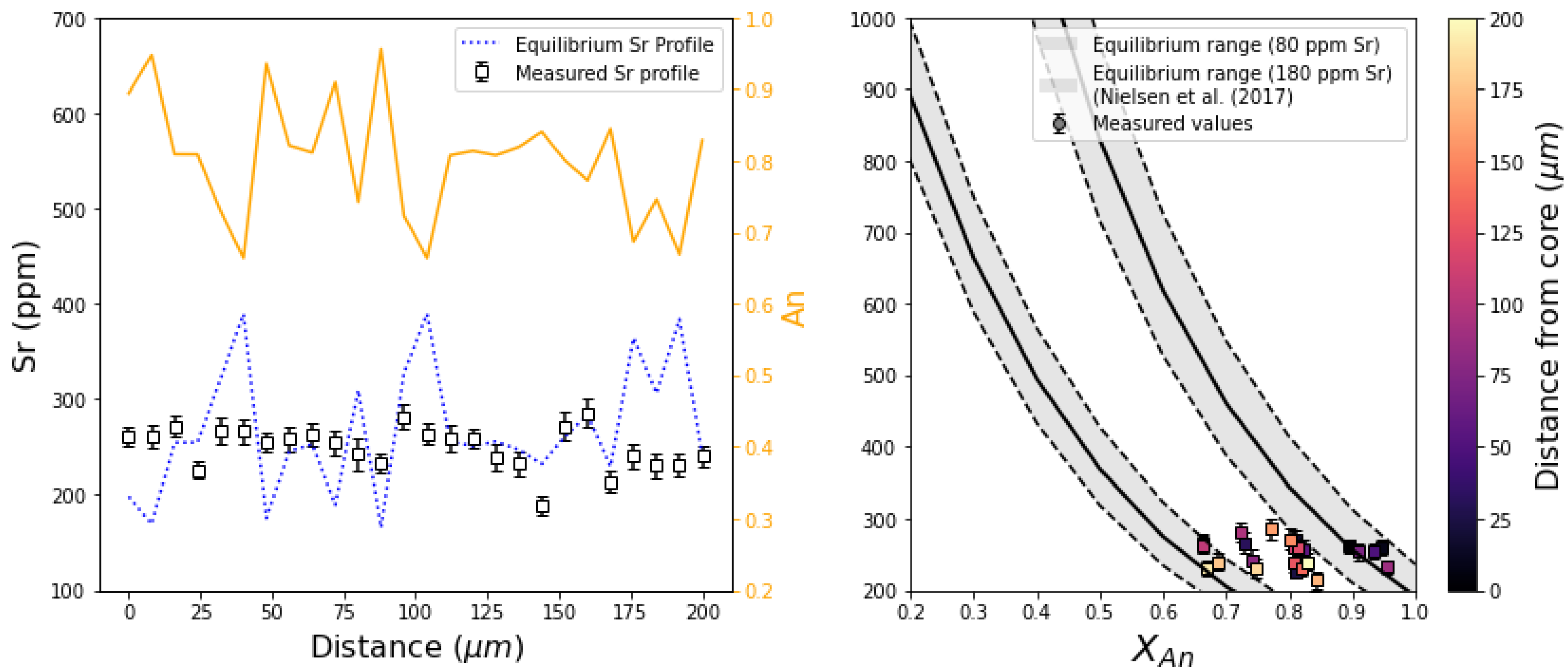

A-03_Plag-7_CORE-3

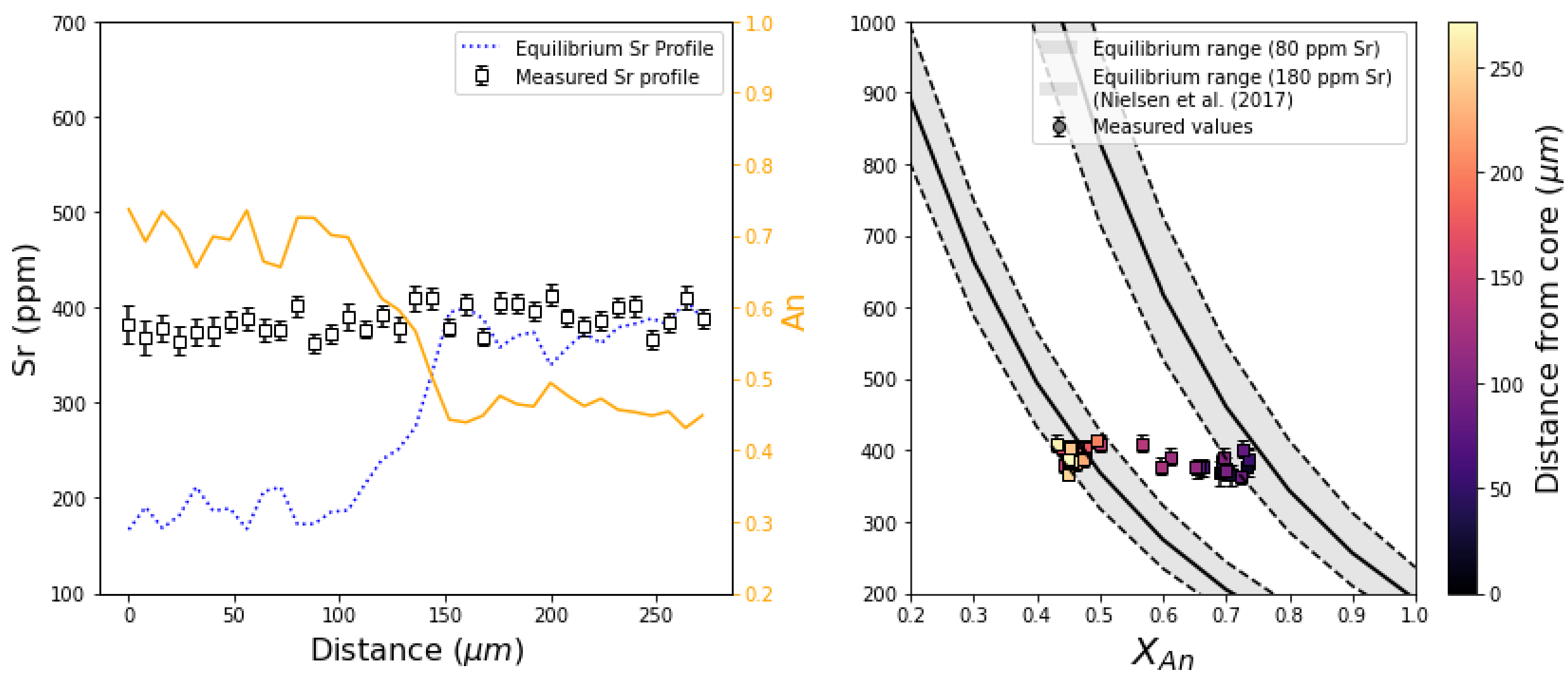

A-03_Plag-7_CORE-4

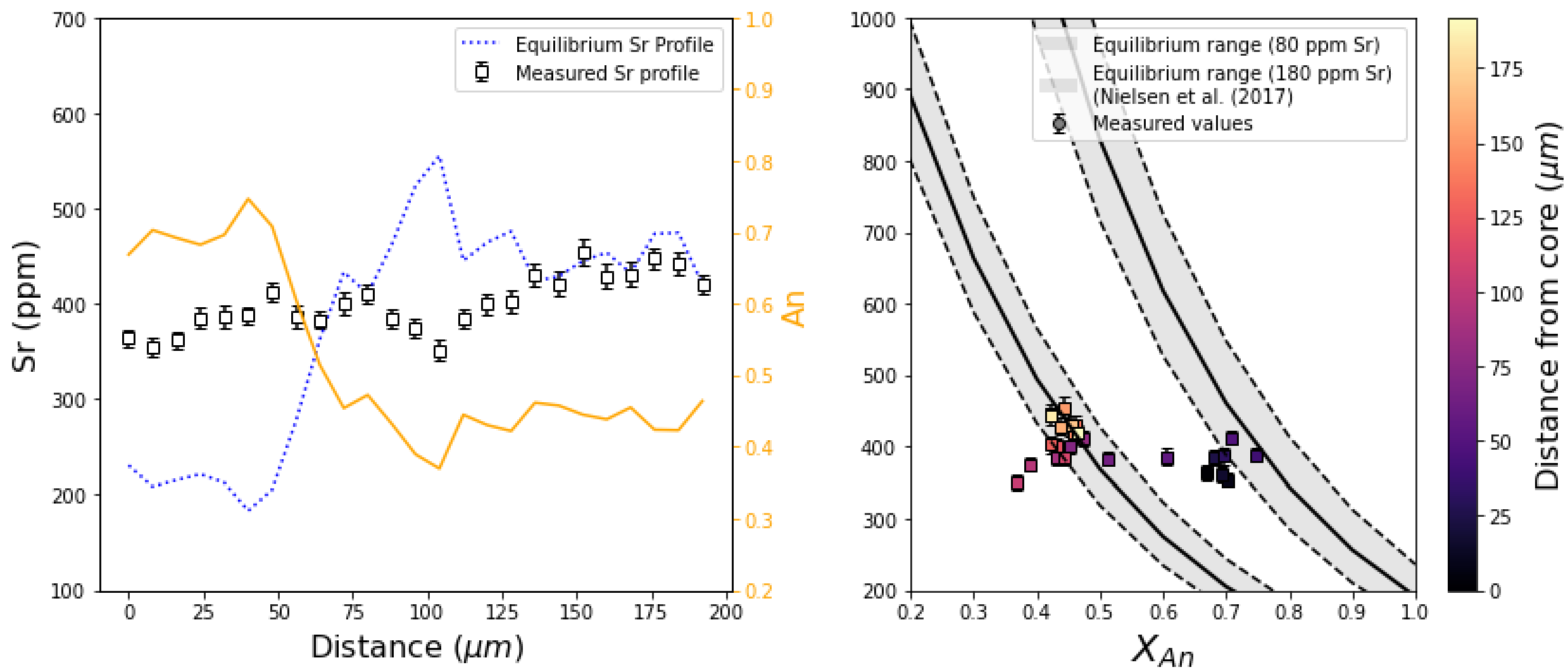

A-10_Plag-3_CORE-1

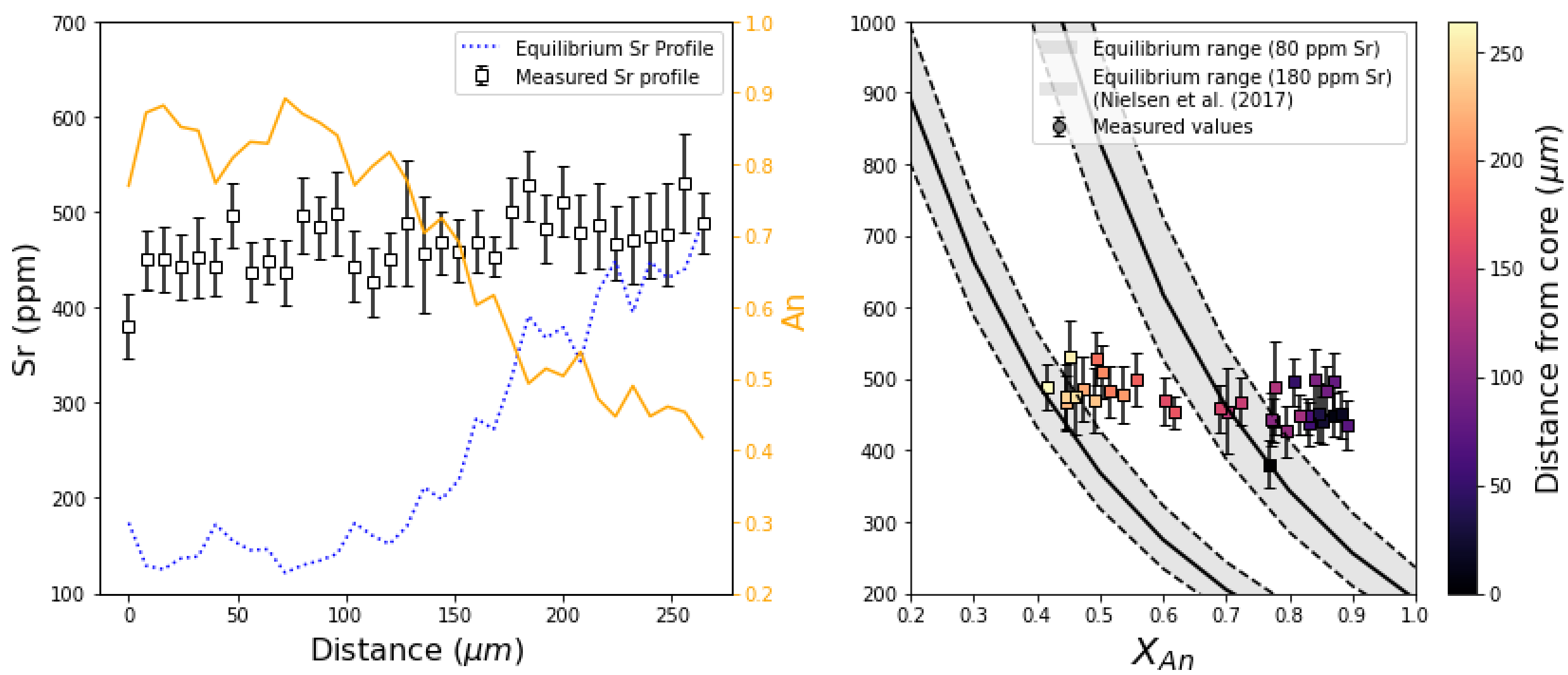

RIMS

ITO-D1-01_Plag-1_RIM-1

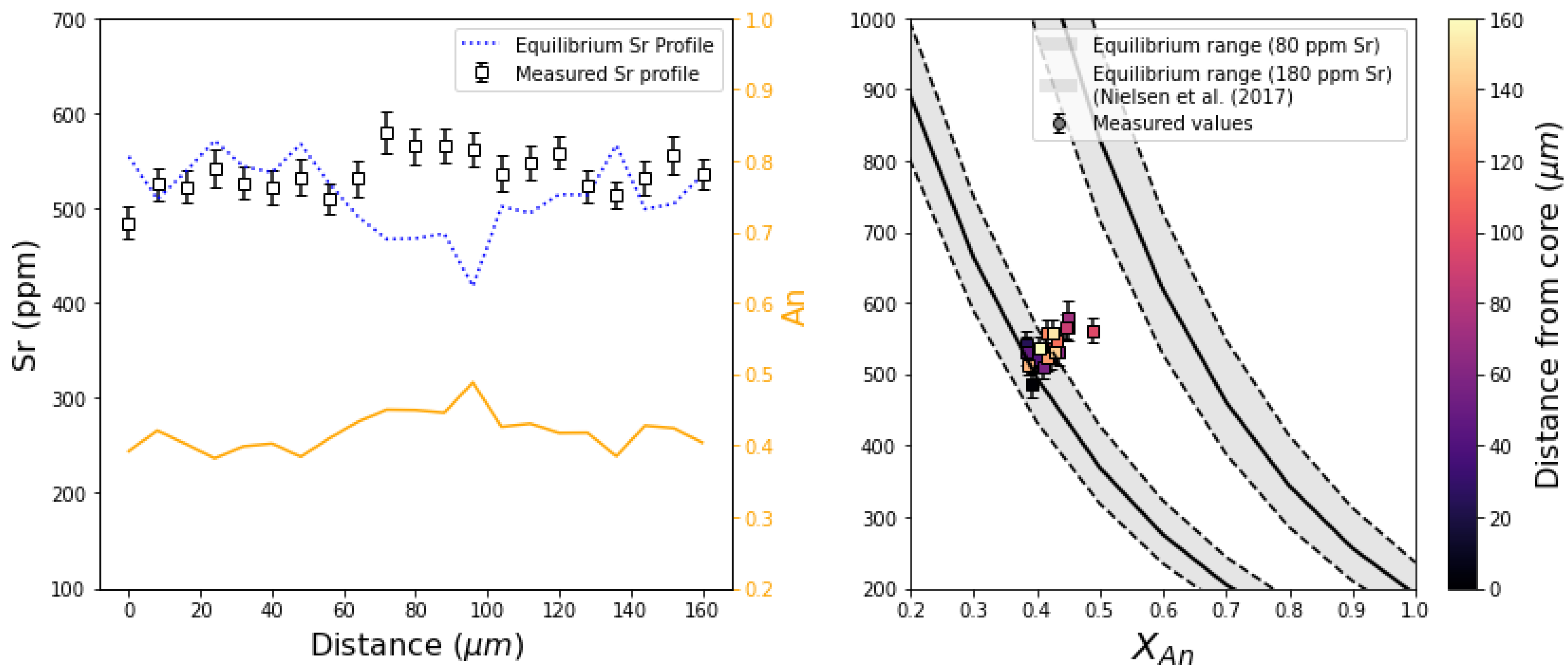

ITO-D1-01_Plag-2_RIM-1

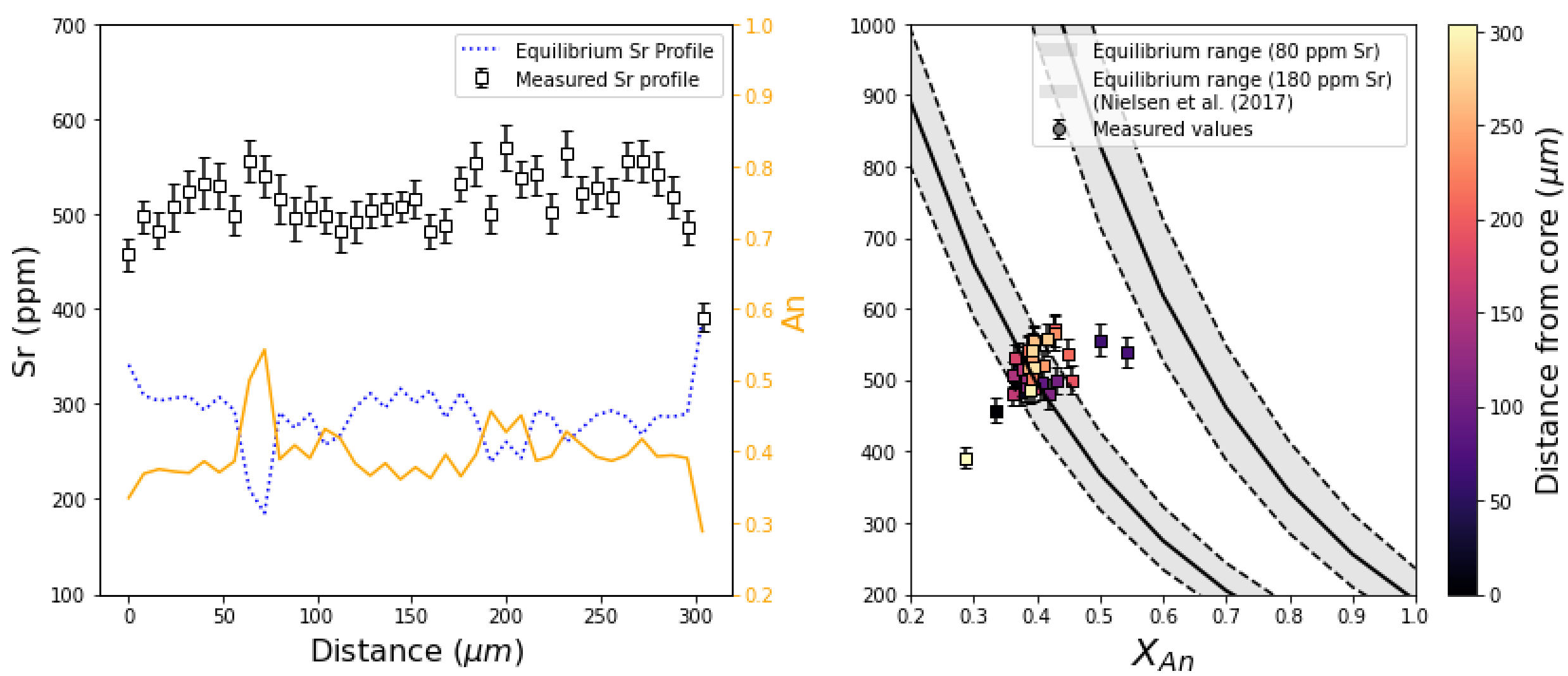

ITO-D1-01_Plag-4_RIM-1

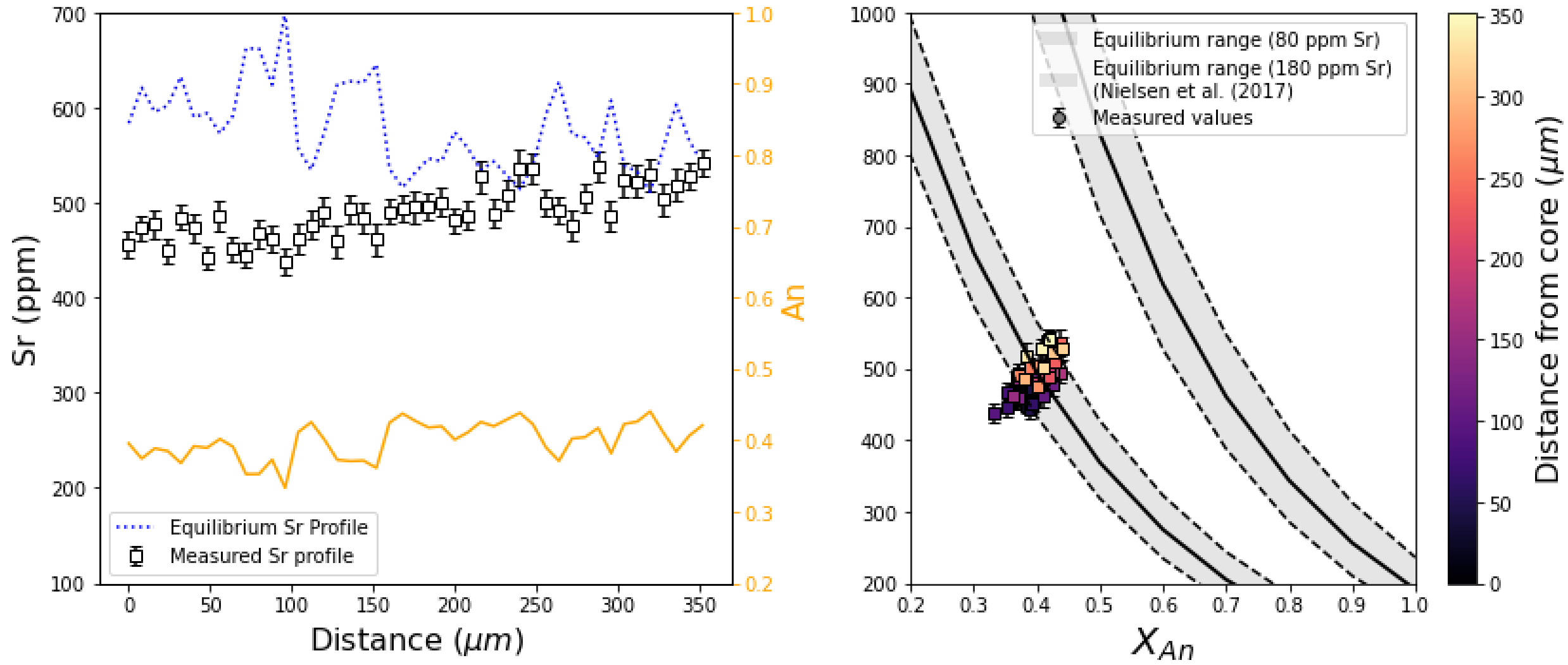

ITO-D1-01_Plag-9_RIM-1

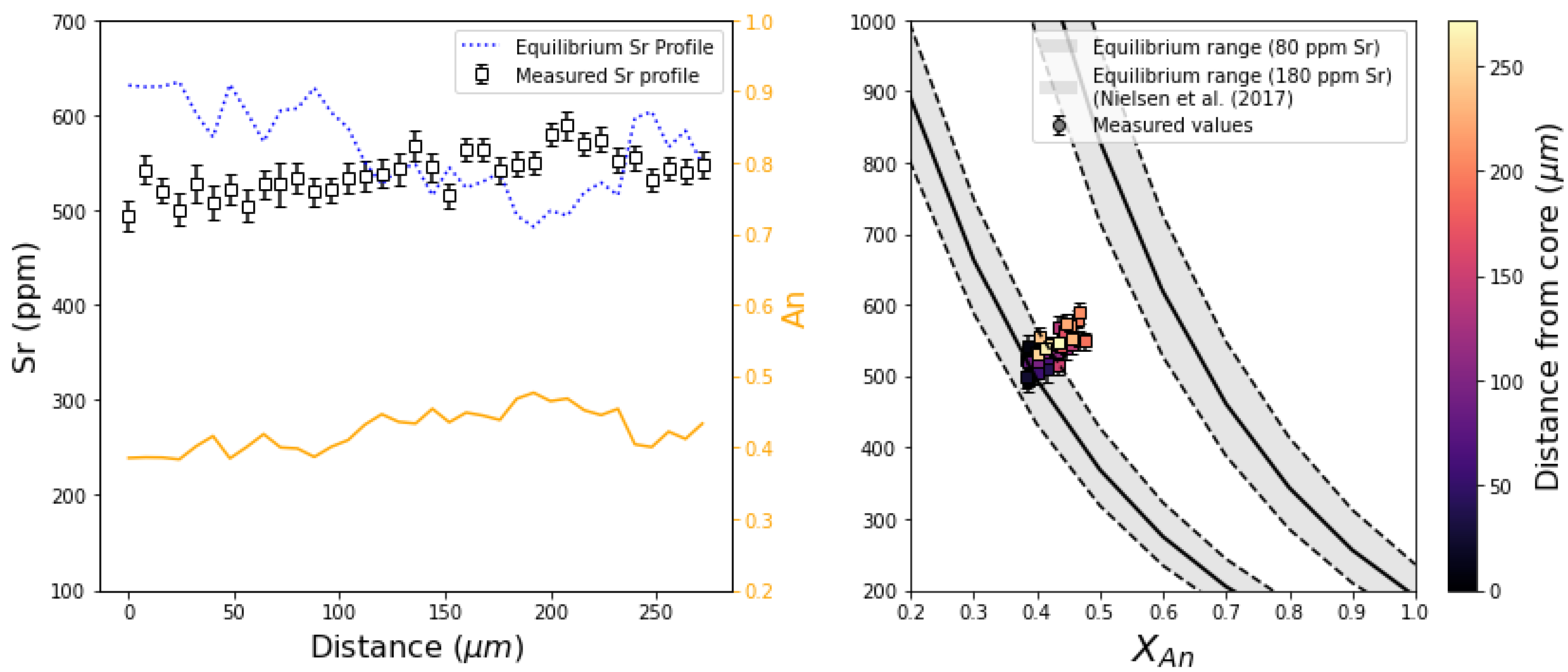

ITO-D1-01_Plag-10_RIM-1

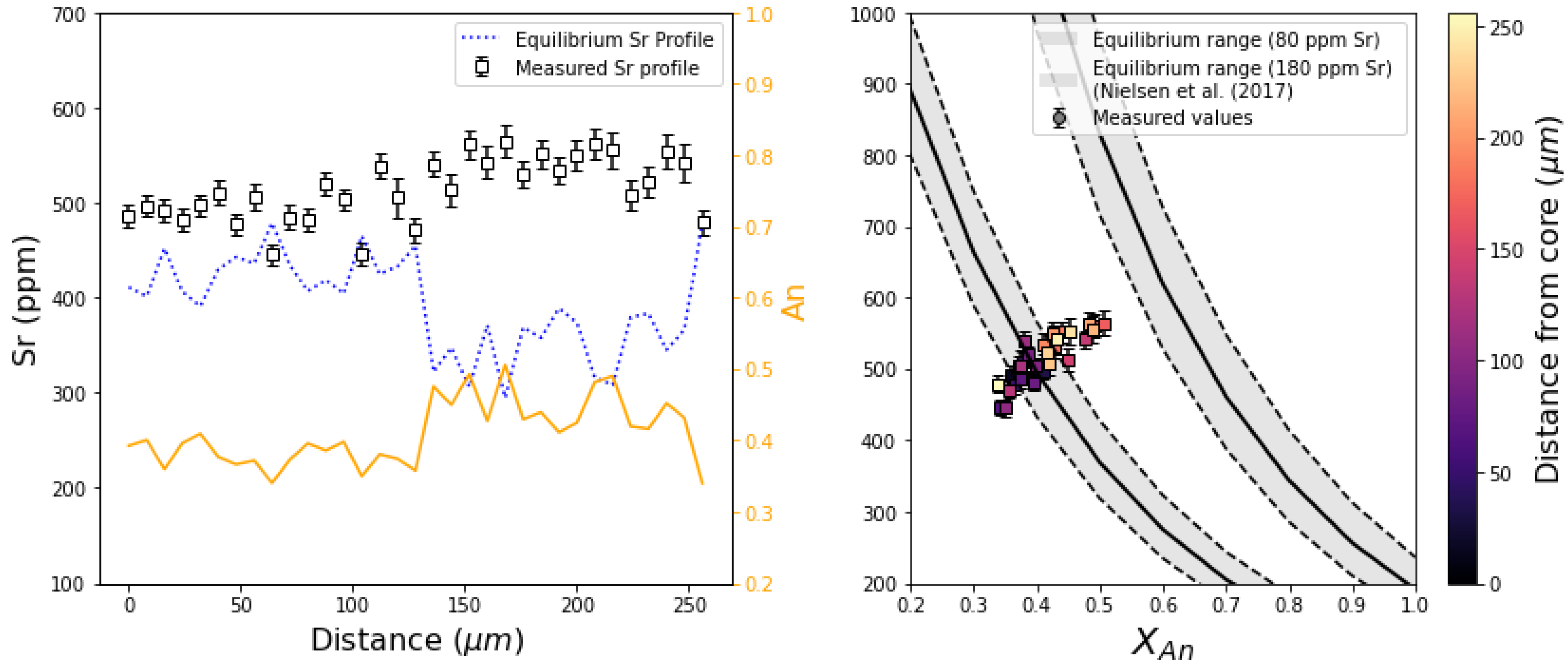

ITO-D1-02_Plag-1_RIM-1

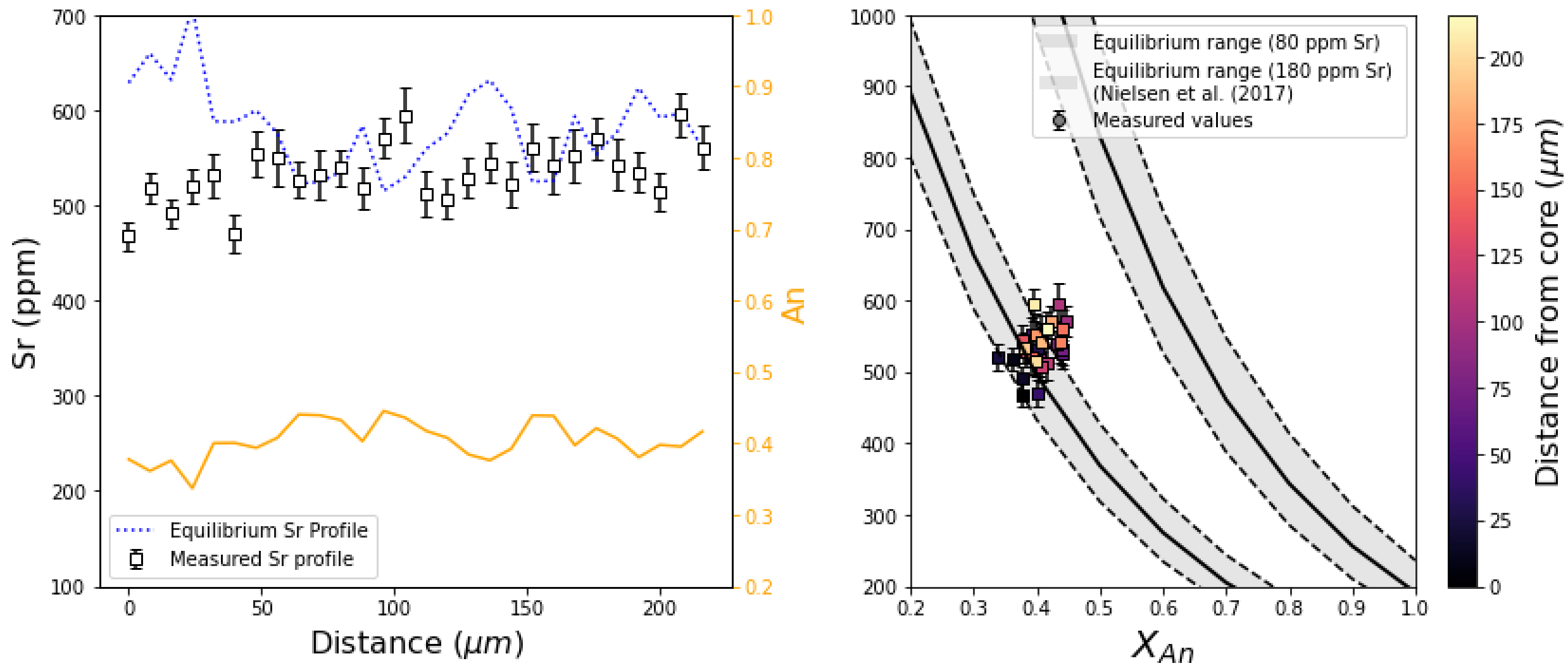

ITO-D1-02_Plag-4_RIM-1

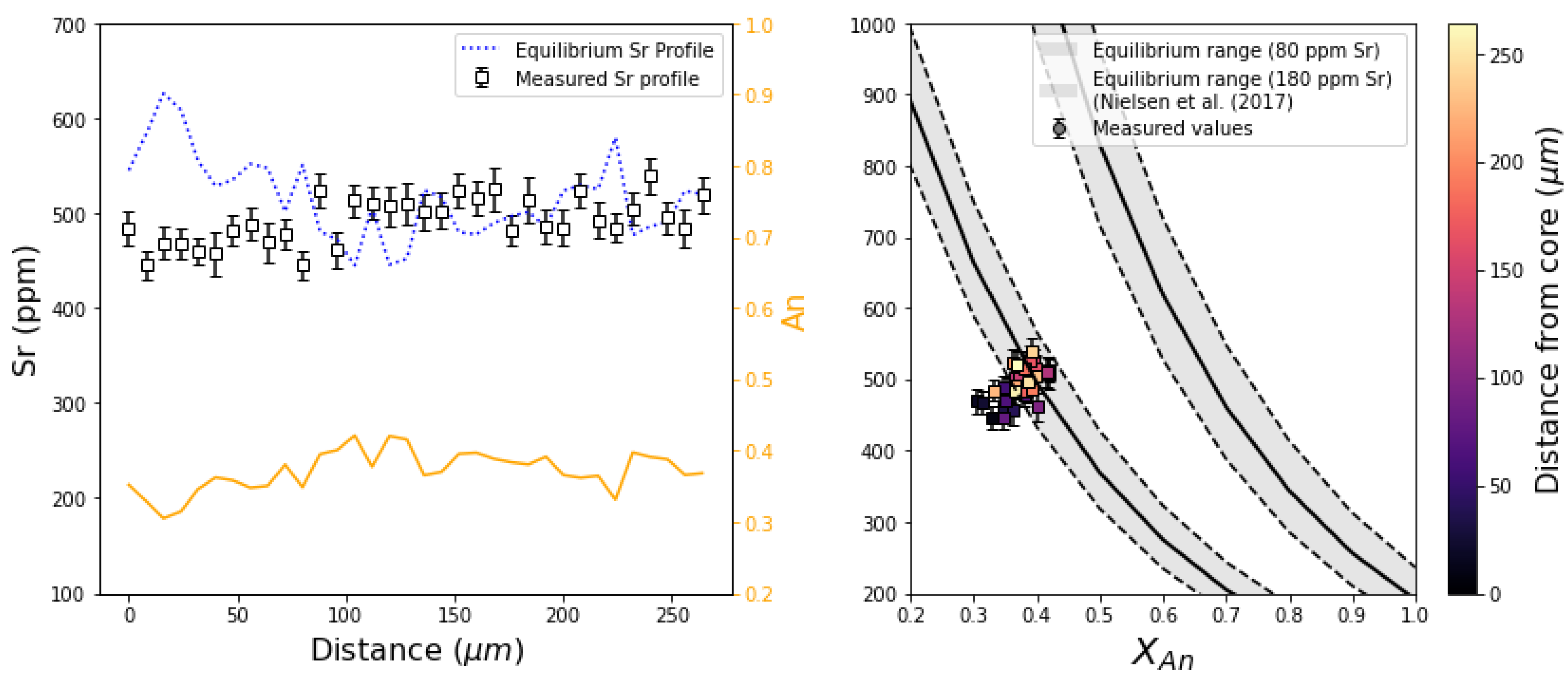

ITO-D1-02_Plag-10_RIM-1

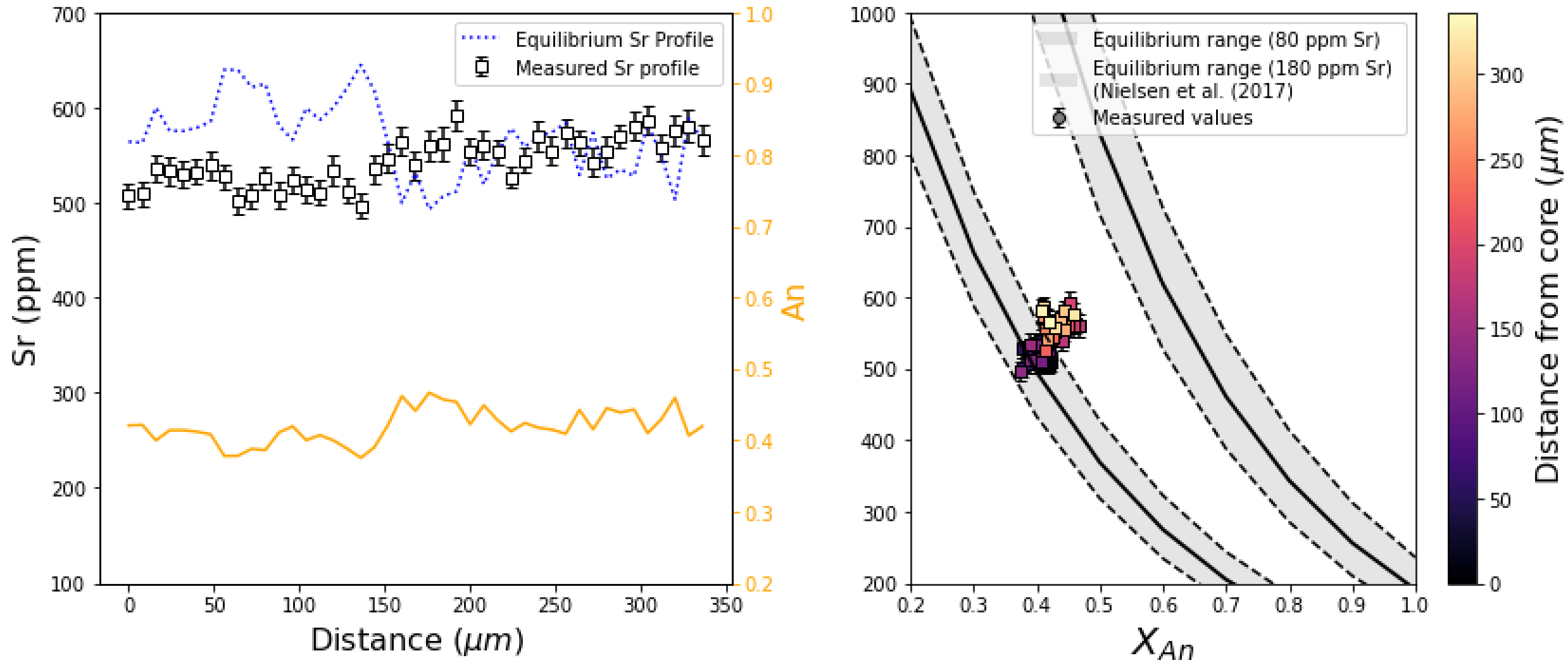

ITO-D1-02_Plag-10_RIM-2

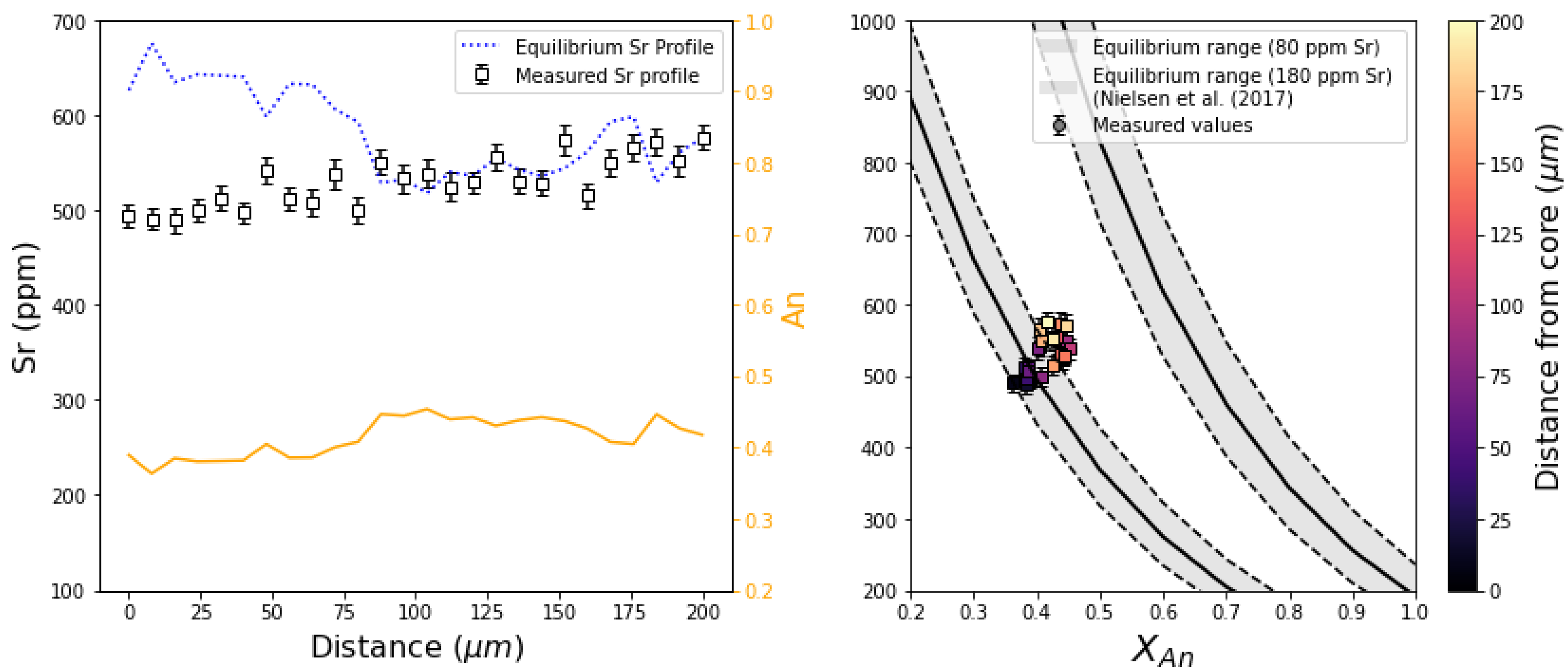

ITO-D1-02_Plag-15_RIM-1

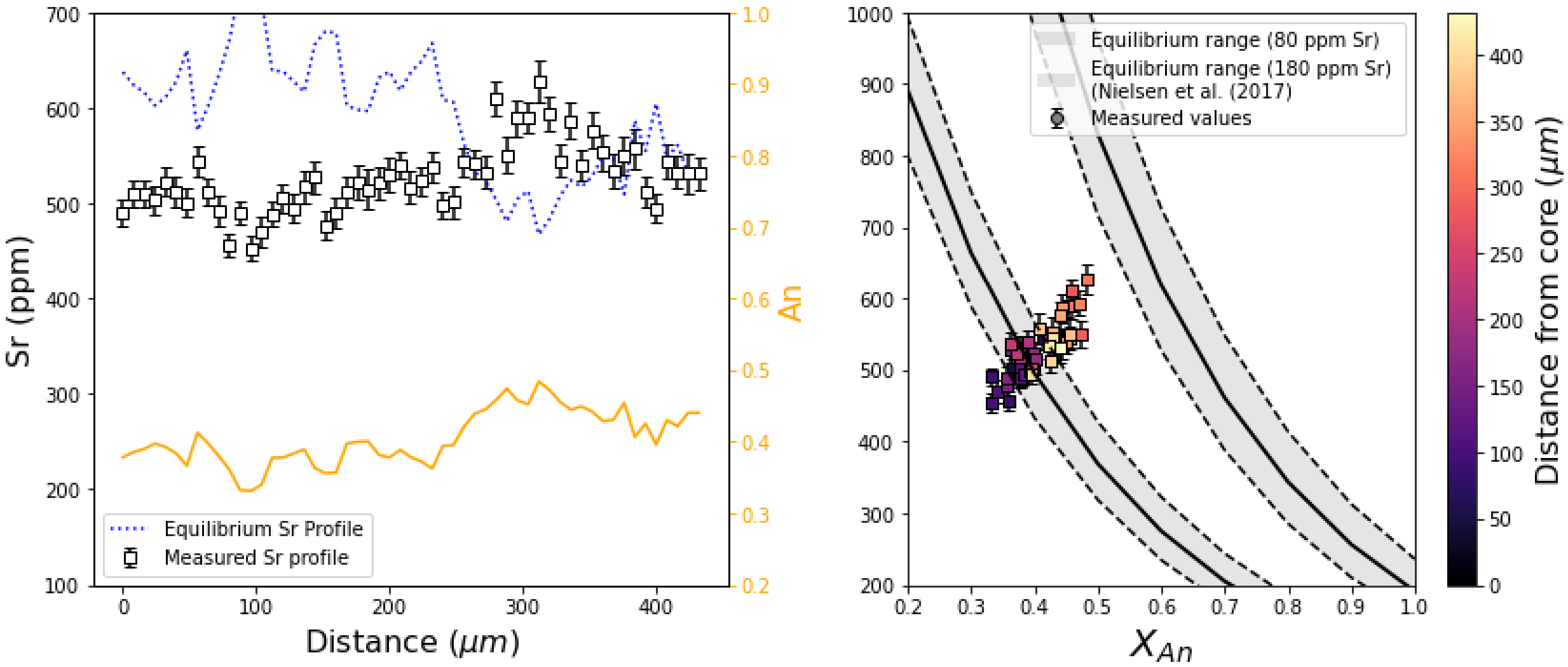

ITO-D2-01_Plag-4_RIM-1

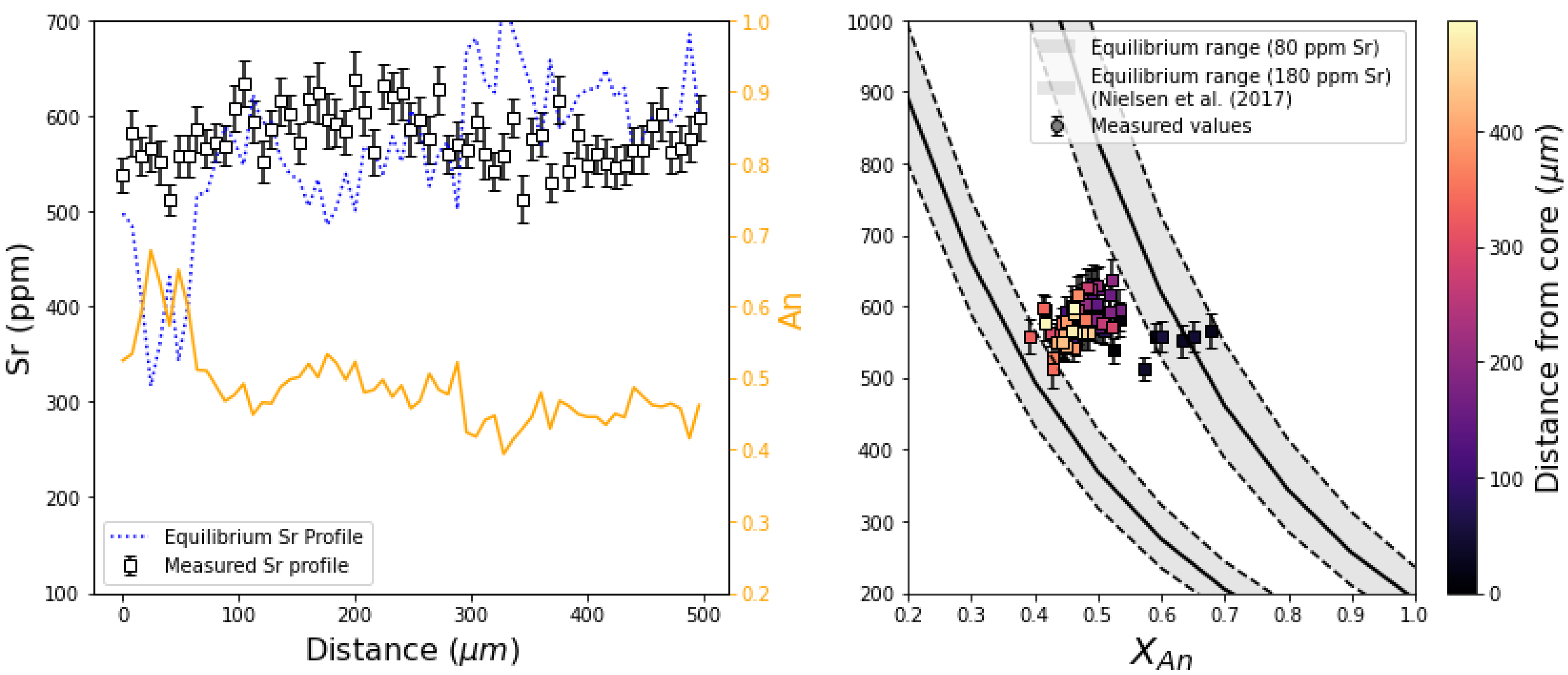

ITO-D2-01_Plag-11_RIM-1

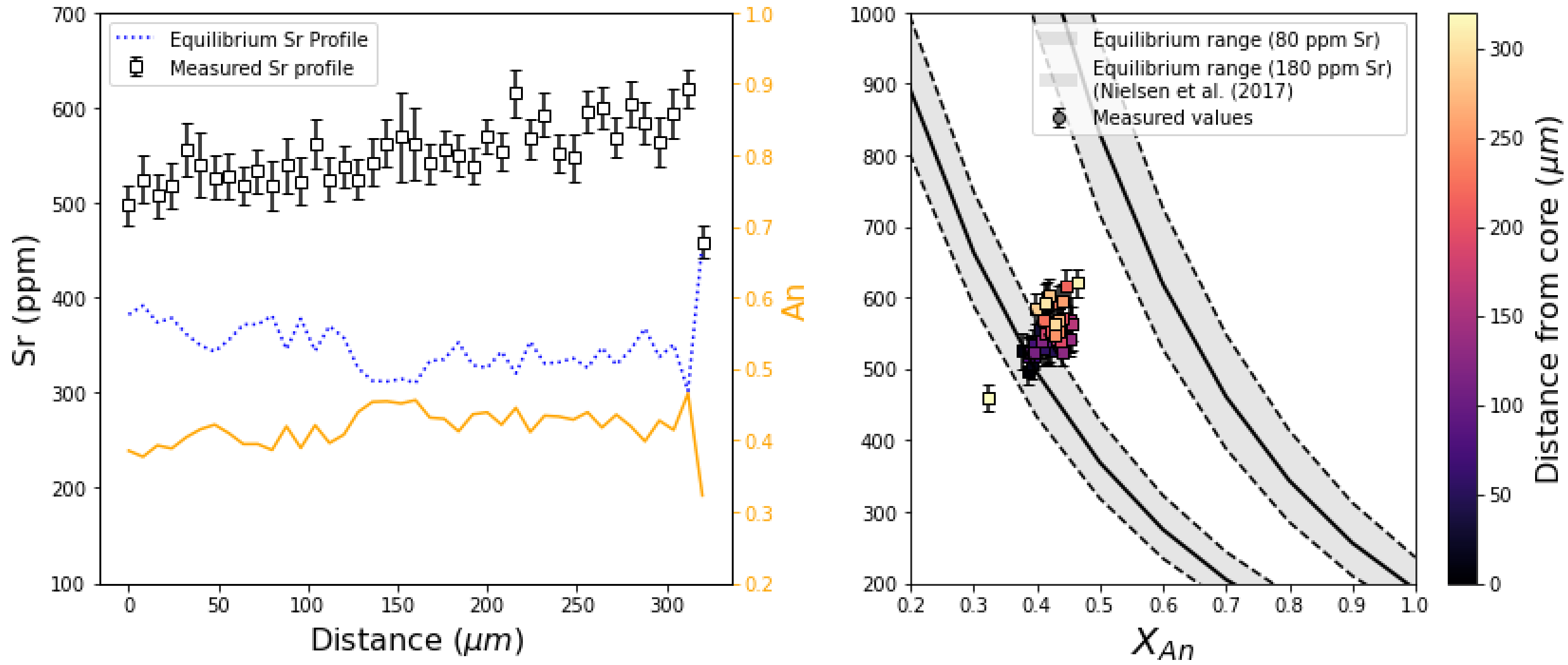

OS-B1-01_Plag-1_RIM-1

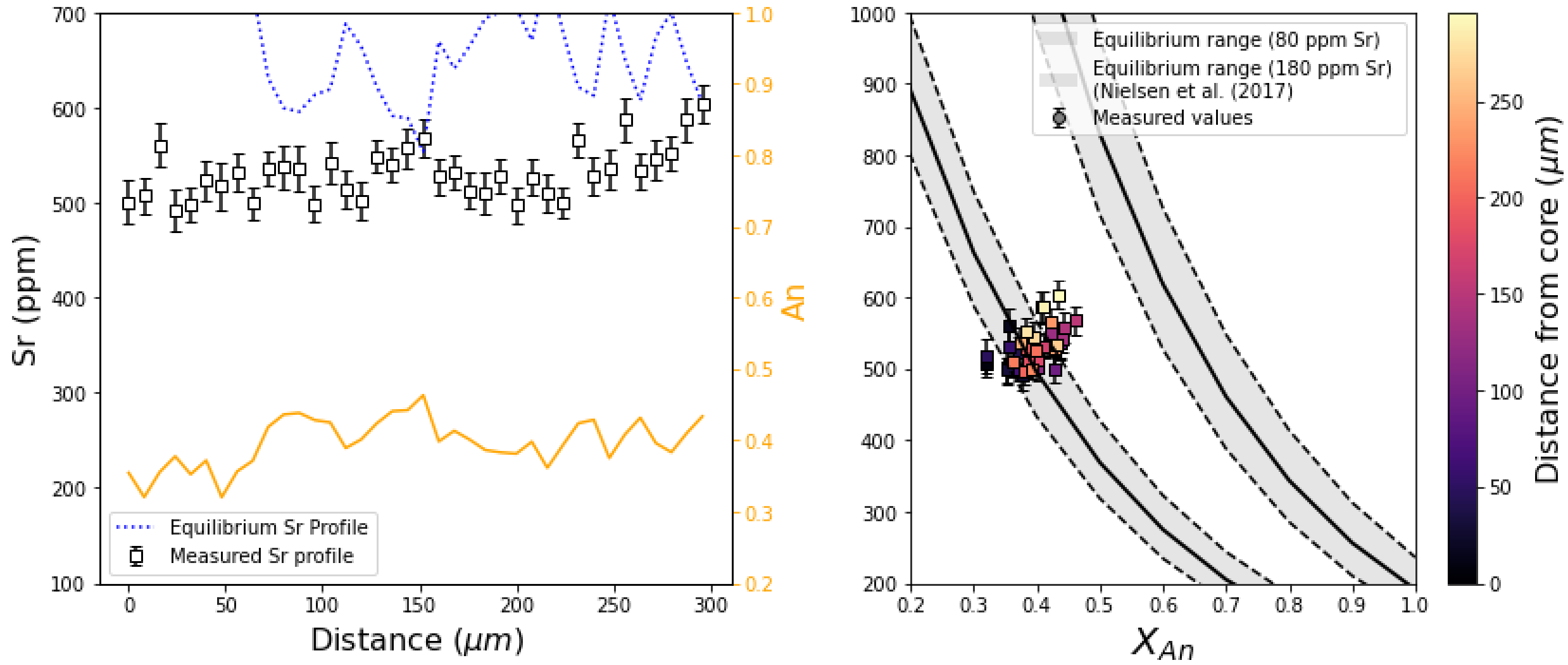

OS-B3-01_Plag-1_RIM-1

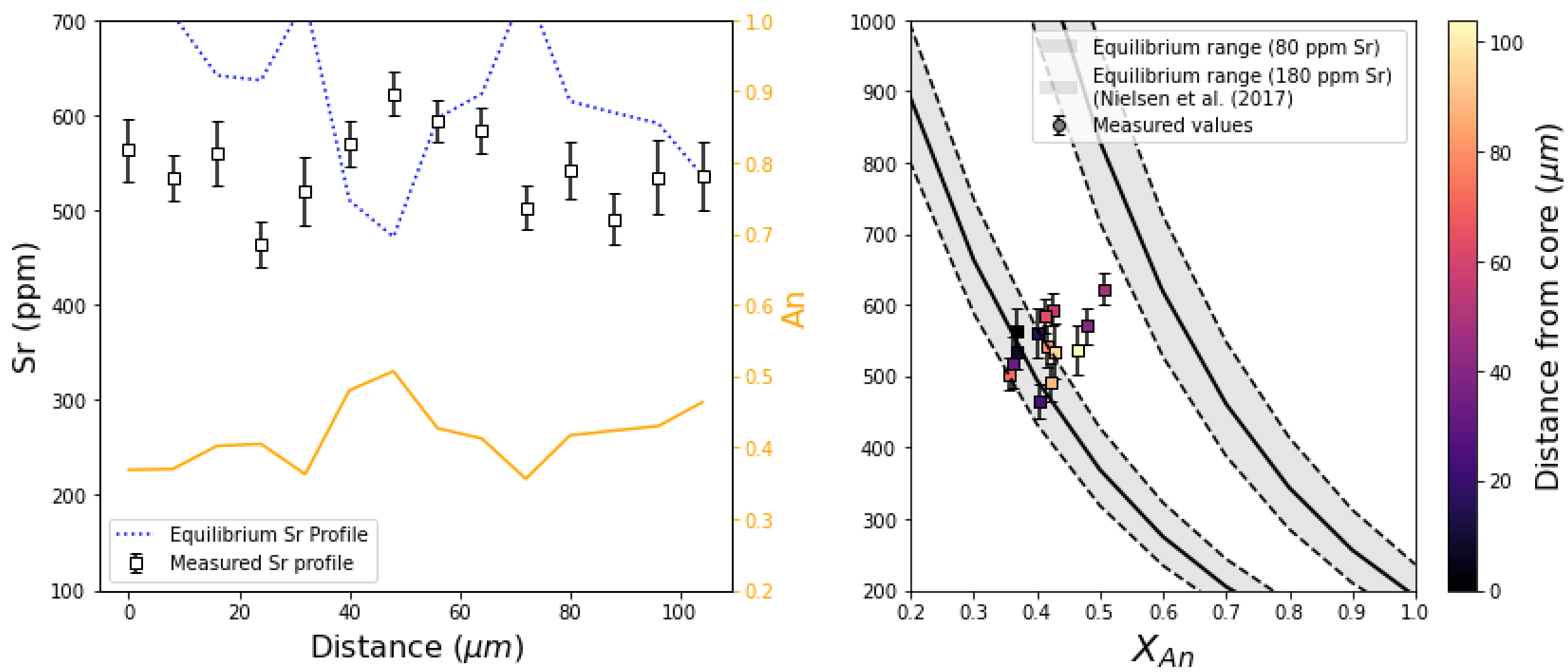

OS-B3-01_Plag-4_RIM-1

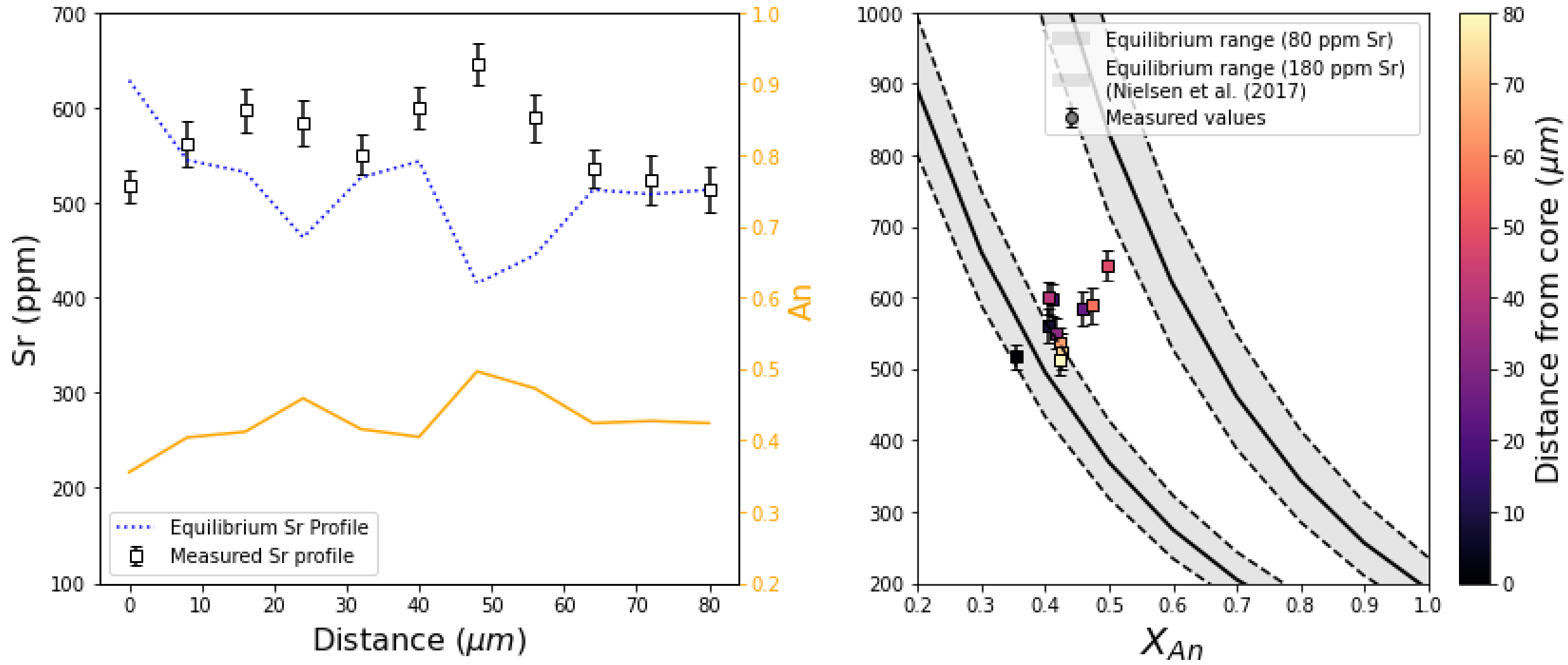

OS-B3-01_Plag-4_RIM-2

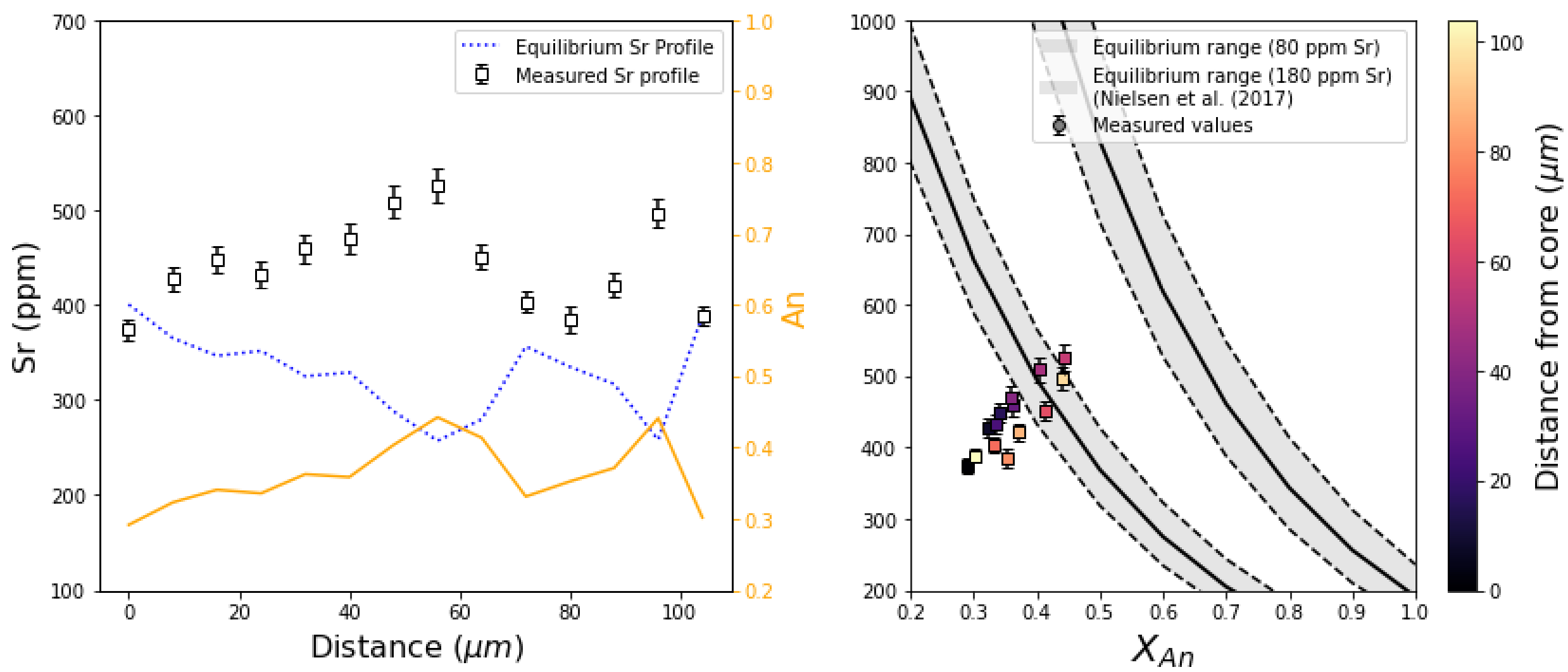

OS-B3-01_Plag-7_RIM-1

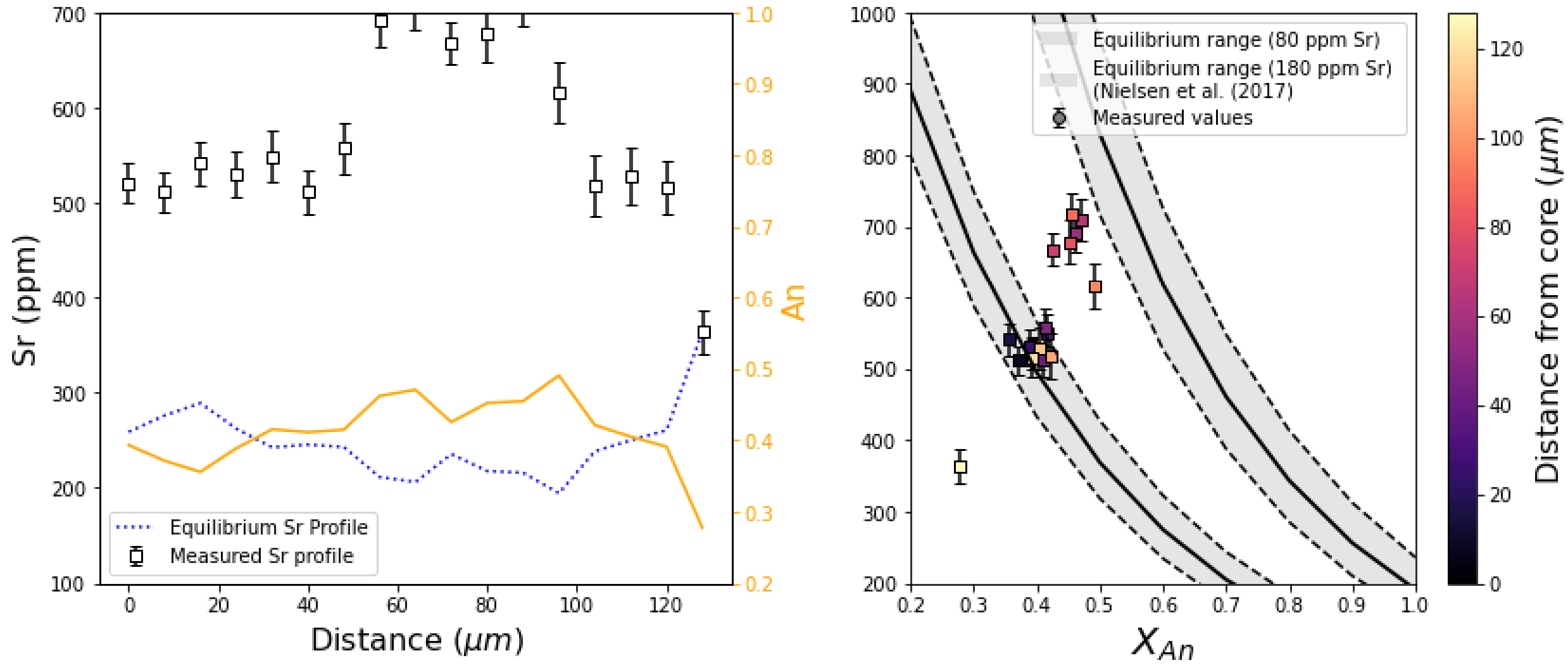

OS-B3-01_Plag-7_RIM-2

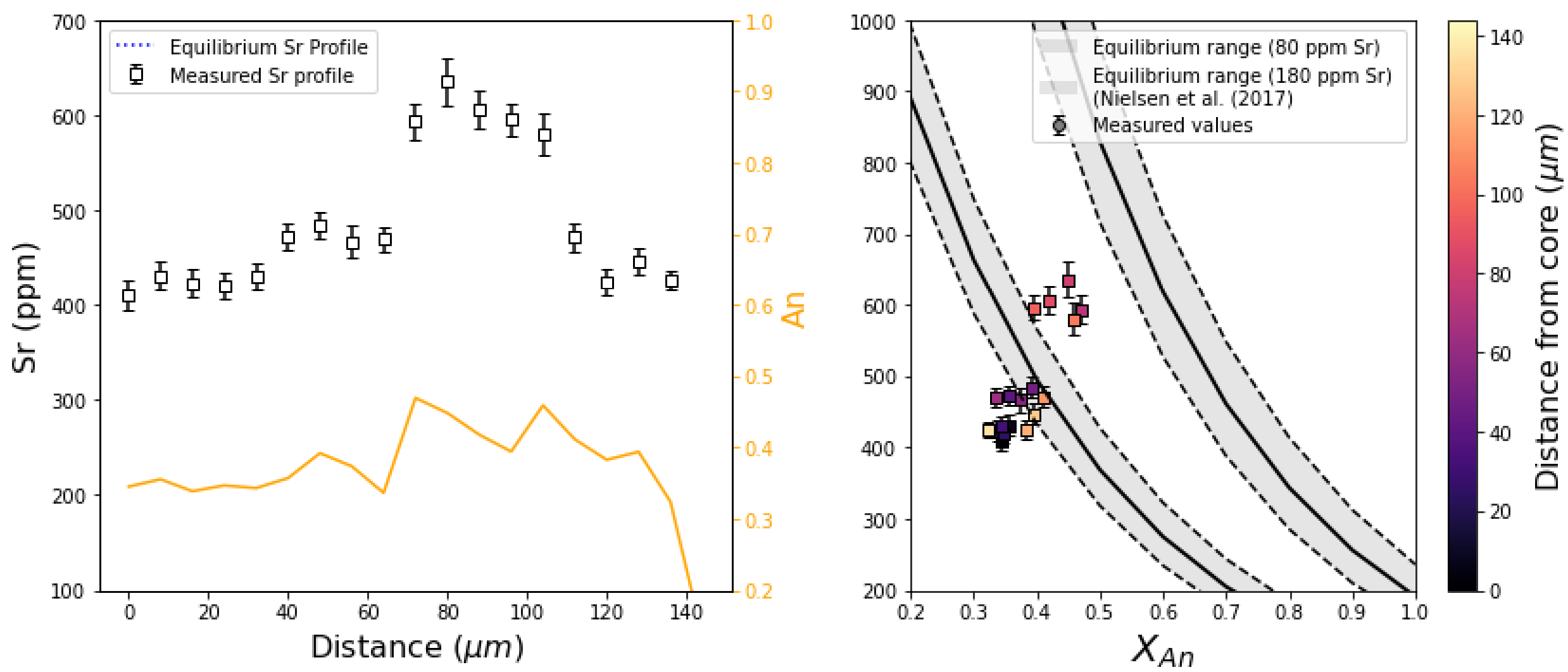

K-B-01_Plag-5_RIM-1

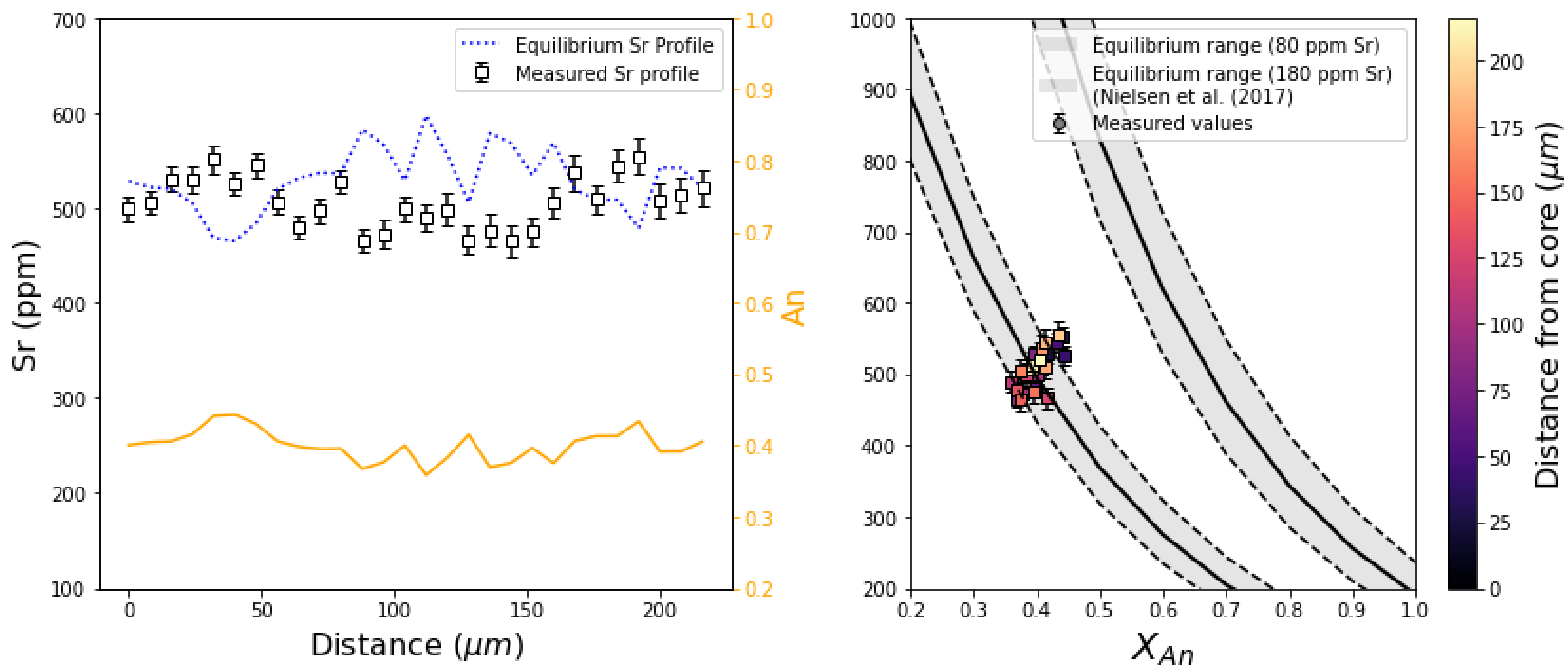

K-B-01_Plag-9_RIM-1

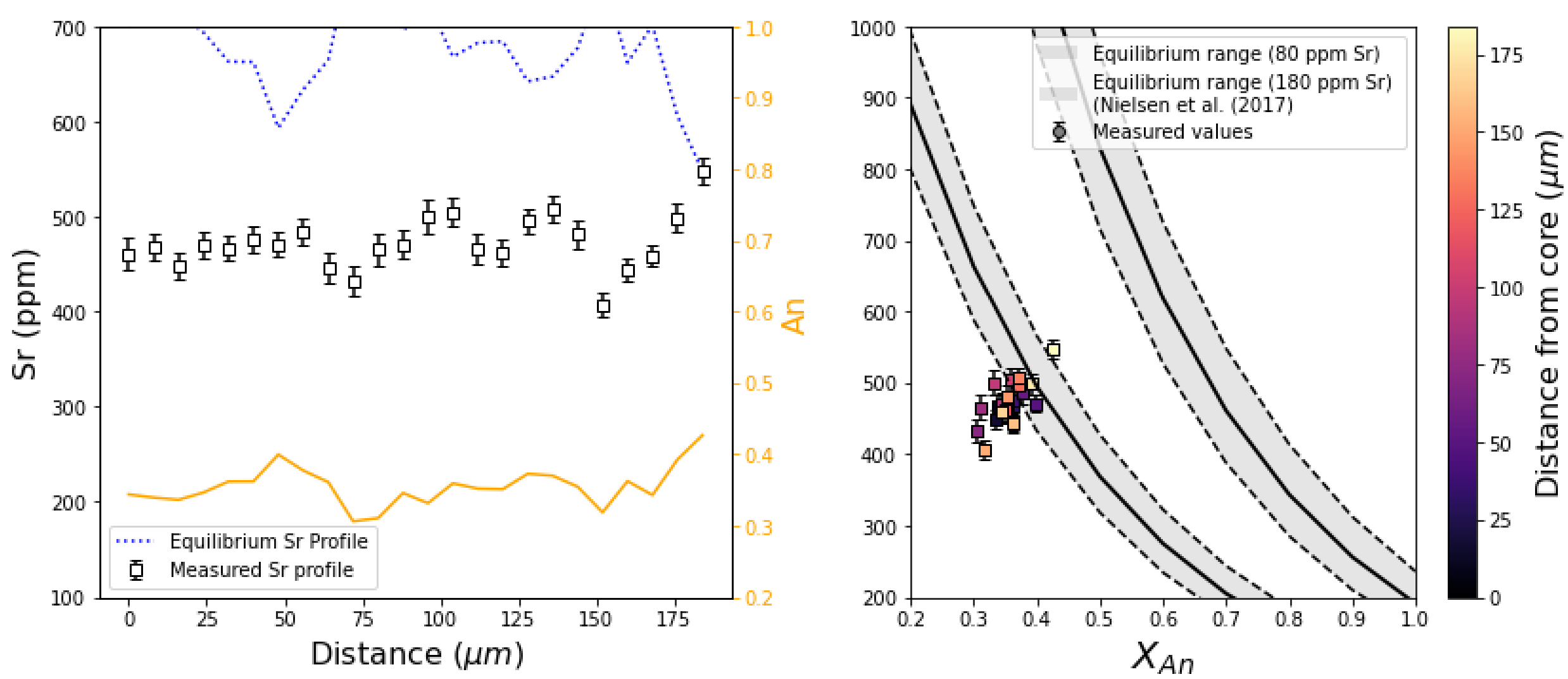

F-A2-01_Plag-8_RIM-1

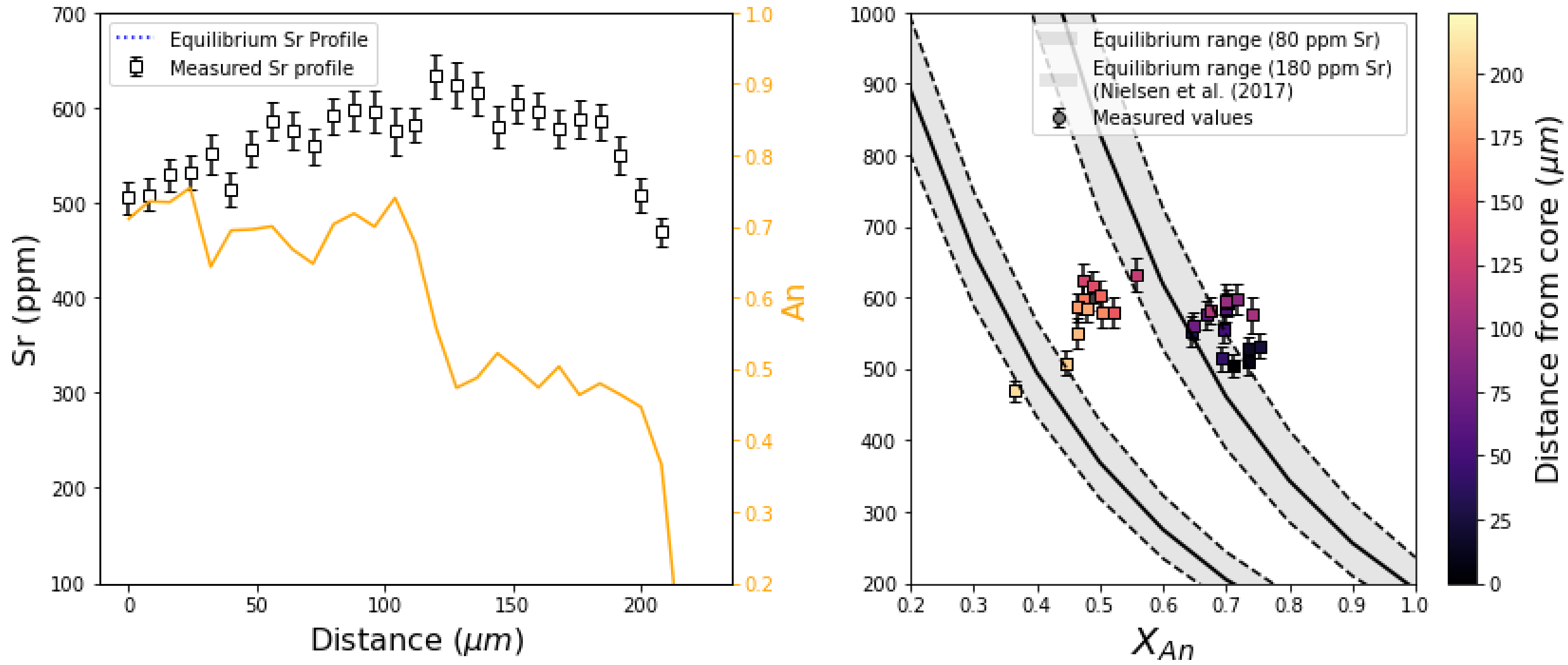

F-A2-01_Plag-10_RIM-1

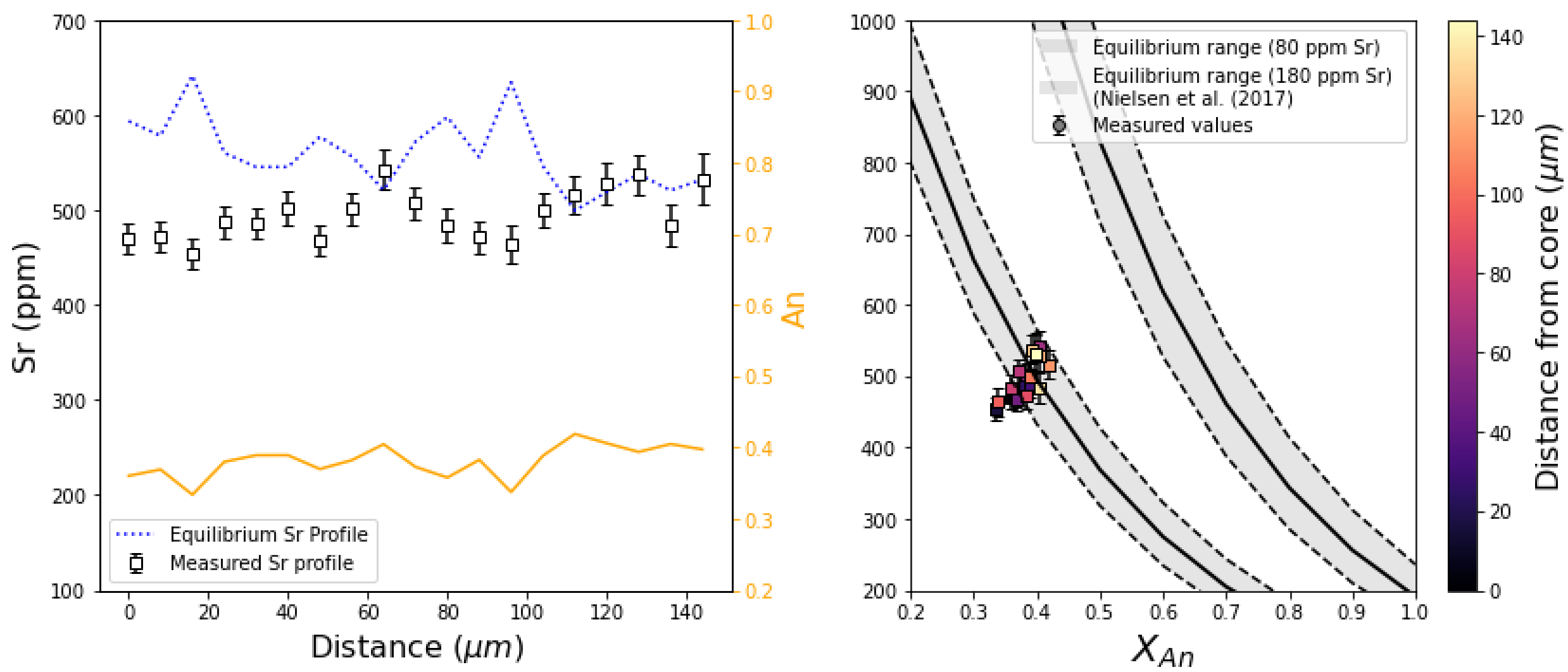

F-A2-01_Plag-11_RIM-1

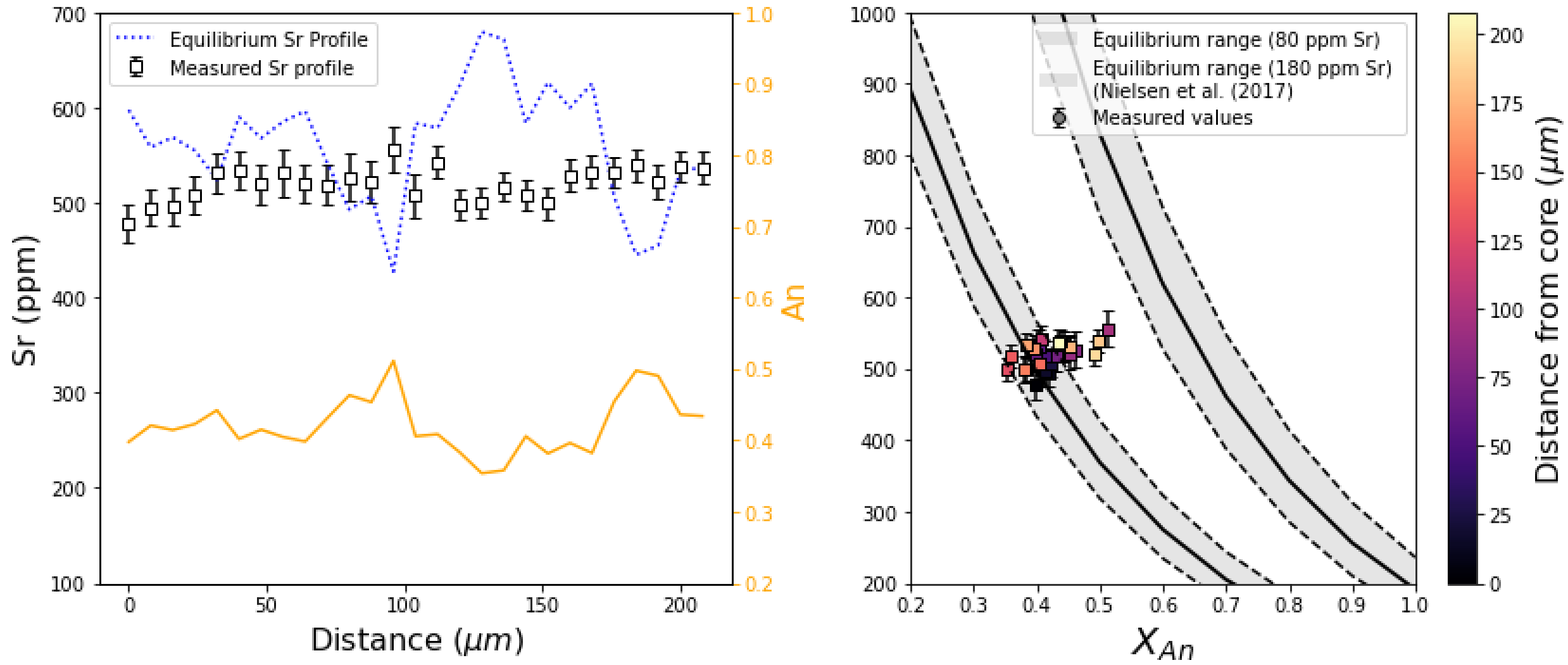

F-A2-01_Plag-16_RIM-1

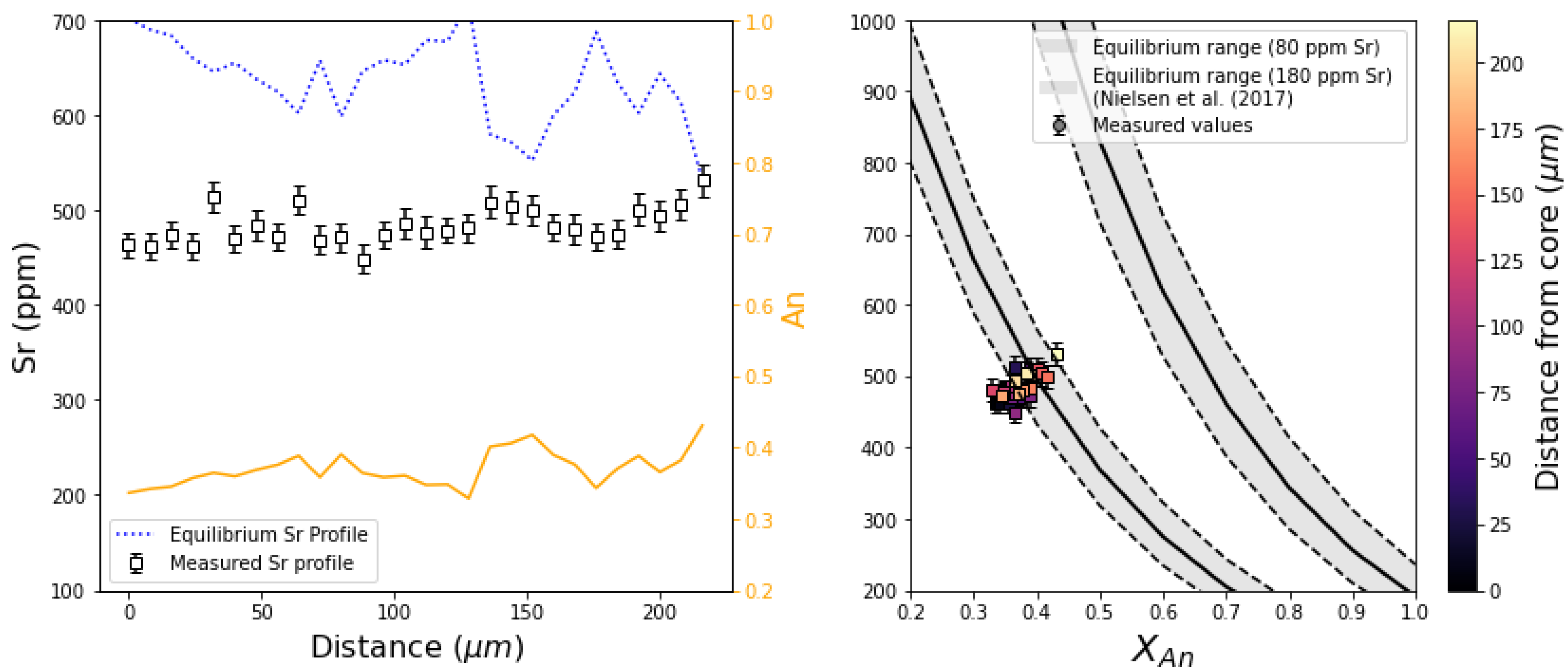

F-A2-02_Plag-3_RIM-1

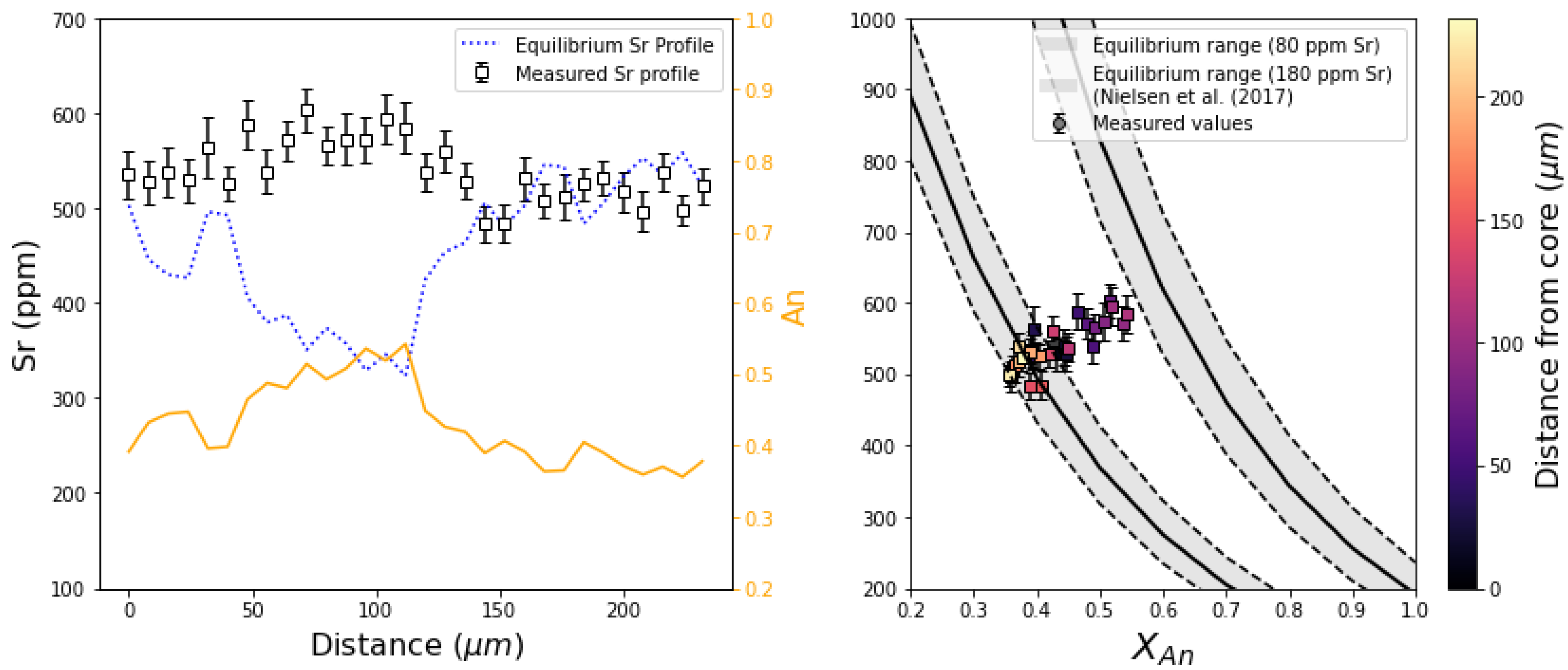

F-A2-02_Plag-7_RIM-1

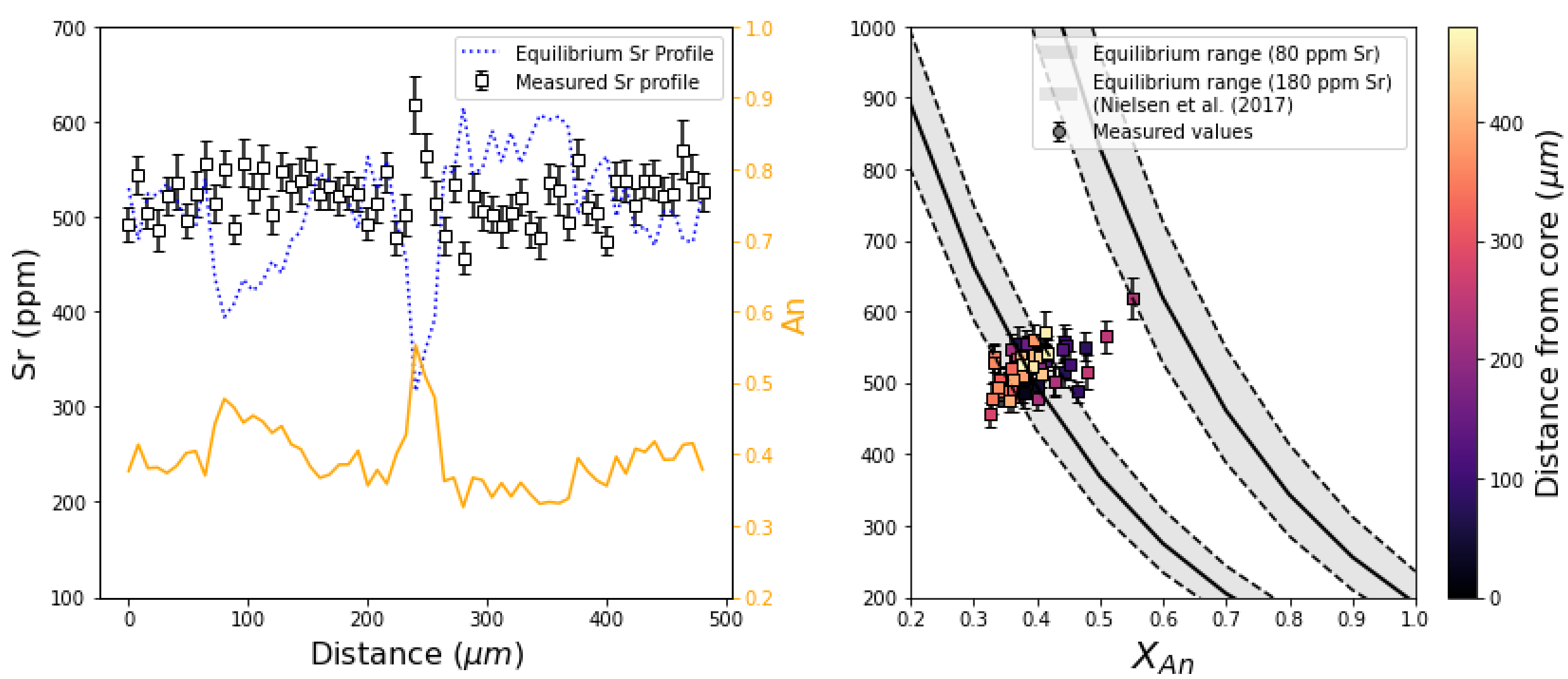

F-A2-02_Plag-9_RIM-1

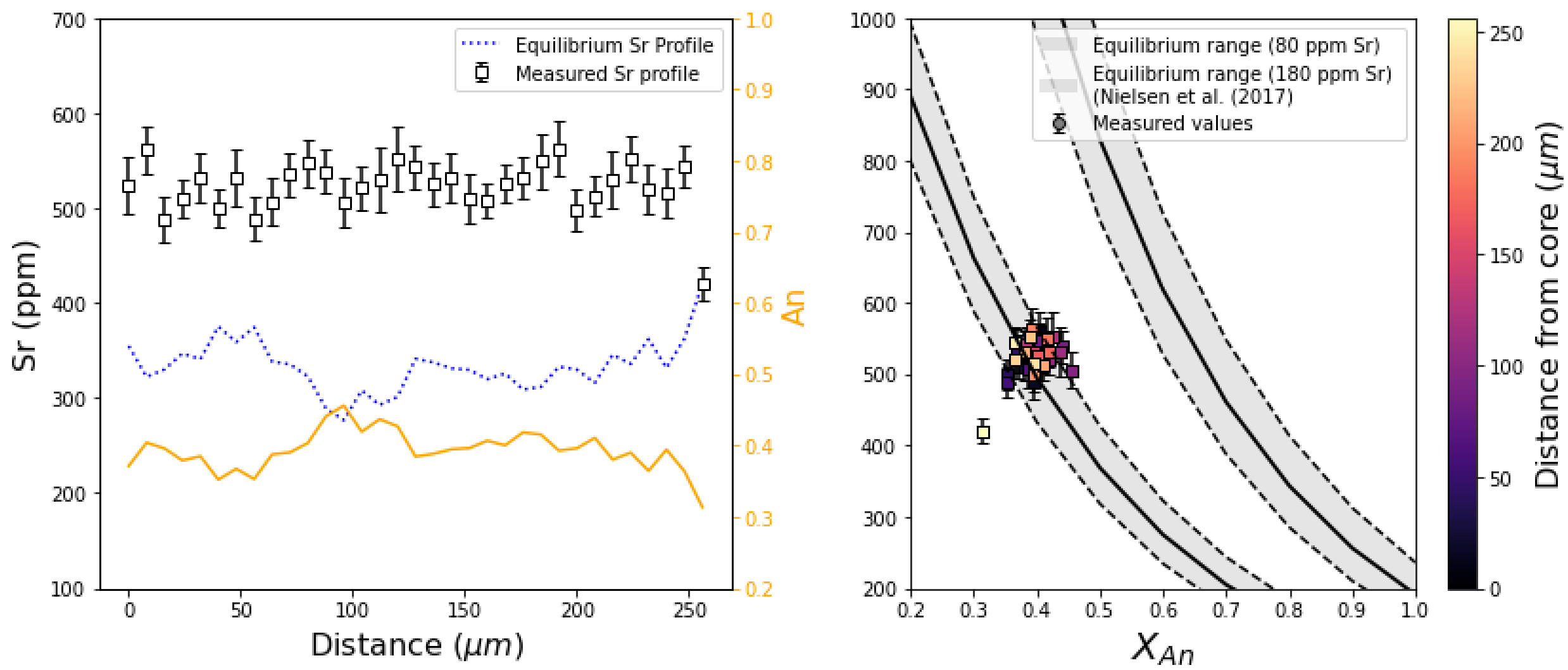

A-03_Plag-5_RIM-1

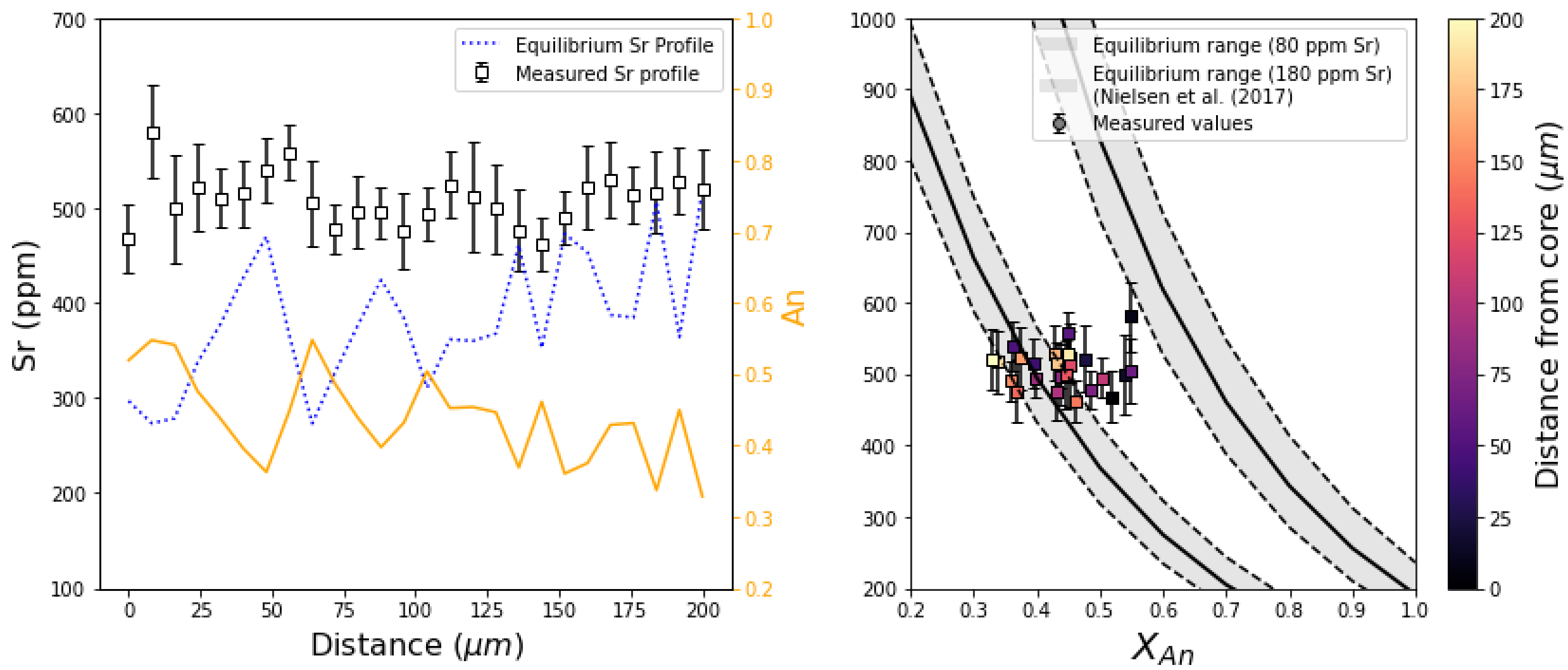

A-03_Plag-6_RIM-1

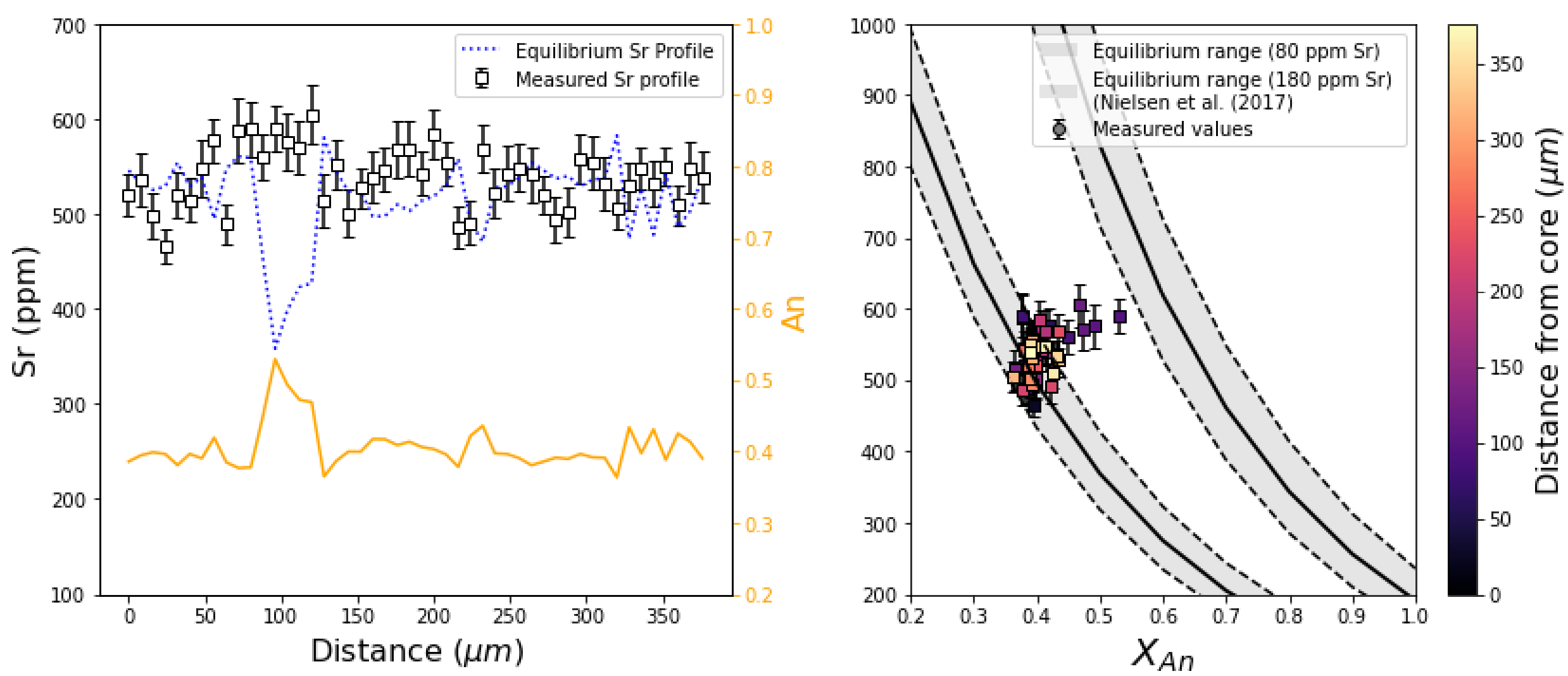

A-03_Plag-11_RIM-2

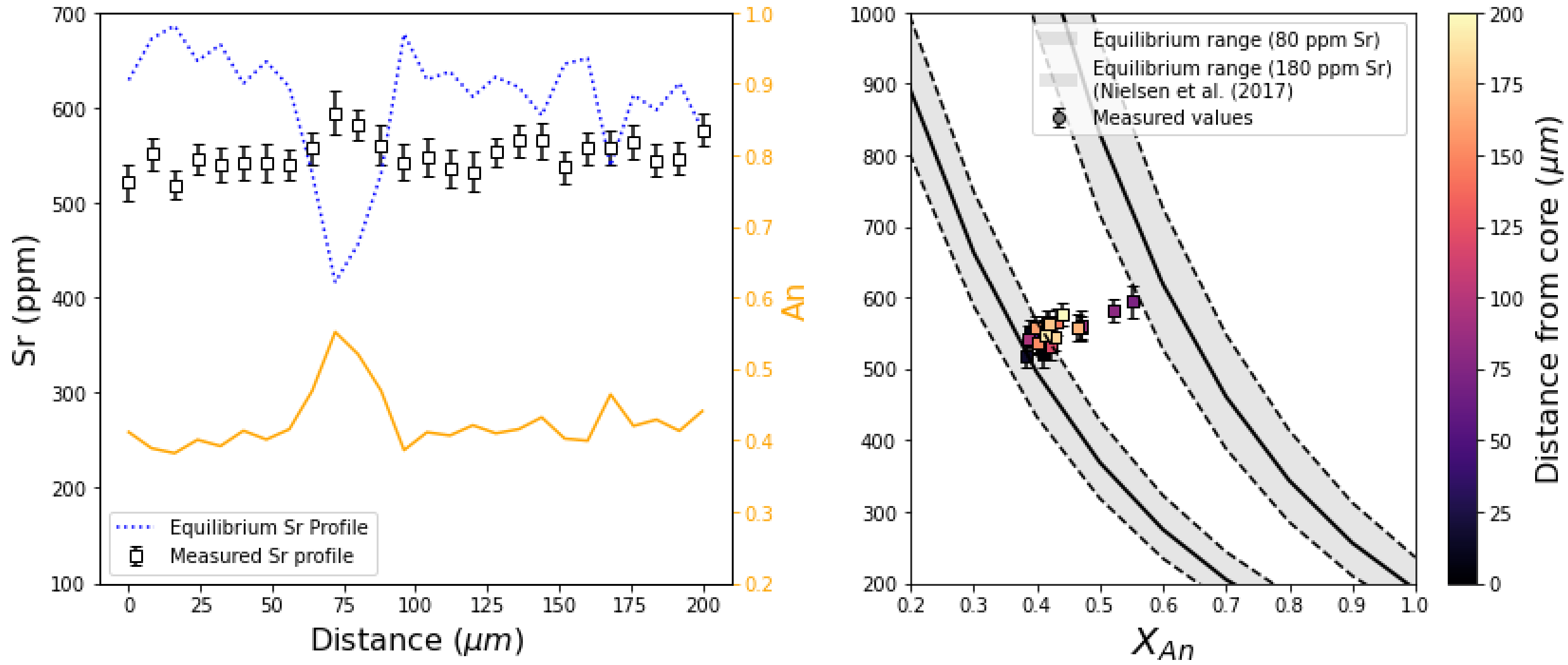

A-08_Plag-4_RIM-1

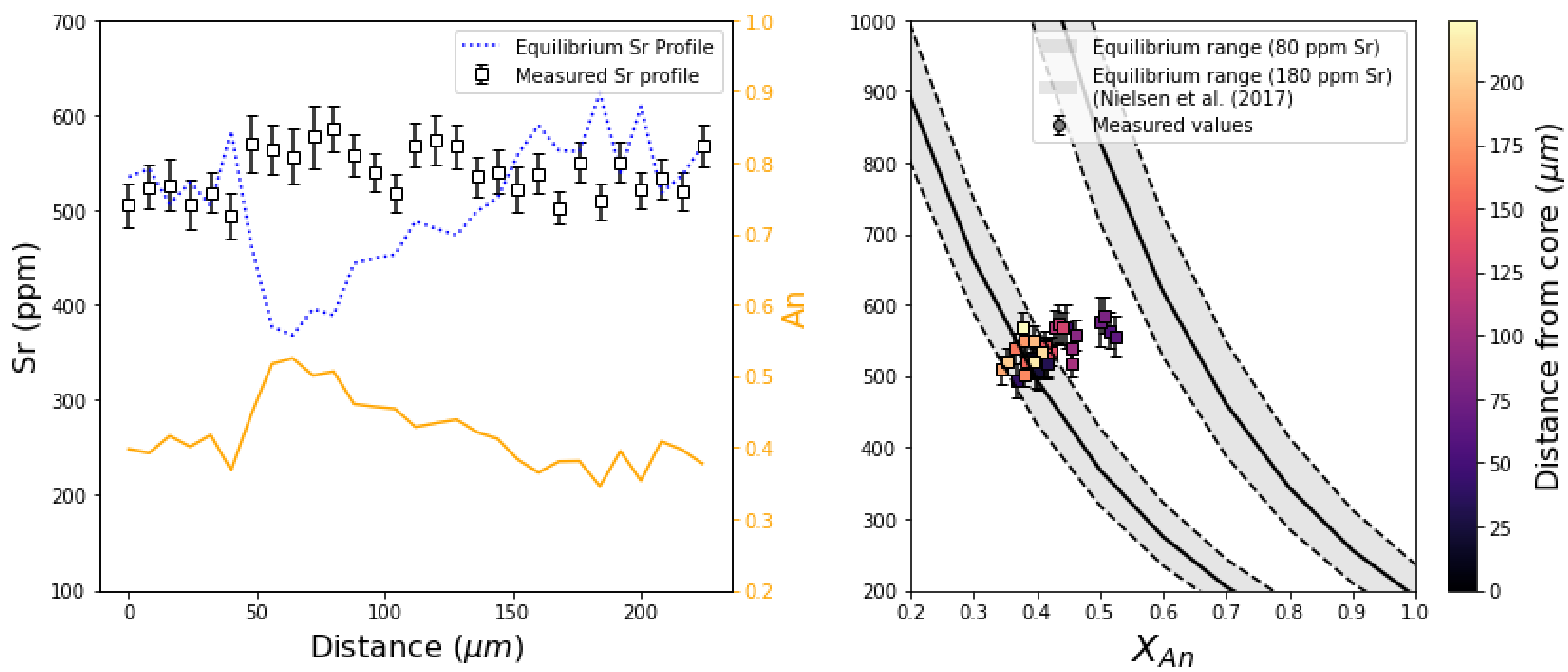

A-10_Plag-4_RIM-2

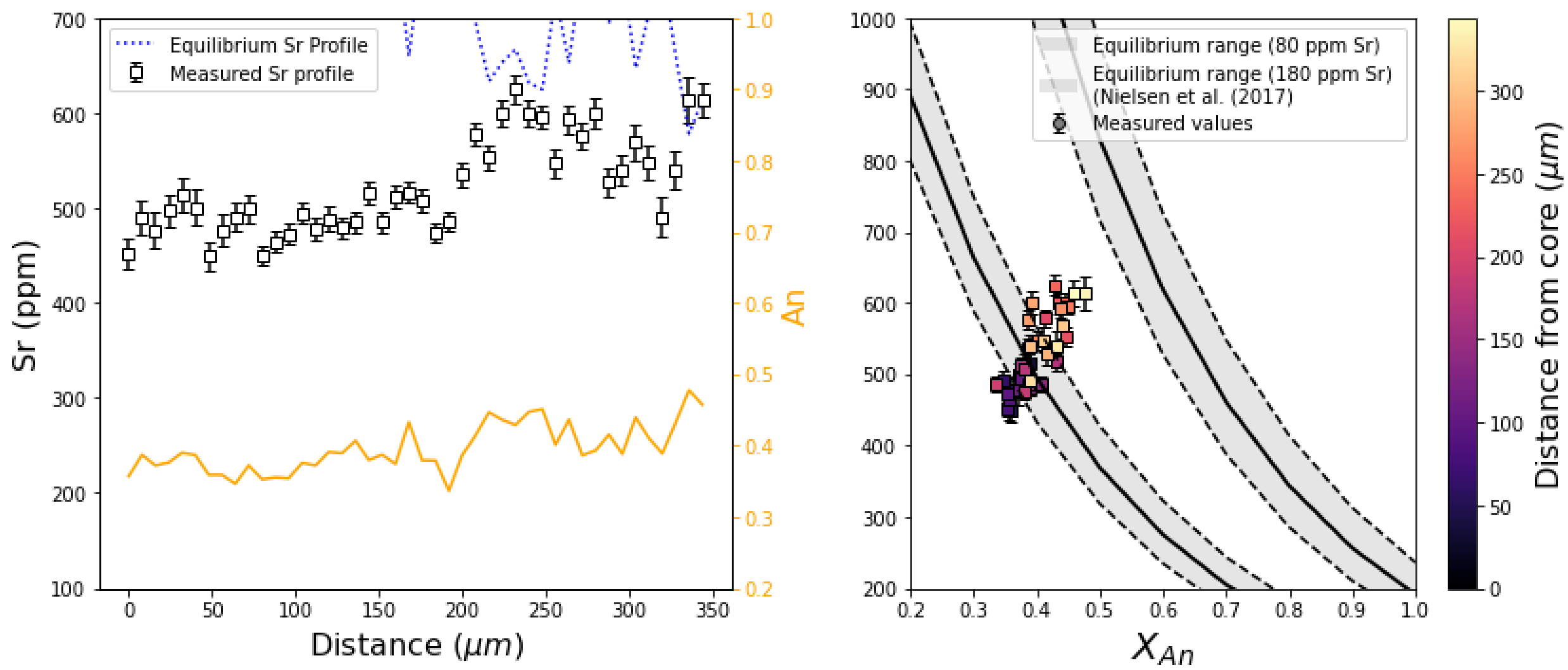

A-10_Plag-8_RIM-2

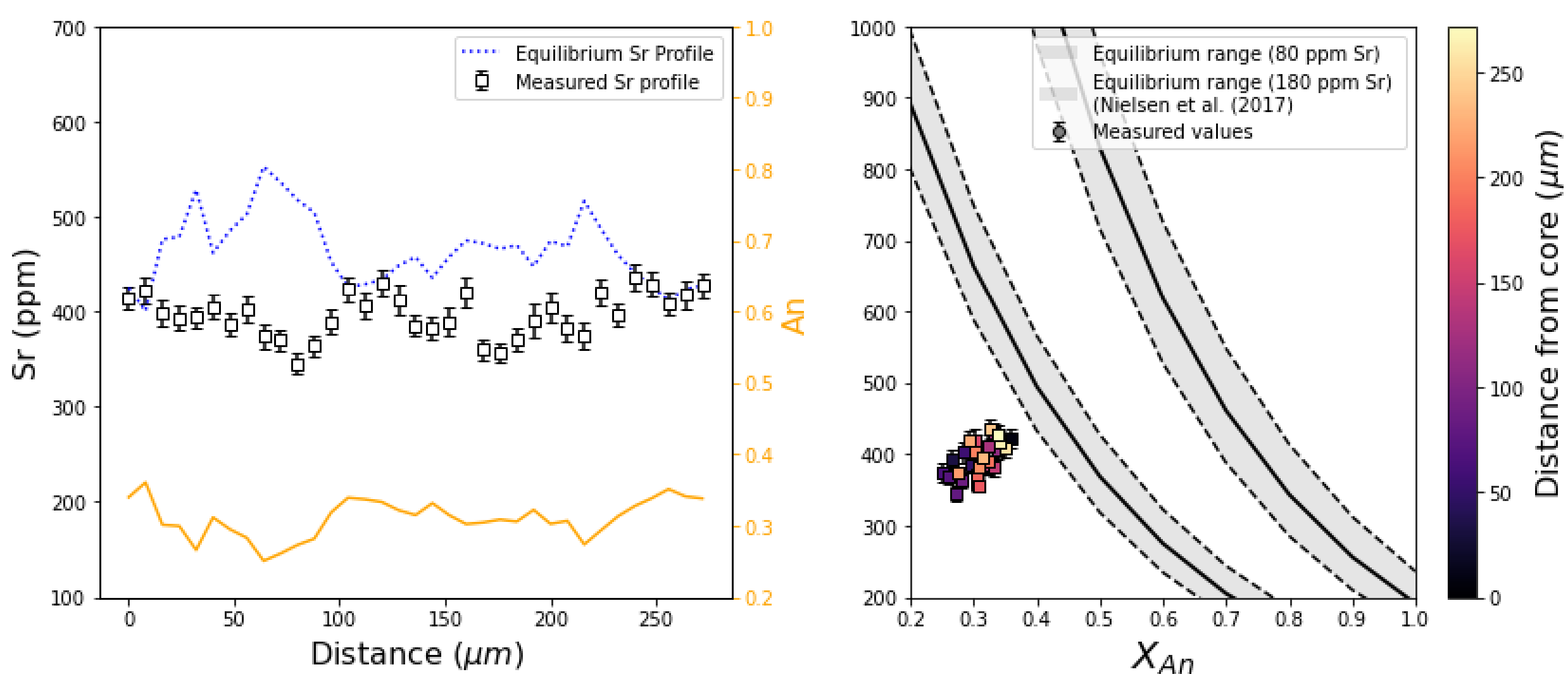

REFERENCES

1. R. A. Daly, The nature of volcanic action. *Proc. Am. Acad. Arts Sci.* **47**, 47–112 (1911).

2. G. R. Davies, A. N. Halliday, Development of the Long Valley rhyolitic magma system: Strontium and neodymium isotope evidence from glasses and individual phenocrysts. *Geochim. Cosmochim. Acta* **62**, 3561–3574 (1998).

3. K. V. Cashman, R. S. J. Sparks, J. D. Blundy, Vertically extensive and unstable magmatic systems: A unified view of igneous processes. *Science* **355**, eaag3055 (2017).

4. A. S. Pamukçu, K. A. Wright, G. A. R. Gualda, D. Gravley, Magma residence and eruption at the Taupo Volcanic Center (Taupo Volcanic Zone, New Zealand): Insights from rhyolite-MELTS geobarometry, diffusion chronometry, and crystal textures. *Contrib. Mineral. Petrol.* **175**, 48 (2020).

5. M. L. Myers, P. J. Wallace, C. J. N. Wilson, B. K. Morter, E. J. Swallow, Prolonged ascent and episodic venting of discrete magma batches at the onset of the Huckleberry Ridge supereruption, Yellowstone. *Earth Planet. Sci. Lett.* **451**, 285–297 (2016).

6. O. Bachmann, C. Huber, Silicic magma reservoirs in the Earth's crust. *Am. Mineral.* **101**, 2377–2404 (2016).

7. O. Bachmann, G. W. Bergantz, On the origin of crystal-poor rhyolites: Extracted from batholithic crystal mushes. *J. Petrol.* **45**, 1565–1582 (2004).

8. W. Hildreth, C. J. N. Wilson, Compositional zoning of the Bishop Tuff. *J. Petrol.* **48**, 951–999 (2007).

9. N. E. Matthews, C. Huber, D. M. Pyle, V. C. Smith, Timescales of magma recharge and reactivation of large silicic systems from ti diffusion in quartz. *J. Petrol.* **53**, 1385–1416 (2012).

10. A. S. Pamukcu, G. A. R. Gualda, F. Bégué, D. M. Gravley, Melt inclusion shapes: Timekeepers of short-lived giant magma bodies. *Geology* **43**, 947–950 (2015).

11. G. A. R. Gualda, A. S. Pamukcu, M. S. Ghiorso, A. T. Anderson, S. R. Sutton, M. L. Rivers, Timescales of quartz crystallization and the longevity of the Bishop giant magma body. *PLOS ONE* **7**, e37492 (2012).

12. K. J. Chamberlain, D. J. Morgan, C. J. N. Wilson, Timescales of mixing and mobilisation in the Bishop Tuff magma body: Perspectives from diffusion chronometry. *Contrib. Mineral. Petrol.* **168**, 1034 (2014).

13. B. W. Pitcher, G. A. R. Gualda, T. Hasegawa, Repetitive duality of rhyolite compositions, timescales, and storage and extraction conditions for pleistocene caldera-forming eruptions, Hokkaido, Japan. *J. Petrol.* **62**, egaa106 (2021).

14. K. M. Cooper, A. J. R. Kent, Rapid remobilization of magmatic crystals kept in cold storage. *Nature* **506**, 480–483 (2014).

15. C. C. Lundstrom, A. F. Glazner, Silicic magmatism and the volcanic–plutonic connection. *Elements* **12**, 91–96 (2016).

16. M. Paulatto, E. E. E. Hooft, K. Chrapkiewicz, B. Heath, D. R. Toomey, J. V. Morgan, Advances in seismic imaging of magma and crystal mush. *Front. Earth Sci.* **10**, 970131 (2022).

17. R. Maguire, B. Schmandt, J. Li, C. Jiang, G. Li, J. Wilgus, M. Chen, Magma accumulation at depths of prior rhyolite storage beneath Yellowstone Caldera. *Science* **378**, 1001–1004 (2022).

18. D. J. Cherniak, E. B. Watson, D. A. Wark, Ti diffusion in quartz. *Chem. Geol.* **236**, 65–74 (2007).

19. A. Audétat, N. Miyajima, D. Wiesner, J. N. Audinot, Confirmation of slow Ti diffusion in quartz by diffusion couple experiments and evidence from natural samples. *Geology* **49**, 963–967 (2021).

20. M. C. Jollands, E. Bloch, O. Müntener, New Ti-in-quartz diffusivities reconcile natural Ti zoning with time scales and temperatures of upper crustal magma reservoirs. *Geology* **48**, 654–657 (2020).

21. A. Audétat, A. K. Schmitt, R. Njul, M. Saalfeld, A. Borisova, Y. Lu, New constraints on Ti diffusion in quartz and the priming of silicic volcanic eruptions. *Nat. Commun.* **14**, 4277 (2023).

22. K. M. Cooper, Timescales of crustal magma reservoir processes: Insights from U-series crystal ages. *Geol. Soc. Spec. Publ.* **422**, 141–174 (2015).

23. N. Geshi, I. Yamada, K. Matsumoto, A. Nishihara, I. Miyagi, Accumulation of rhyolite magma and triggers for a caldera-forming eruption of the Aira Caldera, Japan, *Bull. Volcanol.* **82**, 44 (2020).

24. S. Nagaoka, The late Quaternary tephra layers from the caldera volcanoes in and around Kagoshima Bay, southern Kyushu, Japan. *Geogr. Rep. Tokyo Metrop. Univ.* **23**, 49–122 (1988).

25. V. C. Smith, R. A. Staff, S. P. E. Blockley, C. Bronk Ramsey, T. Nakagawa, D. F. Mark, K. Takemura, T. Danhara, Identification and correlation of visible tephras in the Lake Suigetsu SG06 sedimentary archive, Japan: Chronostratigraphic markers for synchronising of east Asian/west Pacific palaeoclimatic records across the last 150 ka. *Quat. Sci. Rev.* **67**, 121–137 (2013).

26. N. Geshi, Y. Miyabuchi, Conduit enlargement during the precursory Plinian eruption of Aira Caldera, Japan. *Bull. Volcanol.* **78**, 63 (2016).

27. S. Nagaoka, M. Okuno, F. Arai, Tephrostratigraphy and eruptive history of the Aira caldera volcano during 100-30ka, Kyushu, Japan. *J. Geol. Soc. Japan* **107**, 432–450 (2001).

28. T. Kobayashi, T. Tameike, History of eruptions and volcanic damage from Sakurajima Volcano, Southern Kyushu, Japan. *Quat. Res.* **41**, 269–278 (2002).

29. T. Yamasaki, F. Sigmundsson, M. Iguchi, Viscoelastic crustal response to magma supply and discharge in the upper crust: Implications for the uplift of the Aira caldera before and after the 1914 eruption of the Sakurajima volcano. *Earth Planet. Sci. Lett.* **531**, 115981 (2020).

30. G. A. R. Gualda, M. S. Ghiorso, Phase-equilibrium geobarometers for silicic rocks based on rhyolite-MELTS. Part 1: Principles, procedures, and evaluation of the method. *Contrib. Mineral. Petrol.* **168**, 1033 (2014).

31. M. S. Ghiorso, G. A. R. Gualda, An H_2O–CO_2 mixed fluid saturation model compatible with rhyolite-MELTS. *Contrib. Mineral. Petrol.* **169**, 53 (2015).

32. G. A. R. Gualda, M. S. Ghiorso, R. V. Lemons, T. L. Carley, Rhyolite-MELTS: A modified calibration of MELTS optimized for silica-rich, fluid-bearing magmatic systems. *J. Petrol.* **53**, 875–890 (2012).

33. S. Newman, J. B. Lowenstern, VolatileCalc: A silicate melt–H_2O–CO_2 solution model written in Visual Basic for excel. *Comput. Geosci.* **28**, 597–604 (2002).

34. E. B. Watson, T. M. Harrison, Zircon saturation revisited: Temperature and composition effects in a variety of crustal magma types. *Earth Planet. Sci. Lett.* **64**, 295–304 (1983).

35. P. Boehnke, E. B. Watson, D. Trail, T. M. Harrison, A. K. Schmitt, Zircon saturation re-revisited. *Chem. Geol.* **351**, 324–334 (2013).

36. F. Gervasoni, S. Klemme, E. R. V. Rocha-Júnior, J. Berndt, Zircon saturation in silicate melts: A new and improved model for aluminous and alkaline melts. *Contrib. Mineral. Petrol.* **171**, 21 (2016).

37. W. P. Leeman, C. M. MacRae, N. C. Wilson, A. Torpy, C. T. A. Lee, J. J. Student, J. B. Thomas, E. P. Vicenzi, A study of cathodoluminescence and trace element compositional zoning in natural quartz from volcanic rocks: Mapping titanium content in quartz. *Microsc. Microanal.* **18**, 1322–1341 (2012).

38. G. A. R. Gualda, S. R. Sutton, The year leading to a supereruption. *PLOS ONE* **11**, e0159200 (2016).

39. S. Y. Wang, G. A. R. Gualda, J. Lubbers, A. J. R. Kent, Fast or slow: An evaluation of Ti-in-quartz diffusion coefficients through comparisons of quartz and plagioclase diffusion times. *Volcanica* **8**, 189–202 (2025).

40. A. Nishihara, Y. Tatsumi, K. Kaneko, J.-I. Kimura, Q. Chang, N. Geshi, T. Miyazaki, B. S. Vaglarov, H. Hinata, K. Suzuki-Kamata, Voluminous magma formation for the 30-ka Aira caldera-forming eruption in SW Japan: Contributions of crust-derived felsic and mafic magmas. *Front. Earth Sci.* **11**, 1283844 (2024).

41. T. D. Schlieder, K. M. Cooper, A. J. R. Kent, R. Bradshaw, C. Huber, Thermal storage conditions and origin of compositional diversity of plagioclase crystals in magmas from the 1980 and 2004-2005 eruptions of Mount Saint Helens. *J. Petrol.* **63**, egac064 (2022).

42. G. N. Fabbro, T. H. Druitt, F. Costa, Storage and eruption of silicic magma across the transition from dominantly effusive to caldera-forming states at an arc volcano (Santorini, Greece). *J. Petrol.* **58**, 2429–2464 (2017).

43. J. Lubbers, A. J. R. Kent, S. de Silva, Thermal budgets of magma storage constrained by diffusion chronometry: The Cerro Galán Ignimbrite. *J. Petrol.* **63**, egac048 (2022).

44. J. Lubbers, A. J. R. Kent, S. de Silva, Constraining magma storage conditions of the Toba magmatic system: A plagioclase and amphibole perspective. *Contrib. Mineral. Petrol.* **179**, 12 (2024).

45. I. N. Bindeman, A. M. Davis, M. J. Drake, Ion microprobe study of plagioclase-basalt partition experiments at natural concentration levels of trace elements. *Geochim. Cosmochim. Acta* **62**, 1175–1193 (1998).

46. R. Dohmen, J. Blundy, A predictive thermodynamic model for element partitioning between plagioclase and melt as a function of pressure, temperature and composition. *Am. J. Sci.* **314**, 1319–1372 (2014).

47. R. L. Nielsen, G. Ustunisik, A. B. Weinsteiger, F. J. Tepley, A. D. Johnston, A. J. R. Kent, Trace element partitioning between plagioclase and melt: An investigation of the impact of experimental and analytical procedures. *Geochem. Geophys. Geosyst.* **18**, 3359–3384 (2017).

48. T. Grocolas, E. M. Bloch, A. S. Bouvier, O. Müntener, Diffusion of Sr and Ba in plagioclase: Composition and silica activity dependencies, and application to volcanic rocks. *Earth Planet. Sci. Lett.* **651**, 119141 (2025).

49. T. Kuritani, Geochemical constraints on the evolution of the magmatic system leading to catastrophic eruptions at Aira Caldera, Japan. *Lithos* **450–451**, 107208 (2023).

50. N. J. G. Pearce, J. A. Westgate, G. A. R. Gualda, E. Gatti, R. F. Muhammad, Tephra glass chemistry provides storage and discharge details of five magma reservoirs which fed the 75 ka Youngest Toba Tuff eruption, northern Sumatra. *J. Quat. Sci.* **35**, 256–271 (2020).

51. G. F. Cooper, C. J. N. Wilson, M.-A. Millet, J. A. Baker, E. G. C. Smith, Systematic tapping of independent magma chambers during the 1 Ma Kidnappers supereruption. *Earth Planet. Sci. Lett.* **313–314**, 23–33 (2012).

52. A. Barth, T. Plank, The ins and outs of water in olivine-hosted melt inclusions: Hygrometer vs speedometer. *Front. Earth Sci.* **9**, 343 (2021).

53. C. R. Tierney, M. R. Reid, Quartz-hosted melt inclusions as windows into magma assembly and storage processes in the Youngest Toba Tuff. *Geochem. Geophys. Geosyst.* **22**, e2020GC009564 (2021).

54. K. J. Chamberlain, C. J. N. Wilson, P. J. Wallace, M.-A. Millet, Micro-analytical perspectives on the Bishop Tuff and its magma chamber. *J. Petrol.* **56**, 605–640 (2015).

55. A. Yasuda, M. Yoshimoto, T. Fujii, The depth of a magma chamber associated with the Aira caldera formation. *Bull. Volcanol. Soc. Jpn.* **60**, 381–397 (2015).

56. H. Miyamachi, H. Yakiwara, R. Kobayashi, S. Hirano, T. Kubo, M. Souda, K. Sakao, N. Unno, T. Matsushima, K. Uchida, R. Miyamachi, K. Isoda, Y. Teguri, Y. Kamiya, A. Triahadini, H.

Shimizu, H. Katao, T. Shibutani, T. Tameguri, Y. Yamashita, T. Miura, J. Nakagawa, I. Yoneda, S. Kato, K. Takishita, K. Nakai, Y. Maeda, T. Watanabe, S. Horikawa, K. Matsushiro, T. Okuda, S. Tsuji, N. Sogawa, D. Hasegawa, K. Nakahigashi, E. Kurashimo, T. Yamada, H. Abe, M. Ando, S. Tanaka, S. Ikezawa, T. Iwasaki, M. Shinohara, T. Sato, M. Yamamoto, R. Azuma, S. Hirahara, T. Nakayama, S. Suzuki, S. Otomo, R. Hino, T. Tsutsui, Y. Inoue, R. Takei, Y. Tada, H. Takahashi, Y. Murai, H. Aoyama, M. Ohzono, T. Shiina, M. Takada, M. Ichiyanagi, T. Yamaguchi, N. Ono, K. Saito, C. Ito, Y. Susukida, T. Nakagaki, Y. Tanaka, Y. Akinaga, Solidified magma reservoir derived from active source seismic experiments in the Aira caldera, southern Kyushu, Japan. *Earth Planets Space* **75**, 166 (2023).

57. J. Blundy, K. Cashman, Ascent-driven crystallisation of dacite magmas at Mount St Helens, 1980-1986. *Contrib. Mineral. Petrol.* **140**, 631–650 (2001).

58. G. A. R. Gualda, M. S. Ghiorso, Low-pressure origin of high-silica rhyolites and granites. *J. Geol.* **121**, 537–545 (2013).

59. B. J. Giletti, J. E. D. Casserly, Strontium diffusion kinetics in plagioclase feldspars. *Geochim. Cosmochim. Acta* **58**, 3785–3793 (1994).

60. C. Ginibre, G. Wörner, Variable parent magmas and recharge regimes of the Parinacota magma system (N. Chile) revealed by Fe, Mg and Sr zoning in plagioclase. *Lithos* **98**, 118–140 (2007).

61. M. J. Streck, Mineral textures and zoning as evidence for open system processes. *Rev. Mineral. Geochem.* **69**, 595–622 (2008).

62. J. R. Boro, J. A. Wolff, O. K. Neill, A. R. Steiner, F. C. Ramos, Titanium diffusion profiles and melt inclusion chemistry and morphology in quartz from the Tshirege Member of the Bandelier Tuf. *Am. Mineral.* **106**, 620–632 (2021).

63. O. Barbee, C. Chesner, C. Deering, Quartz crystals in Toba rhyolites show textures symptomatic of rapid crystallization. *Am. Mineral.* **105**, 194–226 (2020).

64. L. J. Kelly, G. A. R. Gualda, D. M. Gravley, D. E. Dempsey, Hydrothermal cooling as a requirement for short storage of silicic magmas. *Geochem. Geophys. Geosyst.* **22**, e2021GC009794 (2021).

65. Y. Watanabe, Late Cenozoic evolution of epithermal gold metallogenic provinces in Kyushu, Japan. *Miner. Depos.* **40**, 307–323 (2005).

66. H. I. Shamloo, C. B. Till, Decadal transition from quiescence to supereruption: Petrologic investigation of the Lava Creek Tuff, Yellowstone Caldera, WY. *Contrib. Mineral. Petrol.* **174**, 32 (2019).

67. T. Flaherty, T. H. Druitt, H. Tuffen, M. D. Higgins, F. Costa, A. Cadoux, Multiple timescale constraints for high-flux magma chamber assembly prior to the Late Bronze Age eruption of Santorini (Greece). *Contrib. Mineral. Petrol.* **173**, 75 (2018).

68. S. J. Barker, C. J. N. Wilson, D. J. Morgan, J. V. Rowland, Rapid priming, accumulation, and recharge of magma driving recent eruptions at a hyperactive caldera volcano. *Geology* **44**, 323–326 (2016).

69. A. S. R. Allan, S. J. Barker, M. A. Millet, D. J. Morgan, S. M. Rooyakkers, C. I. Schipper, C. J. N. Wilson, A cascade of magmatic events during the assembly and eruption of a super-sized magma body. *Contrib. Mineral. Petrol.* **172**, 49 (2017).

70. A. S. Pamukçu, S. M. Hickernell, M. P. Eddy, B. Schoene, T. Steiner-Leach, Geology constrains the diffusivity of Ti in quartz and crystallization timescales of high-silica magmas in the Searchlight Magmatic System (NV, USA). *Earth Planet. Sci. Lett.* **666**, 119437 (2025).

71. B. G. Mason, D. M. Pyle, C. Oppenheimer, The size and frequency of the largest explosive eruptions on Earth. *Bull. Volcanol.* **66**, 735–748 (2004).

72. F. J. Spera, W. A. Bohrson, Rejuvenation of crustal magma mush: A tale of multiply nested processes and timescales. *Am. J. Sci.* **318**, 90–140 (2018).

73. J. Cabato, R. Altherr, T. Ludwig, H. P. Meyer, Li, Be, B concentrations and δ7Li values in plagioclase phenocrysts of dacites from Nea Kameni (Santorini, Greece). *Contrib. Mineral. Petrol.* **165**, 1135–1154 (2013).

74. F. Pohl, H. Behrens, M. Oeser, F. Marxer, R. Dohmen, Li diffusion in plagioclase crystals and glasses—Implications for timescales of geological processes. *Eur. J. Mineral.* **36**, 985–1003 (2024).

75. J. Neukampf, O. Laurent, P. Tollan, A. S. Bouvier, T. Magna, P. Ulmer, L. France, B. S. Ellis, O. Bachmann, Degassing from magma reservoir to eruption in silicic systems: The Li elemental and isotopic record from rhyolitic melt inclusions and host quartz in a Yellowstone rhyolite. *Geochim. Cosmochim. Acta* **326**, 56–76 (2022).

76. A. J. R. Kent, J. Blundy, K. V. Cashman, K. M. Copper, C. Donnelly, J. S. Pallister, M. Reagan, M. C. Rowe, C. R. Thornber, Vapor transfer prior to the October 2004 eruption of Mount St. Helens, Washington. *Geology* **35**, 231–234 (2007).

77. J. Blundy, K. V Cashman, K. Berlo, D. R. Sherrod, W. E. Scott, P. H. Stauffer, "Evolving magma storage conditions beneath Mount St. Helens inferred from chemical variations in melt inclusions from the 1980–1986 and current (2004–2006) eruptions" in *A Volcano Rekindled: The Renewed Eruption of Mount St. Helens* (U.S. Geological Survey, 2008), pp. 755–790.

78. M. Myers, R. Spallanzani, D. M. Schwartz, C. Mercer, B. Hosseini, Variable partitioning of lithium in rhyolitic melt during decompression and ascent. *Econ. Geol.* **120**, 1191–1206 (2025).

79. M. Dupont de Dinechin, C. Martel, H. Balcone-Boissard, M. K. Rusiecka, R. Champallier, E. Deloule, Experimental determination of lithium partitioning between plagioclase and hydrous rhyolitic melt. *Chem. Geol.* **695**, 123047 (2025).

80. M. Pistone, J. Blundy, R. A. Brooker, Water transfer during magma mixing events: Insights into crystal mush rejuvenation and melt extraction processes. *Am. Mineral.* **102**, 766–776 (2017).

81. A. Nishihara, N. Geshi, H. Naruo, Long-term change of the eruption activities of Sakurajima volcano, Japan, inferred from the fallout tephra deposits. *Front. Earth Sci.* **10**, 988373 (2022).

82. P. J. Reimer, W. E. N. Austin, E. Bard, A. Bayliss, P. G. Blackwell, C. Bronk Ramsey, M. Butzin, H. Cheng, R. L. Edwards, M. Friedrich, P. M. Grootes, T. P. Guilderson, I. Hajdas, T. J. Heaton, A. G. Hogg, K. A. Hughen, B. Kromer, S. W. Manning, R. Muscheler, J. G. Palmer, C. Pearson, J. Van Der Plicht, R. W. Reimer, D. A. Richards, E. M. Scott, J. R. Southon, C. S. M. Turney, L. Wacker, F. Adolphi, U. Büntgen, M. Capano, S. M. Fahrni, A. Fogtmann-Schulz, R. Friedrich, P. Köhler, S. Kudsk, F. Miyake, J. Olsen, F. Reinig, M. Sakamoto, A. Sookdeo, S. Talamo, The IntCal20 Northern Hemisphere radiocarbon age calibration curve (0–55 cal kBP). *Radiocarbon* **62**, 725–757 (2020).

83. G. A. R. Gualda, A. Frontoni, B. M. Wallrich, L. Harmon, S. L. Smithies, G. R. Chiaro, A. S. Pamukçu, An assessment of the quality of microanalysis of silicate glass using scanning electron microscope-based energy dispersive spectrometry (SEM-EDS). EarthArXiv X5KT80 [Preprint] (2025). https://doi.org/10.31223/X5KT80.

84. W. H. E. Day, H. Edelsbrunner, Efficient algorithms for agglomerative hierarchical clustering methods. *J. Classif.* **1**, 7–24 (1984).

85. R. Mojena, Hierarchical grouping methods and stopping rules: An evaluation. *Comput. J.* **20**, 359–363 (1977).

86. L. L. Summa, K. L. Verosub, Trace element mobility during early diagenesis of volcanic ash: Applications to stratigraphic correlation. *Quat. Int.* **13–14**, 149–157 (1992).

87. P. Tollan, B. Ellis, J. Troch, J. Neukampf, Assessing magmatic volatile equilibria through FTIR spectroscopy of unexposed melt inclusions and their host quartz: A new technique and application to the Mesa Falls Tuff, Yellowstone. *Contrib. Mineral. Petrol.* **174**, 24 (2019).

88. Y. Zhang, R. Belcher, P. D. Ihinger, L. Wang, Z. Xu, S. Newman, New calibration of infrared measurement of dissolved water in rhyolitic glasses. *Geochim. Cosmochim. Acta* **61**, 3089–3100 (1997).

89. H. Behrens, S. Ohlhorst, F. Holtz, M. Champenois, CO_2 solubility in dacitic melts equilibrated with H_2O-CO_2 fluids: Implications for modeling the solubility of CO_2 in silicic melts. *Geochim. Cosmochim. Acta* **68**, 4687–4703 (2004).

90. M. S. Ghiorso, B. W. Evans, Thermodynamics of rhombohedral oxide solid solutions and a revision of the Fe-Ti two-oxide geothermometer and oxygen-barometer. *Am. J. Sci.* **308**, 957–1039 (2008).

91. K. Iacovino, S. Matthews, P. E. Wieser, G. M. Moore, F. Bégué, VESIcal Part I: An open-source thermodynamic model engine for mixed volatile (H_2O-CO_2) solubility in silicate melts. *Earth Space Sci.* **8**, e2020EA001584 (2021).

92. J. Crank, *The Mathematics of Diffusion* (Oxford Univ. Press, 1975), 414 pp.

93. E. J. F. Mutch, J. Maclennan, A. L. Madden-Nadeau, The dichotomous nature of Mg partitioning between plagioclase and melt: Implications for diffusion chronometry. *Geochim. Cosmochim. Acta* **339**, 173–189 (2022).

94. R. Dohmen, K. Faak, J. D. Blundy, Chronometry and speedometry of magmatic processes using chemical diffusion in olivine, plagioclase and pyroxenes. *Rev. Mineral. Geochem.* **83**, 535–575 (2017).

95. J. A. Van Orman, D. J. Cherniak, N. T. Kita, Magnesium diffusion in plagioclase: Dependence on composition, and implications for thermal resetting of the 26Al-26Mg early solar system chronometer. *Earth Planet. Sci. Lett.* **385**, 79–88 (2014).

96. F. Costa, S. Chakraborty, R. Dohmen, Diffusion coupling between trace and major elements and a model for calculation of magma residence times using plagioclase. *Geochim. Cosmochim. Acta* **67**, 2189–2200 (2003).

97. Curry, A., Gaynor, S. P., Davies, J. H. F. L., Ovtcharova, M., Simpson, G., & Caricchi, L., Timescales and thermal evolution of large silicic magma reservoirs during an ignimbrite flare-up: Perspectives from zircon. *Contrib. Mineral. Petrol.*, **176**, 103 (2021).
